# Synthesis and *In vitro* evaluation of bichalcones as novel anti-toxoplasma agents

**DOI:** 10.3389/fchem.2024.1406307

**Published:** 2024-07-22

**Authors:** Flaminia Mazzone, Moritz K. T. Klischan, Julian Greb, Sander H. J. Smits, Jörg Pietruszka, Klaus Pfeffer

**Affiliations:** ^1^ Institute of Medical Microbiology and Hospital Hygiene, Heinrich Heine University Düsseldorf, University Hospital Düsseldorf, Düsseldorf, Germany; ^2^ Institute of Bioorganic Chemistry, Heinrich Heine University Düsseldorf at Forschungszentrum Jülich, Jülich, Germany; ^3^ Institute of Biochemistry, Heinrich Heine University Düsseldorf, Düsseldorf, Germany; ^4^ Center for Structural Studies, Heinrich Heine University, Düsseldorf, Germany; ^5^ Institute of Bio- and Geosciences (IBG-1): Biotechnology, Forschungszentrum Jülich GmbH, Jülich, Germany

**Keywords:** *Toxoplasma gondii*, bichalcones, anti-infective, anti-toxoplasma, stereoisomers, biaryl, bioactivity

## Abstract

Toxoplasmosis is a zoonotic disease caused by *Toxoplasma gondii*, an apicomplexan parasite that infects approximately a third of the world’s human population. This disease can cause serious complications during pregnancy and can be fatal in immunocompromised hosts. The current treatment options for toxoplasmosis face several limitations. Thus, to address the urgent medical need for the discovery of novel anti-toxoplasma potential drug candidates, our research focused on exploring a series of monomeric and dimeric chalcones, polyphenolic molecules belonging to the class of flavonoids. Chalcones **1aa**—**1bg** and axially chiral A-A′-connected bichalcones **2aa**—**2bg** were evaluated *in vitro* against the proliferation of the parasite in a cell-based assay. A comparison of the efficacy demonstrated that, in several cases, bichalcones exhibited increased bioactivity compared to their corresponding monomeric counterparts. Among these compounds, a bichalcone with a phenyl substituent and a methyl moiety **2ab** showed the most potent and selective inhibitory activity in the nanomolar range. Both enantiomers of this bichalcone were synthesized using an axially chiral biphenol building block. The biaryl bond was forged using Suzuki cross-coupling in water under micellar catalysis conditions. Separation of the atropisomers of this biphenol building block was conducted by chiral HPLC on a preparative scale. The biological evaluation of the enantiomers revealed that the (*R*_a_)-enantiomer (*R*_a_)-**2ab** is the eutomer. These studies suggest that bichalcones may be important drug candidates for further *in vivo* evaluations for the discovery of anti-toxoplasma drugs.

## 1 Introduction

*Toxoplasma gondii* (*T. gondii*), the causative agent of toxoplasmosis, is a coccidian parasite that belongs to the phylum Apicomplexa (Montoya and Liesenfeld, 2004; Hill et al., 2018). This large phylum includes also other unicellular eukaryotes (Levine, 1988) that survive by infecting a wide range of hosts and cause severe diseases such as malaria [*Plasmodium falciparum* (Phillips et al., 2017)], babesiosis [*Babesia* spp. (Homer et al., 2000)], or cryptosporidiosis [*Cryptosporidium parvum* (Helmy and Hafez, 2022)]. Like all other apicomplexans, *T. gondii* displays a heteroxenous and complex life cycle. It alternates between sexual replication that occurs exclusively in the Felidae family members (the definitive hosts), and asexual replication in a variety of warm-blooded intermediate hosts, including humans, *via* three infectious stages: tachyzoites, bradyzoites, and sporozoites (Dubey et al., 1998; Tenter et al., 2000). Due to its widespread distribution, *T. gondii* is often referred to as one of the most successful parasites (Delgado et al., 2022). Multiple modes of transmission can result in human infections: foodborne, *via* ingestion of contaminated water and raw or undercooked meat; fecal-oral, *via* unintentional ingestion of oocysts from cat feces; and also *via* several minor modes such as congenital transmission and blood or organ transplantation (Dubey, 2021). According to the Centers for Disease Control and Prevention (CDC), it has been estimated that more than 40 million people have been infected with *T. gondii* in the United States alone (CDC, 2024).

In immunocompetent individuals, the infection is often subclinical or asymptomatic in the acute phase, but can trigger behavioral disorder during the latent phase (Fekadu et al., 2010). Without proper treatment, severe disease, or even death, can occur in immunocompromised individuals or fetuses infected congenitally (Wang et al., 2017; Strang et al., 2020). An increased frequency of toxoplasmic encephalitis has been reported in patients with AIDS (Acquired Immunodeficiency Syndrome) with significant immunosuppression (Lejeune et al., 2011).

The current gold-standard treatment for toxoplasmosis relies on the administration of pyrimethamine (PYR) and sulfadiazine (SDZ) (Konstantinovic et al., 2019). Since its discovery in 1953 by Eyles and Coleman (Eyles and Coleman, 1953), the synergistic nature of this combination therapy has been well-established. This synergy is achieved because both drugs interfere with different steps in the folate pathway of the tachyzoite stage of the parasite, and therefore the acute phase of the infection (Sheffield and Melton, 1975). To mitigate the harmful side effects associated with this PYR-SDZ regimen, including its bone marrow myelosuppression, folinic acid (leucovorin) has been included in the combination (Van Delden and Hirschel, 1996; Alday and Doggett, 2017). Unfortunately, these and other current therapies are still burdened with strong side effects that often lead to patient noncompliance and discontinuation of therapy. Moreover, the lack of drugs that specifically target the cyst form of the parasite, which is responsible for chronic and latent infection, remains a critical limitation. Thus, there is an urgent need for the discovery and development of novel, potent, and well-tolerated treatments to overcome these challenges and improve the wellbeing of patients inflicted with toxoplasmosis (Konstantinovic et al., 2019).

Natural products play a critical role in the field of infectious diseases drug discovery (Newman and Cragg, 2020). These compounds usually offer a wide and diverse range of biological activities, but often show only moderate or weak potency in early evaluations. Thus, synthetic work on natural products is important for the identification of natural product analogs as novel hits and lead compounds (Deng et al., 2019).

A very prominent class of natural products are flavonoids. Among these phenylpropanoid-based organic compounds are chalcones, polyphenolic secondary metabolites of plants (Elkanzi et al., 2022). In particular chalcones, with their α,β-unsaturated ketone moiety (Michael system), reveal ubiquitous bioactive structural motifs (Zhuang et al., 2017; Salehi et al., 2020). Synthetic chalcone derivatives thus have been investigated, incorporating various substitutions in the A- and B-rings, to modulate both potency and bioactivity (Mezgebe et al., 2023; Oliveira et al., 2023). These molecules have attracted great interest in medicinal chemistry and are now considered privileged structures for their simple scaffold and their wide variety of pharmacological activities and characteristics (Zhuang et al., 2017), including antibacterial (Dan and Dai, 2020), anti-inflammatory (Vale et al., 2023), antiviral (Fu et al., 2020), anticancer (Ouyang et al., 2021), antioxidant (Bale et al., 2021), antidiabetic (Rocha et al., 2020), and antimalarial (Qin et al., 2020) properties, among others. In addition, several studies have demonstrated the potential of chalcones as novel anti-toxoplasma agents (Si et al., 2018; Touquet et al., 2018; Al-Hilli et al., 2021; Jiang et al., 2022; Ghazzay et al., 2023), but their mode of action on *T. gondii* remains unclear. The α,β-unsaturated ketone moiety is considered the main pharmacophore, due to its reactivity and crucial role in various biological activities (Batovska and Todorova, 2010; Sahu et al., 2012). Loss of the ketone moiety has been shown to result in a significant decrease or absence of bioactivity of chalcones (Matos et al., 2015), which is also true for their anti-toxoplasma activity (Jiang et al., 2022). Thus, several synthetic chalcone derivatives have been investigated, preserving the linkage and incorporating various substitutions in the A- and B-rings to modulate both potency and bioactivity (Mezgebe et al., 2023; Oliveira et al., 2023). Overall, three main strategies are used to derivatize the chalcone scaffold: modification of the aryl A- and B-rings, substitution of the aryl rings with heteroaryl rings, and molecular hybridization by conjugating the chalcone scaffold with biologically active scaffolds to enhance a specific bioactivity (Ouyang et al., 2021). Chalcones have been shown to exhibit enhanced antitubercular, antibacterial, antifungal, and antioxidant activities when their aromatics rings feature electron-withdrawing and/or electron-donating substitutions (Gupta and Jain, 2015; da Silva et al., 2023). Notably, the inclusion of the electron-donating amino group on the A aryl ring and an electron-withdrawing group on the aryl B-ring resulted in particularly higher anti-toxoplasma activity (Jiang et al., 2022).

Bichalcones (also commonly referred to as “bis-chalcones”), a subclass of chalcones and thus of the broader class of flavonoids, have been shown to possess intriguing biological activities, including antiplasmodial activity (Ram et al., 2000; Domínguez et al., 2013; Sharma et al., 2018). Despite this, these molecules have hardly been investigated in comparison to chalcones (Pereira et al., 2023). These observations suggest that further research on bichalcones may reveal valuable insights and novel potential therapeutic applications of these molecules.

**FIGURE 1:**
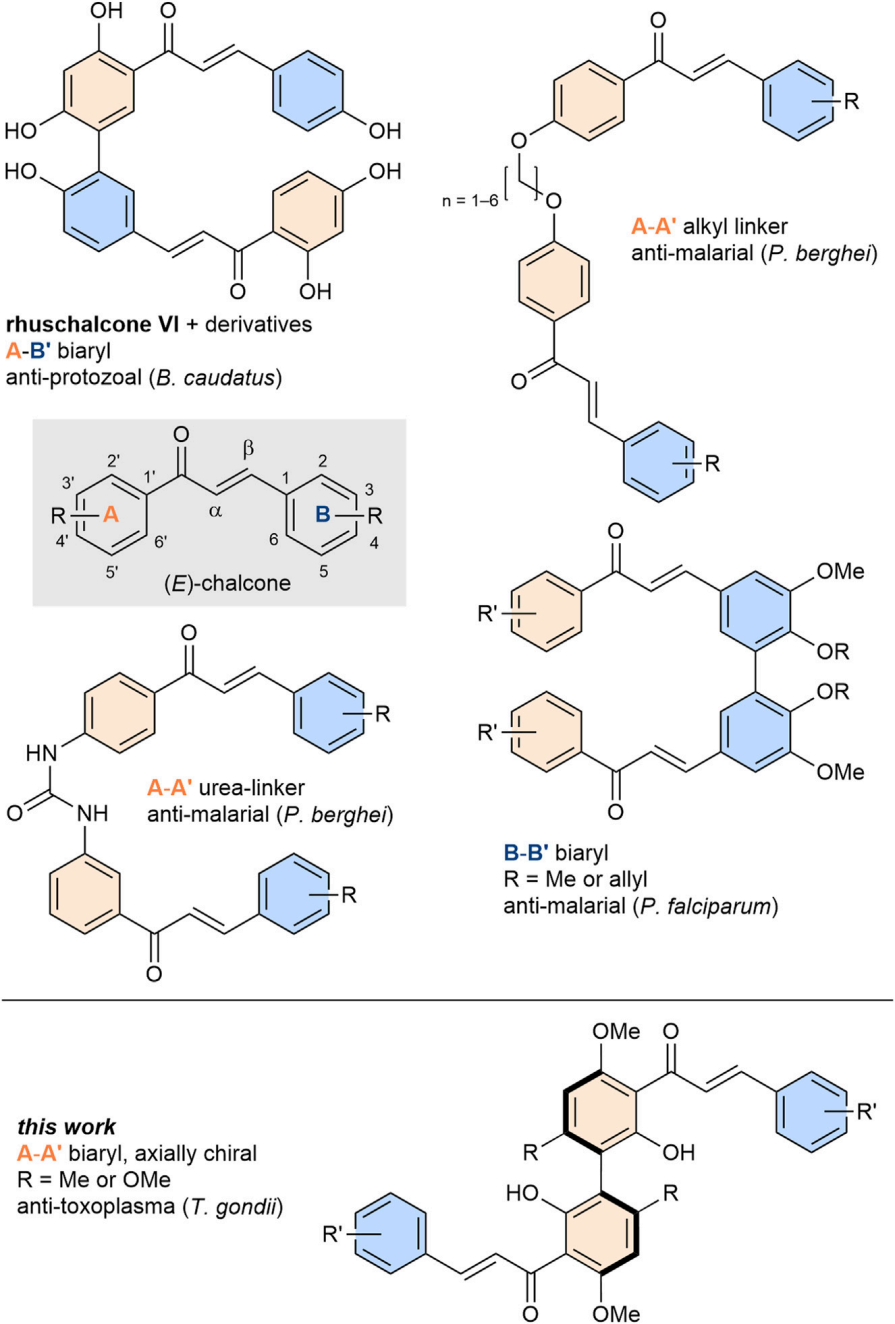
General structure of chalcones and various bichalcones with different linkages and their respective bioactivities. Colors are used to indicate A-rings (orange) and B-rings (blue) respectively for visual support.

Bichalcones exist in a variety of different linkages with respect to the monomeric unit (Figure 1). Depending on their scaffolds, different bioactivities were observed. Common dimeric scaffolds include, among others (Masesane et al., 2000; Mdee et al., 2003; Zhang et al., 2013; Arslan et al., 2016; Zhuang et al., 2017; Karaman et al., 2018; Menezes and Diederich, 2019; Pereira et al., 2023) urea- or alkyl linker compounds with antimalarial activity (Ram et al., 2000; Domínguez et al., 2013), as well as A-B′-type or B-B′-type biaryl bichalcones with antiprotozoal (Mihigo et al., 2010) or antiplasmodial activity (Sharma et al., 2018), respectively. To date, only a few A-A′-bichalcones have been synthesized and their bioactivities have hardly been investigated. A particular challenge in their synthesis is the stereogenic biaryl bond, which makes these compounds axially chiral depending on the *ortho*-substituents of the axis. Lin *et al.* (Lin and Zhong, 1997) and Li *et al.* (Li et al., 1997) independently investigated synthetic routes towards enantiopure 8,8′-biflavones, obtaining enantiopure A-A′-bichalcones as key intermediates. Both strategies involved the tedious use of chiral auxiliaries to access the enantiopure products, limiting the scalability of these approaches.

**SCHEME 1:**
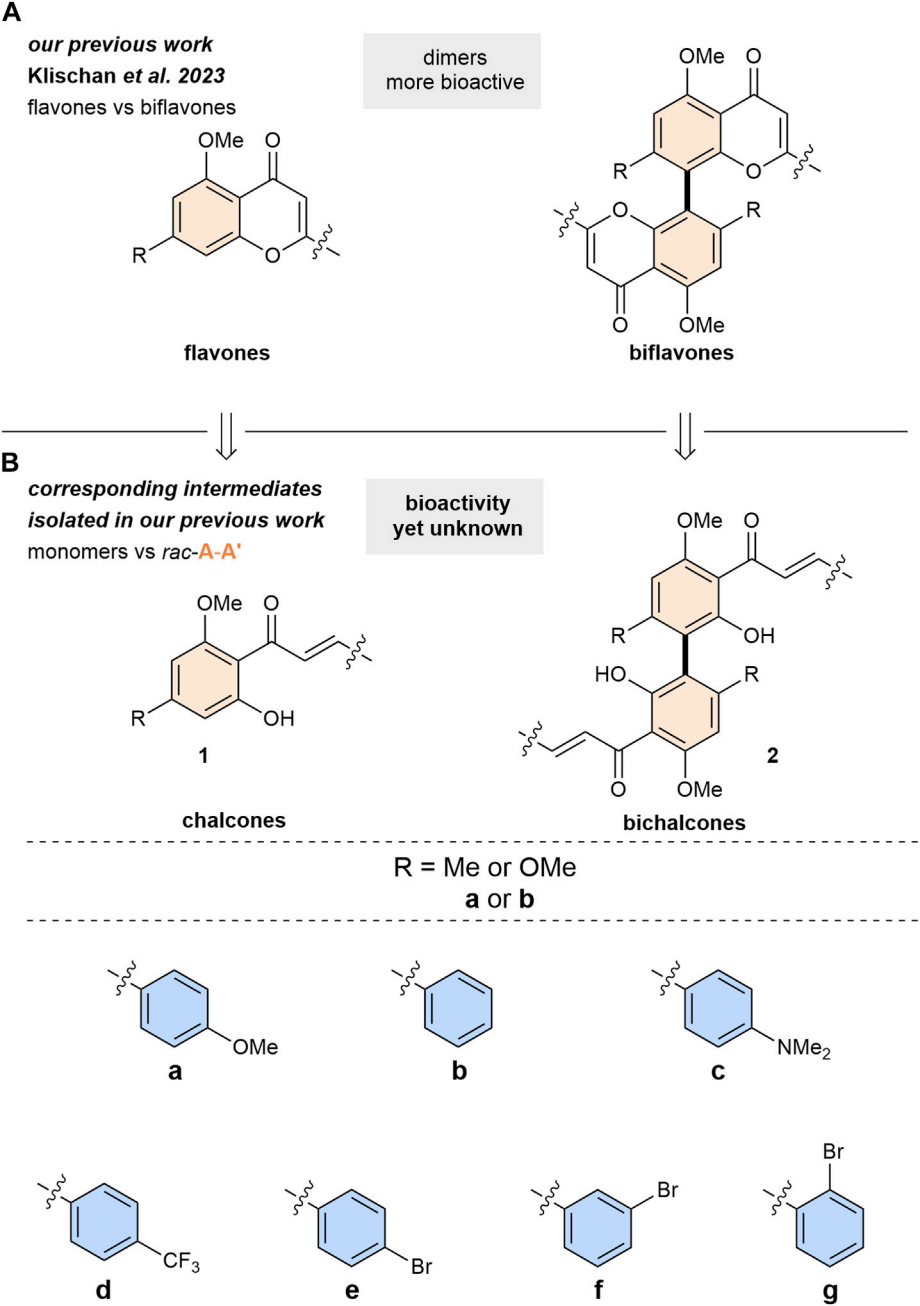
Library of chalcones and bichalcones. For example: bichalcone **(2)** with R = Me **(a)** phenyl substitution at the B-ring **(b)** = **2ab**. Colouring of the rings in accordance with Figure 1.

In our previous study we investigated the anti-toxoplasma activities of various flavones and biflavones (Klischan et al., 2023; Klischan et al., 2024) (Scheme 1A). There, chalcones **1** and racemic A-A′-bichalcones **2** were obtained as intermediate products (Scheme 1B). However, the biological activity profiles of these compounds were not yet investigated. In this work, we report the anti-toxoplasma activities for this readily available library of simplified natural product analogues. Additionally, following a complementary synthetic strategy both enantiomers of the most active A-A′-bichalcone were synthesized and the first biological evaluation of this elusive class of flavonoids was conducted. Their *in vitro* anti-toxoplasma activities were evaluated against the *T. gondii* type II ME49 strain and compared with their monomer chalcone counterparts.

## 2 Materials and methods

### 2.1 Synthesis of chalcones and bichalcones

The chalcones and racemic bichalcones investigated in the present study have been synthesized following established procedures from our previous investigations on the bioactivity of biflavones (Klischan et al., 2023; Klischan et al., 2024). An overview of these syntheses as well as synthetic details, methodologies, and analytical data of the enantioselective synthetic sequence developed in this work are available in the Supplementary Material.

### 2.2 Parasite and cell culture

The tachyzoite stage of the ME49 strain type II of *T. gondii* (ATCC/LGC Standards GmbH, Wesel, Germany, #50611) was maintained and propagated by regular passages infecting monolayers of human foreskin fibroblasts Hs27 (ATCC/LGC Standards GmbH, Wesel, Germany, #CRL-1634), in 25 cm^2^ cell culture flasks, with 5 × 10^6^ tachyzoites. Cultures were grown in Iscove’s Modified Dulbecco’s medium (IMDM; Gibco-Thermo Fisher Scientific, Braunschweig, Germany, #12440053) supplemented with 10% heat-inactivated fetal bovine serum (FBS Standard; South America origin, foetal bovine serum, 2 µm sterile filtered, PAN-Biotech, Aidenbach, Germany, #P30-3,306) and 50 mM 2-mercaptoethanol (Gibco-Thermo Fisher Scientific, Braunschweig, Germany, #21985023) at 37°C and 5% CO_2_ for 72 h. After incubation, the cell culture supernatant was centrifuged at 700 rpm for 5 min. The parasite density was then measured using a hemocytometer and adjusted accordingly to *in vitro* experimental infection analysis.

### 2.3 Compounds for *in vitro* analysis

Pyrimethamine, staurosporine, and all synthesized chalcones and bichalcones were dissolved in DMSO (dimethyl sulfoxide, ≥99%, Thermo Scientific Chemicals, Braunschweig, Germany, #A12380.36) as 10 mM stock solutions and stored at −20°C. Before use, these solutions were thawed and diluted in culture medium to obtain the appropriate concentrations (ranging from 200 μM to 0.02 µM).

### 2.4 *Toxoplasma gondii in vitro* inhibition assay

To evaluate the efficacy of the investigated chalcones and bichalcones against *T. gondii* proliferation, we conducted *in vitro* inhibition assays using a similar protocol as previously reported (Merkt et al., 2021; Mazzone et al., 2022; Klischan et al., 2023). Briefly, we seeded 3 × 10^4^ Hs27 cells in 96-well microtiter plates with a total culture volume of 200 µL per well. To avoid edge effects, only the inner 60 wells of each plate were used. After harvesting *T. gondii* as described previously, the host cells were infected with 3 × 10^4^ tachyzoites per well at a multiplicity of infection (MOI, parasite/host cell) ratio of 1:1. Simultaneously, the compounds were added to the cultures at various concentrations (ranging from 50 μM to 0.02 µM) as described previously. Controls included pyrimethamine (Alday and Doggett, 2017; Konstantinovic et al., 2019) (Merck, Darmstadt, Germany, #219864), *T. gondii* infected cells only, and human interferon γ (IFN γ) (Pfefferkorn, 1986) (Merck, Darmstadt, Germany, #I17001) pre-stimulated cells (300 U/mL for 24 h) which were subsequently infected with *T. gondii*. To quantify *T. gondii* proliferation, the parasites were labeled with tritiated uracil (^3^H-U; 5 mCi, Hartmann Analytic, Braunschweig, Germany, #ART1782, diluted 1:30) (Pfefferkorn and Pfefferkorn, 1977) after 48 h incubation and incubated for another 28–30 h. Prior to assay evaluation, the 96-well microtiter plates were frozen at −20°C. The plates were then thawed at room temperature, cells were extracted using a harvester (Basic96 Harvester; Zinsser Analytic, Skatron Instruments, Northridge, CA, United States) and transferred to glass-fiber filters (Printed Filtermat A 102 mm × 258 mm; PerkinElmer, Waltham, MA, United States). The filters were then dried in a cabinet at 130°C for 20 min, wetted in 10 mL of scintillation solution (Betaplate Scint; PerkinElmer, Waltham, MA, United States, #1205–440), and sealed with plastic covers (Sample Bag for Betaplate; PerkinElmer, Waltham, MA, United States, #1205–441). The filters were clamped into cassettes and analyzed using a beta-counter (Betaplate Liquid Scintillation Counter 1,205; LKB-WALLAK, Melbourne, Australia) to quantify the amount of radioactive uracil in *T. gondii* RNA. IC_50_ values (minimum concentration of compounds required for 50% inhibition *in vitro*) were determined by nonlinear regression analysis using GraphPad PRISM™ statistical software (version 9.5.1; San Diego, CA).

### 2.5 Cytotoxicity assay

The MTT [3-(4, 5-dimethylthiazole-2-yl)-2, 5-diphenyltetrazolium bromide] reduction assay (Mosmann, 1983) was used to quantify the cytotoxic effects of the examined chalcones and bichalcones on the host cells. To avoid edge effects, only the inner 60 wells of each plate were used. Briefly, 5 × 10^4^ Hs27 cells per well were cultured in 96-well microtiter plates in Iscove’s modified Dulbecco’s medium (IMDM, Gibco–Thermo Fisher Scientific, Braunschweig, Germany, #12440053) with a volume of 100 µL per well and incubated overnight at 37°C until confluence. Different concentrations of the compounds (ranging from 200 μM to 0.09 µM) were then added to the Hs27 cells. Controls included untreated Hs27 cells treated with DMSO, and cells treated with staurosporine (0.031, 0.062, 0.125, 0.25, 0.5, 1 µM) (Merck, Darmstadt, Germany, #S4400), a natural product known for its potent activity as apoptosis inducer (Belmokhtar et al., 2001). After 24 h of incubation, the culture media were replaced with 100 µL of Dulbecco’s Modified Eagle Medium (DMEM) medium without red phenol (Gibco-Thermo Fisher Scientific, Braunschweig, Germany, #21041025) plus 10% heat-inactivated fetal bovine serum (FBS Standard, South America origin, fetal bovine serum, 2 µm sterile filtered, PAN-Biotech, Aidenbach, Germany, #P30-3,306), and 50 mM 2-mercaptoethanol (Gibco-Thermo Fisher Scientific, Braunschweig, Germany, #21985023). The experiment was performed following the manufacturer’s instructions (CyQuant MTT Cell Viability Assay Kit, Thermo Fisher Scientific, Braunschweig, Germany, #V-13154). Optical density (O.D.) was measured at 570 nm using a microplate reader (TECAN Sunrise, Männedorf, Switzerland). The half-maximal cytotoxic concentration (CC_50_) values of each compound against Hs27 cells relative to DMSO-treated samples were determined using GraphPad PRISM™ statistical software (version 9.5.1; San Diego, CA). In addition, the selectivity index (SI) of each compound was calculated from the ratio of CC_50_/IC_50_.

## 3 Results

### 3.1 Bichalcones and chalcones are effective inhibitors of *Toxoplasma gondii* tachyzoite growth

In view of previous studies demonstrating the effectiveness of chalcones against apicomplexans (Ram et al., 2000; Domínguez et al., 2013; Sharma et al., 2018; Si et al., 2018; Touquet et al., 2018; Qin et al., 2020; Al-Hilli et al., 2021; Jiang et al., 2022; Ghazzay et al., 2023), we evaluated and compared the activity of 14 chalcones **1** and 14 of their dimeric bichalcone **2** counterparts (Scheme 1B). All chalcones **1** and racemic bichalcones **2** were characterized and reported in our previous investigation (Klischan et al., 2023). An overview of the employed synthetic procedures is provided in Supplementary Scheme S1. To determine their potential against *T. gondii* ME49 tachyzoites, we performed an *in vitro* proliferation assay based on the uptake of radioactively labeled ^3^H-uracil (Pfefferkorn and Pfefferkorn, 1977). We then evaluated their IC_50_ values. As shown in Table 1 and Supplementary Figure S23, chalcones and bichalcones exhibited bioactivity against *T. gondii* proliferation.

**TABLE 1:** *In vitro* activity of chalcones (1aa–bg) and bichalcones (2aa–bg) against *Toxoplasma gondii* ME49 tachyzoites. Values shown in the table represent the means of three independent experiments each done in duplicate (n = 6) ± S.D.

Chalcone	IC_50_ ± S.D. (µM)	Bichalcone	IC50 ± S.D. (µM)
**1aa**	21.81 ± 3.07	**2aa**	6.63 ± 0.77
**1ab**	14.05 ± 2.62	**2ab**	0.11 ± 0.02
**1ac**	>50	**2ac**	>50
**1ad**	>50	**2ad**	24.74 ± 3.52
**1ae**	10.60 ± 1.05	**2ae**	7.91 ± 1.14
**1af**	6.18 ± 0.55	**2af**	30.48 ± 7.21
**1ag**	>50	**2ag**	18.44 ± 0.10
**1ba**	8.08 ± 2.06	**2ba**	>50
**1bb**	6.01 ± 2.54	**2bb**	9.75 ± 1.95
**1bc**	>50	**2bc**	>50
**1bd**	>50	**2bd**	3.09 ± 0.15
**1be**	>50	**2be**	8.55 ± 1.28
**1bf**	11.32 ± 0.61	**2bf**	12.4 ± 2.09
**1bg**	4.46 ± 0.46	**2bg**	>50

Especially, bichalcone **2ab** showed an IC_50_ of 0.11 µM, more potent than its monomeric counterpart (**1ab**, 14.05 µM) by two orders of magnitude and, overall, more active than all other compounds in our dedicated library (3.09–30.48 µM). Bichalcone **2ab** contains a phenyl moiety as the B-ring and a methyl substituent at the A-ring’s 4-position (R = Me) (Scheme 1). Interestingly the latter seems to be important for the anti-toxoplasma activity, since **2bb** with a methoxy group (R = OMe) in the same position showed a comparatively low IC_50_ of 9.75 µM (Table 1; Supplementary Figure S23).

### 3.2 Bichalcone 2ab is a strong and selective inhibitor of *Toxoplasma gondii*

To investigate the selectivity of our (bi)chalcone library regarding parasite inhibition versus human host cell cytotoxicity, we assessed their cytotoxic potential in human fibroblasts Hs27 with the MTT reduction assay. The screening revealed weak to no cytotoxicity for chalcones and no cytotoxicity for all bichalcones (Table 2; Supplementary Figure S24). Compound **2ab** had the highest selectivity index (SI) of >6,994, indicating low cytotoxicity against human fibroblasts and high activity against *T gondii*. Therefore, due to its high selectivity, it was selected as our lead compound for further investigation.

**TABLE 2:** *In vitro* cytotoxicity of chalcones (1aa–bg) and bichalcones (2aa–bg) on human fibroblasts Hs27 and their selectivity indexes (SI) Values shown in the table represent the means of three independent experiments each done in duplicate (n = 6).

Chalcone	CC50 (µM)	SI	Bichalcone	CC_50_ (µM)	SI
**1aa**	179.7 ± 32	8.2	**2aa**	>200	>30
**1ab**	132.8 ± 28	13.2	**2ab**	>200	>6,994
**1ac**	>200	–	**2ac**	>200	–
**1ad**	>200	–	**2ad**	>200	>8
**1ae**	190.3 ± 99	18	**2ae**	>200	>25
**1af**	133.8 ± 78	21.7	**2af**	>200	>7
**1ag**	>200	–	**2ag**	>200	>11
**1ba**	>200	>25	**2ba**	>200	–
**1bb**	87.8 ± 16	14.6	**2bb**	>200	>21
**1bc**	>200	–	**2bc**	>200	–
**1bd**	>200	–	**2bd**	>200	>65
**1be**	>200	–	**2be**	>200	>23
**1bf**	>200	>18	**2bf**	>200	>16
**1bg**	86.5 ± 72	29.8	**2bg**	>200	–

### 3.3 Enantiopure synthesis of (*S*_a_)- and (*R*_a_)-2ab enantiomers

**SCHEME 2:**
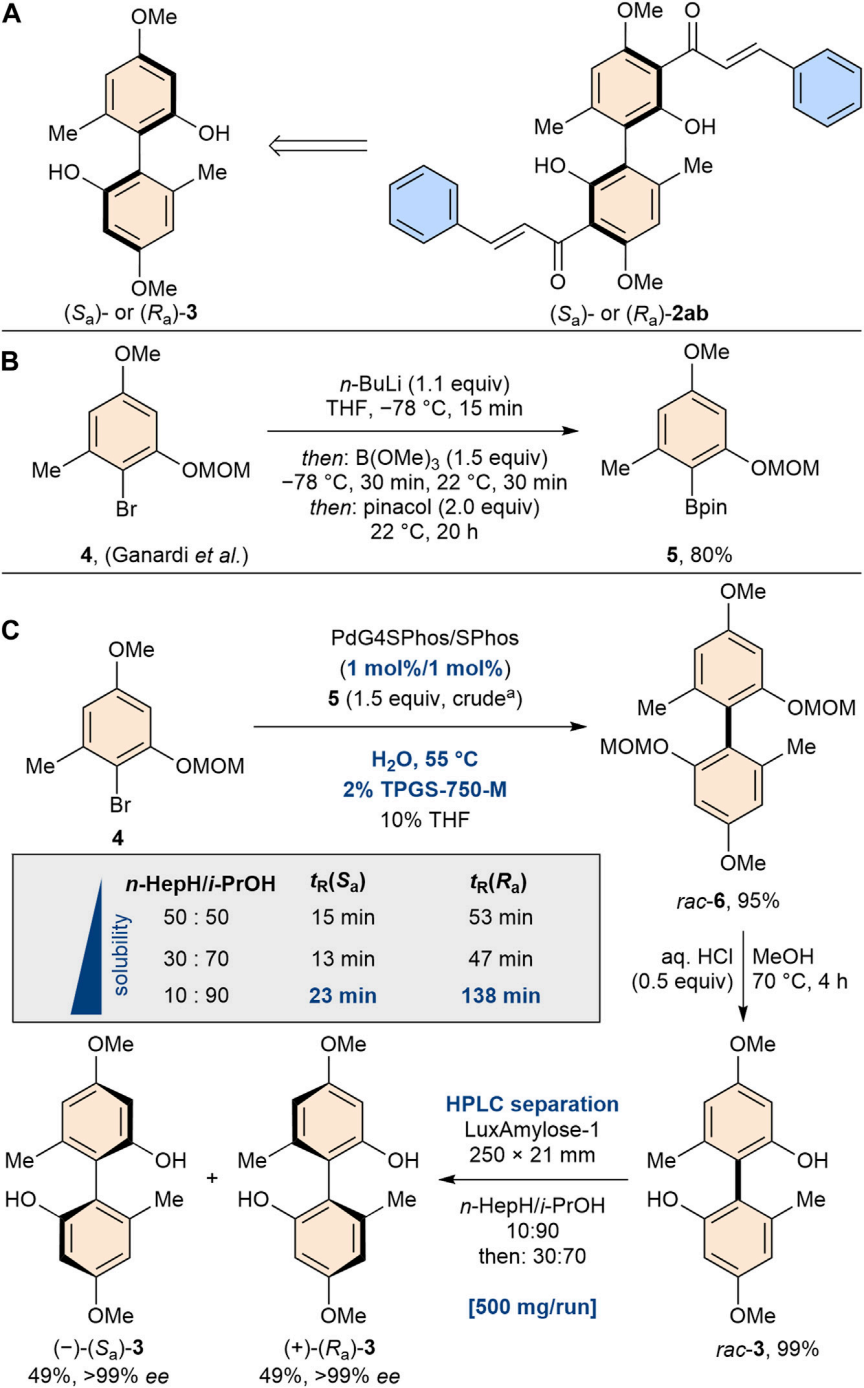
**(A)** Synthesis strategy of both enantiomers of bichalcone **2ab**. **(B)** Scalable borylation of readily available brominated starting material **4**. **(C)** Pd-catalyzed (1 mol% Pd) Suzuki cross coupling using amphiphile TPGS-750-M. Separation of the enantiomers was achieved by HPLC because of drastically different elution times (Δ*t*_R_ = 115 min for *n*-heptane: *i*-propanol 10:90 v/v) for both enantiomers using Lux i-Amylose-1.

Since **2ab** exhibits axial chirality and only its racemic mixture has been studied, we turned our attention to the investigation of the pure enantiomers of **2ab**. The aim of this investigation was to discern potential variations in activity and selectivity between the enantiomers. To gain access to both atropisomers of enantiopure bichalcones, our synthetic strategy involved the use of biphenol **3**, a building block used in previous studies for the synthesis of various natural product analogs (Ganardi et al., 2023; Greb et al., 2023) (Scheme 2). The readily available brominated starting material **4** was converted to the corresponding boronic acid ester **5** in a scalable manner via a halogen-metal exchange followed by treatment with trimethyl borate and pinacol. Next, the aryl-aryl bond formation was investigated. The sterically demanding tetra-*ortho*-substituted biaryl bond of **6** was constructed by Suzuki cross-coupling. After screening various conditions (Supplementary Material), we were able to exploit the use of Lipshutz’s TPGS-750-M amphiphile in water under micellar catalysis conditions (Lipshutz et al., 2011), yielding *rac*-**6** in 95% yield and drastically reducing the amount of palladium required [compared to our previous protocol (Ganardi et al., 2023)]. Having acidically deprotected *rac*-**6**, we then needed to access enantiomerically pure biphenol **3**. To our delight, we found that the separation of both enantiomers was possible by preparative normal phase HPLC using Lux i-Amylose-1 as the column. Due to the unusually good separation performance and high solubility in the polar elution medium, this process could be scaled up to 500 mg per run yielding (*S*_a_)-**3** and (*R*_a_)-**3** in 49% each, exceeding the scale of previously established synthesis protocols for enantiopure bichalcones (Li et al., 1997; Lin and Zhong, 1997).

**SCHEME 3:**
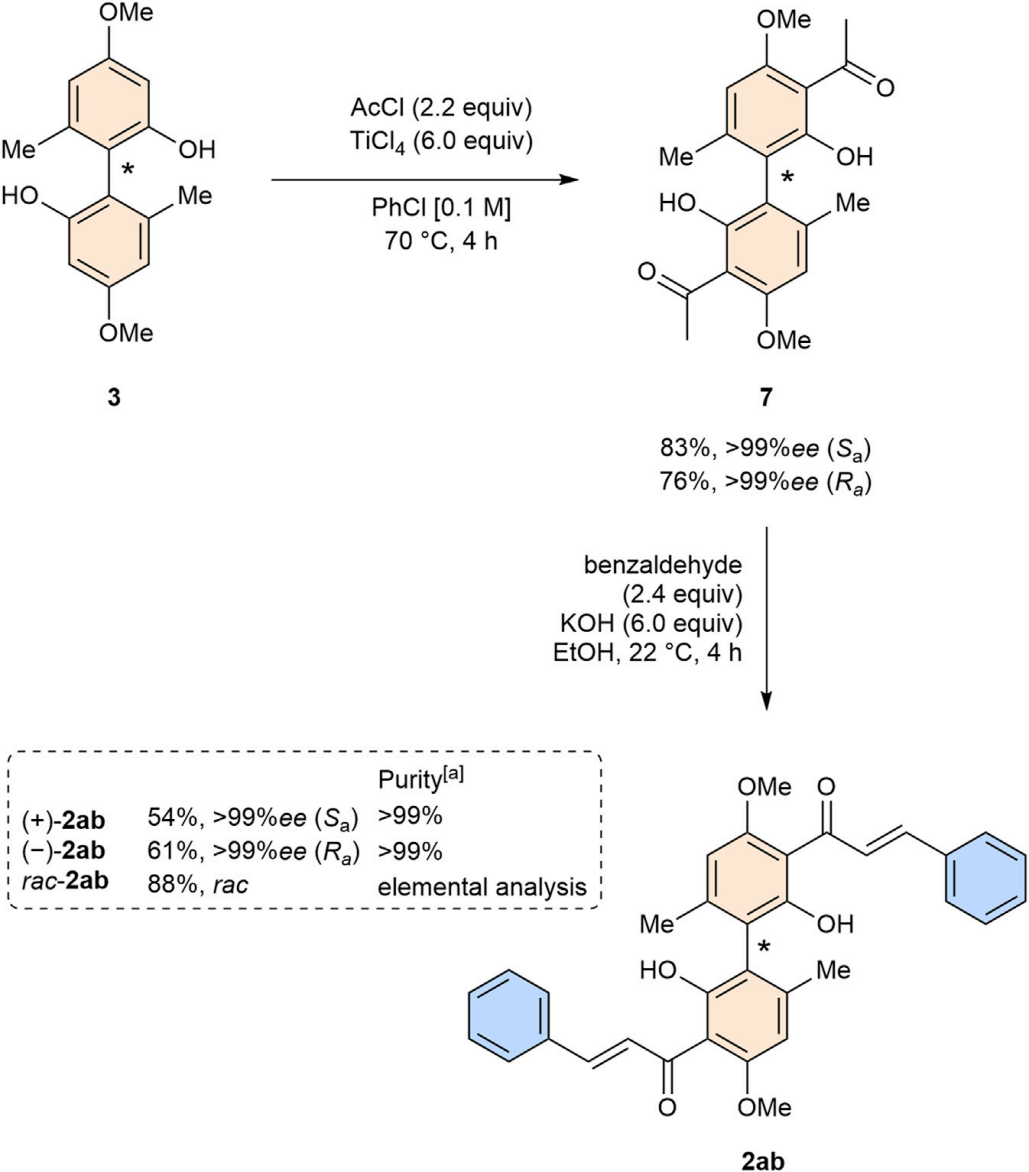
Synthesis of both enantiomers of bichalcone **2ab**. (a) Purity by normal phase and reversed phase HPLC.

With both enantiomers of biphenol **3** in hand, we focused our efforts on the synthesis of both enantiomers of bichalcone **2ab**. The synthesis of acetophenone **7**, following our previously established protocol (Greb et al., 2023), proceeded smoothly for both enantiomers in yields of 83% (*S*_a_) and 76% (*R*_a_), respectively (Scheme 3). We proceeded with the synthesis of both enantiomers of bichalcone **2ab**. Racemic bichalcone **2ab** was conveniently obtained from our previous investigations on the bioactivity of biflavones (Klischan et al., 2023). In contrast to the racemic mixture of **2ab** isolation by column chromatography was feasible. Both enantiomers were obtained in yields of 54% (*S*_a_) and 61% (*R*_a_), respectively, with >99%*ee* each as determined by HPLC. We attribute the lower yield compared to the racemic mixture to increased formation of flavanone side products.

### 3.4 Biological evaluation of the enantiomers of bichalcone **2ab**

After the successful synthesis of enantiopure (*S*_a_)-**2ab** and (*R*_a_)-**2ab**, we performed a comparative evaluation of their activity against *T. gondii* tachyzoites and their potential cytotoxicity on Hs27 cells to determine if their absolute configuration has an effect on the observed biological activity. As shown in Table 3 and Supplementary Figure S25, both enantiomers are highly active against *T. gondii* proliferation with IC_50_ values in the nanomolar range and no cytotoxicity on Hs27, similar to the racemic mixture. Interestingly (*R*_a_)-**2ab** was found to be the eutomer (more potent enantiomer) with an IC_50_ of 0.10 µM, less than half that of the enantiomer (*S*_a_)-**2ab**, and slightly more active than the racemic mixture. A comparison with PYR–the gold-standard treatment for toxoplasmosis (Konstantinovic et al., 2019)—[IC_50_ of 0.22 µM, literature 0.4 µM (Touquet et al., 2018)] highlights the significance of our findings (Table 3).

**TABLE 3:** *In vitro* activity (IC_50_) against *Toxoplasma gondii*, cytotoxicity (CC_50_) on human Hs27 cells of (*S*_a_)-2ab (*R*_a_)-2ab, *rac-*2ab, and pyrimethamine (PYR) Values shown in the table represent the means of three independent experiments each done in duplicate (*n* = 6) ± S.D.

Compound	IC_50_ (µM)	CC_50_ (µM)
(*S*_a_)-**2ab**	0.31 ± 0.03	>200
(*R*_a_)-**2ab**	0.10 ± 0.01	>200
*rac*-**2ab**	0.11 ± 0.02	>200
PYR	0.22 ± 0.05	>100

### 3.5 ADME prediction of bichalcone **2ab**

To additionally evaluate the pharmacokinetic properties of the tested compounds and to assess their drug-likeness, corresponding metrics were calculated using SwissADME (Daina et al., 2017; Bakchi et al., 2022). Table 4 shows a subset of key parameters for selected compounds from these predictions. Among the bichalcones, the most active compound **2ab** is indeed the smallest molecule and also has the lowest topological polar surface area (TPSA). In addition, **2ab** has the least number of rotatable bonds, which results in it being the only bichalcone with no violations of Veber’s rules for oral bioavailability (Veber et al., 2002) while at the same time showing some solubility and only one violation of Lipinski’s rule of five (Lipinski et al., 2001). Compared to the monomeric chalcones (**1aa** and **1bb** are shown as representatives), **2ab**, as well as all other bichalcones, are predicted to show relatively low gastrointestinal (GI) absorption and no blood-brain barrier (BBB) permeation. Interestingly, **2ab** is not a substrate of P-glycoproteins (P-gp), transporters belonging to the ATP-binding cassette (ABC) superfamily, that actively efflux small molecules through biological membranes reducing drug accumulation in the GI tract and in the brain by enhancing their elimination (Chen et al., 2018). Furthermore, bichalcone **2ab** is predicted to not inhibit any of the cytochrome P450 isoforms, critical metabolic enzymes responsible for drug biotransformation (Zhao et al., 2021). Inhibition of these enzymes can lead to drug-drug interactions (Deodhar et al., 2020). Therefore, bichalcones show overall acceptable drug-like properties, with **2ab** fulfilling the most criteria of the bichalcones tested.

**TABLE 4:** Pharmacokinetics and drug-likeness predictions for selected chalcones and bichalcones. Predictions were performed using the SwissADME webtool (Daina et al., 2017; Bakchi et al., 2022). The detailed dataset of predicted property data for all chalcones and bichalcones is given in Supplementary Tables S9–S19.

Comp	TPSA Å2	Solubility	GI absorp	BBB perm	P-gp substrate	CYP2C9 inhib	Lipinsiki #violat	Veber #violat
**1ab**	46.5	moderate	high	yes	no	yes	0	0
**1bb**	55.8	moderate	high	yes	no	yes	0	0
**2ab**	93.1	poor	low	no	no	no	1	0
**2ac**	99.5	poor	low	no	no	no	1	1
**2ae**	93.1	insoluble	low	no	no	no	2	0
**2bb**	111.5	poor	low	no	no	no	1	1

## 4 Discussion

Toxoplasmosis, the disease caused by the apicomplexan parasite *T. gondii*, is the most common infection worldwide, affecting virtually all warm-blooded animals, including humans. Currently available treatments are only able to control the acute infection caused by the actively replicating tachyzoite stage, while having little or no effect on the chronic infection caused by the slowly replicating bradyzoite stage. Furthermore, more than 60 years after its discovery, the combination therapy of pyrimethamine-sulfadiazine (PYR-SDZ) remains the frontline treatment and only few new drugs have been approved for the treatment of toxoplasmosis in humans in the meantime (Dunay et al., 2018).

Previous studies have demonstrated the potential of flavonoids as novel anti-toxoplasma entities *in vitro* and *in vivo*, including flavones (Abugri et al., 2018; Klischan et al., 2023) and chalcones (Si et al., 2018; Touquet et al., 2018; Qin et al., 2020; Al-Hilli et al., 2021; Jiang et al., 2022; Ghazzay et al., 2023).

We have recently shown that biflavones are more potent than their monomeric counterpart, for both their antioxidant capacity and anti-toxoplasma *in vitro* activity (Klischan et al., 2023). Therefore, the objective of the present study was to explore the anti-toxoplasma *in vitro* activity of chalcones, key synthetic intermediates of flavones obtained in our previous study. With a special focus on the biological comparison between monomers and dimers, this study aims to demonstrate the potential of the less investigated bichalcone counterparts.

Comparison of the *in vitro* anti-toxoplasma potential of monomers and their axially chiral dimeric counterparts revealed that bichalcones are in some cases more potent than chalcones as was also observed for flavones and biflavones in our previous study (Klischan et al., 2023). In addition, all bichalcones had no cytotoxic effect on human fibroblasts Hs27, thus possessing a better cytotoxic profile than their monomeric counterparts. Bichalcone **2ab** exhibits the highest potency and selectivity, being the only one with an IC_50_ in the nanomolar range. Additionally, predictions of the ADME properties identified **2ab** to be rather drug-like thus overall having the potential to be a novel anti-toxoplasma lead compound.

Having identified a highly active racemic compound, we then established the synthesis of both enantiomers of **2ab**. In addition to our previous racemic synthesis strategy, we successfully established a complementary atropselective access starting from enantiopure biphenol **3**. Synthesis of *rac*-**3** was successfully performed using low amounts of palladium catalyst enabled by an amphiphilic additive. We then separated both enantiomers using chiral HPLC on preparative scale. Finally, both enantiomers of the most active bichalcone **2ab** were obtained in satisfactory yields and excellent purity. Comparison of the activity of the two enantiomers (*S*_a_)-**2ab** and (*R*_a_)-**2ab** showed that stereochemistry plays a key role in potency, leading to the identification of (*R*_a_)-**2ab** as the eutomer. In addition, a comparison of the activity with the activity of PYR demonstrated that the enantiomer (*R*_a_)-**2ab** and the racemic mixture are more potent than the anti-toxoplasma gold standard treatment.

Interestingly, Karaman et al. (2018) performed a structure-based virtual screening to identify of novel sirtuin inhibitors and modulators. Docking studies identified two bichalcones, a rhuschalcone IV and a rhuschalcone I analog, as active inhibitors of human SIRT-1 and SIRT-2 (Karaman et al., 2018). Sirtuins are highly conserved nicotinamide adenine dinucleotide (NAD^+^)-dependent enzymes. In humans, seven sirtuins are classified as class III histone deacetylases (HDACs) that regulate a wide variety of important intracellular activities, such as metabolism, transcription, and genome stability (Finkel et al., 2009; Abbotto et al., 2022). The *T. gondii* genome encodes a SIR2 subtype homolog (class III) (Yu et al., 2021a; Yu et al., 2021b). The function of SIR2-like proteins in apicomplexans has not been fully elucidated, although they have been shown to be involved in the epigenetic regulation of virulence genes essential for *P. falciparum* pathogenesis and persistence (Religa and Waters, 2012). Therefore, we performed a docking study of (*S*_a_)-**2ab** and (*R*_a_)-**2ab** with the *Tg*SIR2 homolog to explore whether it could be a potential target of the bichalcones. Unfortunately, the docking studies did not result in a stable protein-ligand complex that could explain the *T. gondii* growth inhibitory properties. Since these are *in silico* experiments, there may be several reasons to explain this, but first, initial *in vitro* binding studies need to be performed to conclusively suggest that this *Tg*SIR2 protein is indeed the target of bichalcones. Thus, further research is needed to elucidate the mechanism of action and to identify the target of **2ab** in *T. gondii*, in particular to investigate the binding interaction with the two enantiomers of **2ab**. In addition, *in vivo* studies are needed to explore their activity and safety in a mouse model system in the future.

## Data Availability

The original contributions presented in the study are included in the article/Supplementary Material, further inquiries can be directed to the corresponding authors.
