# Supplementary material for: Synthesis and In vitro evaluation of bichalcones as novel anti-toxoplasma agents
Source: Front Chem. 2024 Jul 22;12:1406307. doi: 10.3389/fchem.2024.1406307 (PMC11298430; doi:10.3389/fchem.2024.1406307)
Supplement: Supplementary file 1 [file DataSheet1.pdf]

Supplementary Material

Synthesis and *In Vitro* Evaluation of Bichalcones as Novel Anti-Toxoplasma Agents

Flaminia Mazzone[1] †, Moritz K. T. Klischan[2] †, Julian Greb[2] †, Sander H. J. Smits[3,4], Jörg Pietruszka[2,5*] and Klaus Pfeffer[1*]

[1]Institute of Medical Microbiology and Hospital Hygiene, Heinrich Heine University Düsseldorf, University Hospital Düsseldorf, Düsseldorf, Germany

[2]Institute of Bioorganic Chemistry, Heinrich Heine University Düsseldorf at Forschungszentrum Jülich, Jülich, Germany

[3]Institute of Biochemistry, Heinrich Heine University Düsseldorf, Düsseldorf, Germany

[4]Center for Structural Studies, Heinrich Heine University, Düsseldorf, Germany

[5]Institute of Bio- and Geosciences (IBG-1): Biotechnology, Forschungszentrum Jülich GmbH, Jülich, Germany

†These authors share first authorship

* Correspondence:

Klaus Pfeffer

klaus.pfeffer@hhu.de

Jörg Pietruszka

j.pietruszka@fz-juelich.de

1 Experimental Procedures

1.1 Chalcone synthesis

The synthesis of the library of chalcones **1** and bichalcones **2** was reported in our previous work (Klischan et al., 2023) (**Scheme S1**). Commercially available phenols **9a** (R^1= Me) and **9b** (R^1 = OMe) were transformed to the corresponding acetophenones (**10a** and **10b**) and subsequently methyl protected and isolated (**11a** and **11b**). A Claisen condensation with benzaldehydes **12a** – **g** provided chalcones **1aa** – **1bg**. Solid phase Fe-mediated oxidative coupling of **10** enabled access to acetophenone dimers **7a** and **7b**. Again, Claisen Schmidt condensation with benzaldehydes **12a** – **g** resulted in the isolation of bichalcones **2aa** – **2bg**. The characterization data can be found in our previously reported synthesis (Chalcones **1aa** – **1bg** in this work refer to **6aa** – **6bg** in (Klischan et al., 2023) and bichalcones **2aa** – **2bg** in this work refer to **7aa** – **7bg** in (Klischan et al., 2023)).

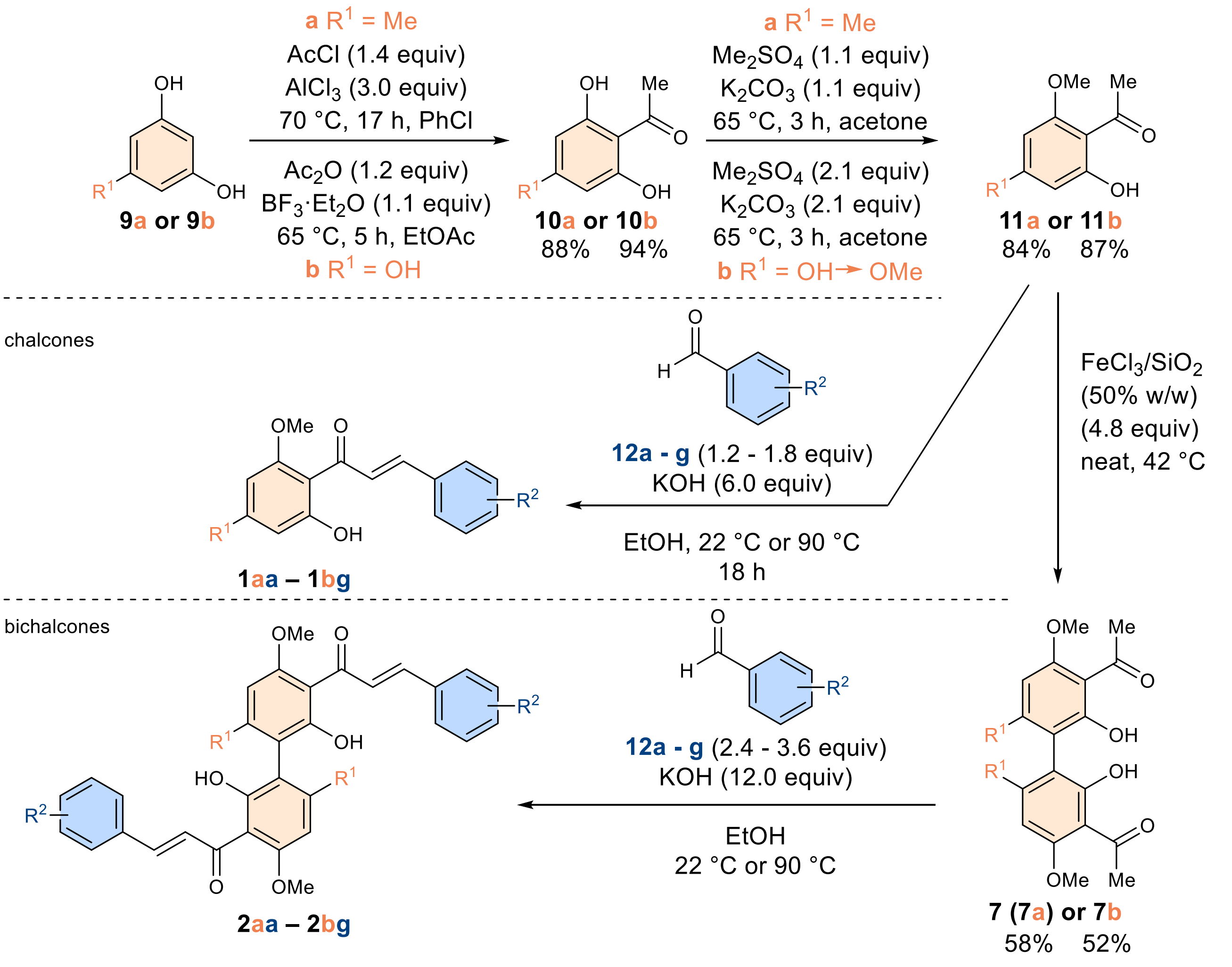

Scheme S1: Synthesis scheme of chalcones and bichalcones (Klischan et al., 2023). Colours indicate the numbering system of the compounds.

1.2 General Information

Prior to use in synthesis benzaldehyde was washed with saturated aqueous Na_2CO_3 solution, dried over $MgSO_4$ and then isolated by vacuum distillation ($1 \cdot 10^{-1}$ mbar) at 94 °C (58 °C head temperature).

1.3 Optimization of Suzuki Coupling in Micellar Aqueous Media and HPLC separation

The required biphenol building block **3** can be formed via double methoxymethyl (MOM)-protected intermediate **6**. While the synthesis of such tetra-*ortho*-substituted biaryls can be challenging, efficient syntheses have been developed recently (**Scheme S2**). Besides formation via oxidation of the corresponding biaryl Lipshutz cuprates, a one-pot Miyaura borylation Suzuki coupling (MBSC) sequence has been developed. While being scalable and efficient, these approaches either involved the use of superstoichiometric amount of pyrophoric *tert*-butyllithium and toxic copper cyanide or rather high effective palladium loadings relative to the amount of isolated product.

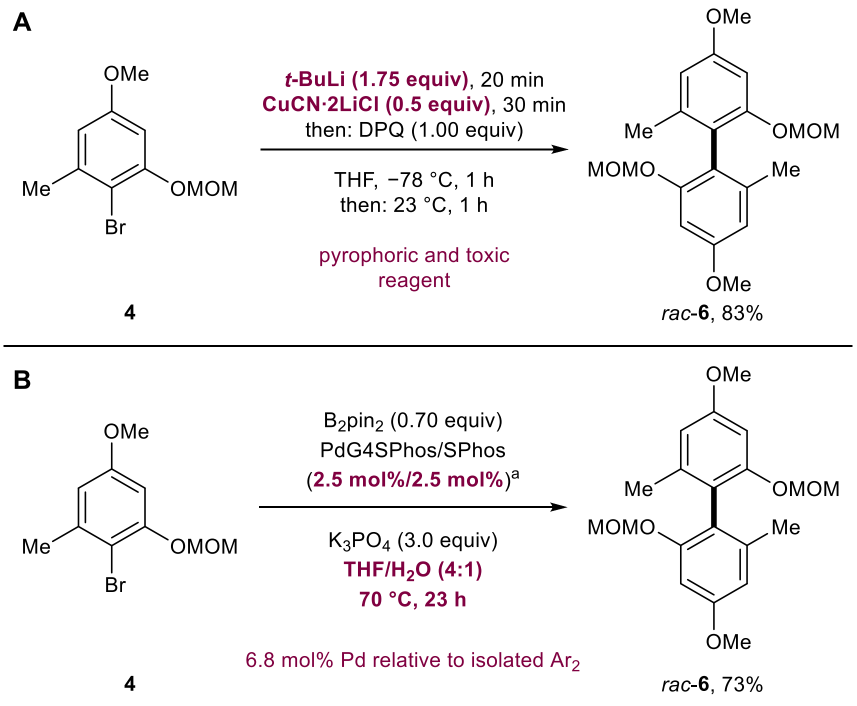

Scheme S2: Previous coupling approach.
Via the oxidation of a biaryl Lipshutz cuprate (A) or using a Miyaura borylation-Suzuki coupling (MBSC) one-pot approach (B).

We wondered whether the challenging Suzuki coupling step could be further improved with respect to temperature, solvent and especially palladium loadings. The needed aryl boronic acid pinacol ester **5** was made available via a classic borylation approach. With the boronate in hand, we systematically optimized our previous Suzuki coupling conditions. The corresponding optimization results are shown in **Table S1**.

A solvent mixture of 4:1 THF/water was confirmed to be superior compared to pure organic solvent systems like tetrahydrofuran and toluene (entries 1 – 3) (Ganardi et al., Tietze et al., 2014). Most notably, we found that the use an aqueous micellar reaction medium based on Lipshutz's TPGS-750-

M surfactant significantly facilitated the coupling reaction, enabling conversions at room temperature (entry 4) (Lipshutz et al., 2011). Addition of 10% cosolvent was employed to ensure a stable emulsion (Gabriel et al., 2017). The use of Buchwald's SPhosG4 precatalyst in combination with slight heating further improved conversions at significantly reduced palladium loadings from 3.5 to 1.0 mol% (entry 5). Scaling was performed using slightly increased amounts of aryl boronate, which could be employed as crude product. Full conversions were observed, and the desired product could be isolated in 95% yield (entry 7). Further reduction of catalyst loadings let to an incomplete conversion (entry 6).

Table S1. **Optimization of Suzuki Coupling in Water**

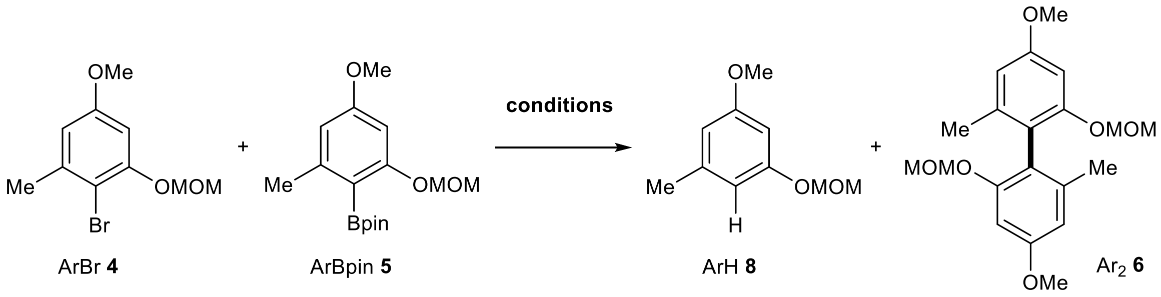

Entry	Conditions	Conversions [%][a]				Comment
		**ArBr**	**ArH**	**ArBpin**	**Ar2**	
**1**	Pd(OAc)2/ SPhos<br>(3.5/ 7.0 mol%)<br>ArBr (1.0 equiv)<br>ArBpin (1.25 equiv)<br>K3PO4 (3.0 equiv)<br>**THF**<br>70 °C, 15 h	43 <97>	32 <72>	0 <0>	25 <56>	[0.05 mmol]<br><Integral(Ar) = 225>
**2**	Pd(OAc)2/ SPhos<br>(3.5/ 7.0 mol%)<br>ArBr (1.0 equiv)<br>ArBpin (1.25 equiv)<br>K3PO4 (3.0 equiv)<br>**PhMe**<br>70 °C, 15 h	45 <101>	31 <70>	13 <29>	11 <25>	[0.05 mmol]<br><Integral(Ar) = 225>
**3**	Pd(OAc)2/ SPhos	23 <52>	28 <63>	0 <0>	49 <110>	[0.05 mmol]

	(3.5/ 7.0 mol%)<br>ArBr (1.0 equiv)<br>ArBpin (1.25 equiv)<br>K_3PO_4 (3.0 equiv)<br>**THF/H_2O (4:1)**<br>70 °C, 17 h					<Integral(Ar) = 225>
**4**	Pd(OAc)$_2$/ SPhos<br>(3.5/ 7.0 mol%)<br>ArBr (1.00 equiv)<br>ArBpin (1.25 equiv)<br>K_3PO_4 (3.0 equiv)<br>**2% TPGS-750-M**<br>**in H_2O**<br>10% THF<br>**22 °C, 2 h**	25 <57>	34 <76>	0 <0>	41 <93>	**reaction at room temperature**<br>[0.1 mmol]<br><Integral(Ar) = 225>
**5**	**PdG4SPhos/ SPhos**<br>**(1.0/ 1.0 mol%)**<br>ArBr (1.00 equiv)<br>ArBpin (1.25 equiv)<br>K_3PO_4 (3.0 equiv)<br>2% TPGS-750-M<br>in H_2O<br>10% THF<br>55 °C, 0.5 h<br><br>55 °C, 3.5 h	22 <50><br>16 <37>	11 <24><br>18 <41>	13 <29><br>0 <0>	54 <122><br>65 <147>	**precatalyst superior**<br>[0.1 mmol]<br><Integral(Ar) = 225>
**6**	PdG4SPhos/ SPhos<br>**(0.5/ 0.5 mol%)**<br>ArBr (1.00 equiv)<br>**crude ArBpin**<br>**(1.50 equiv)**<br>K_3PO_4 (3.0 equiv)<br>2% TPGS-750-M	13 <32>	37 <93>	0 <1>	50 <126><br>(61)	**ArBpin crude**<br>**(15% ArH)**<br>[5.66 mmol]<br><Integral(Ar) = 250>

	in H_2O<br>10% THF<br>50 °C, 22 h					
**7**	PdG4SPhos/ SPhos<br>**(1.0/ 1.0 mol%)**<br>ArBr (1.00 equiv)<br>**crude ArBpin**<br>**(1.50 equiv)**<br>K_3PO_4 (3.0 equiv)<br>2% TPGS-750-M<br>in H_2O<br>10% THF<br>55 °C, 16 h	0 <0>	23 <67>	0 <0>	73 <183><br>(95)	**ArBpin crude**<br>**(15% ArH)**<br>[5.66 mmol]<br>**Isolated: 1.9 g**<br><Integral(Ar) = 250>

[a] Observed rations by ^{1}H-NMR analysis of worked-up reaction samples or crude products (integral sum of all common parent aryl group signals 'Integral(Ar**)' set to 100). In chevrons ('<>') the same ratio is give with the integral sum of aryl group signals set to the total amount of** ArBr **and** ArBpin **starting material 'aryl equivalents' (e.g. Integral(Ar) = 225 for 1.00 equiv** ArBr **+ 1.25 equiv** ArBpin**). While maybe a bit unusual, this procedure allows for a more direct determination of the individual conversions of** ArBr **and** ArBpin**, which carry the same parent aryl motif. Isolated yields are given in parentheses.**

The fully optimized Suzuki coupling conditions are summarized in Fehler! Verweisquelle konnte nicht gefunden werden.. The overall yield of biaryl **6** relative to the employed aryl bromide **4** is 68% over two steps. While the yield is comparable to the previous one-pot borylation-Suzuki coupling approach (**Scheme S2**), a significant decrease in palladium loading could be achieved (1.1 mol% instead of 6.8 mol% relative to the amount of isolated product).

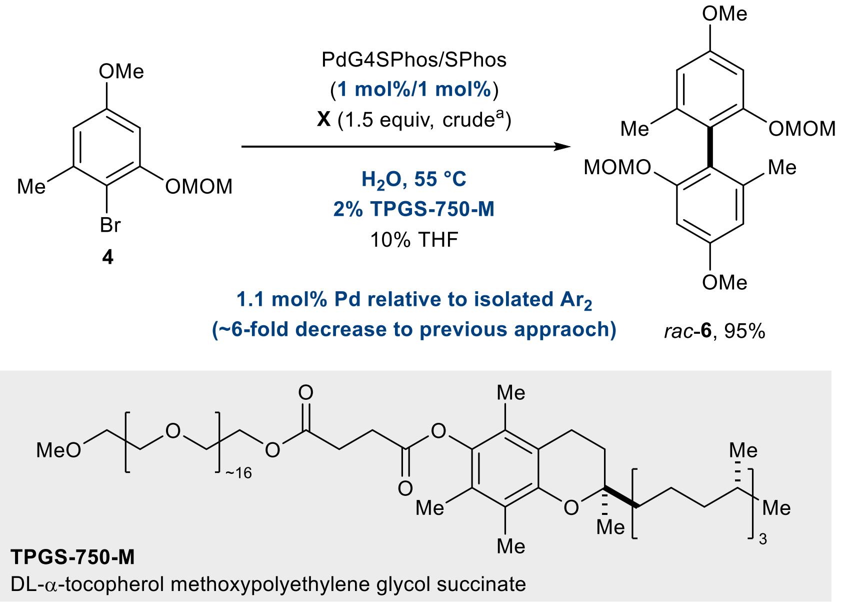

Scheme S3: Optimized Suzuki coupling with reduced Pd-loading, temperature and solvent using micellar catalysis.
[a] determined by ^{1}H-NMR, protodehalogenated aryl side product as impurity.

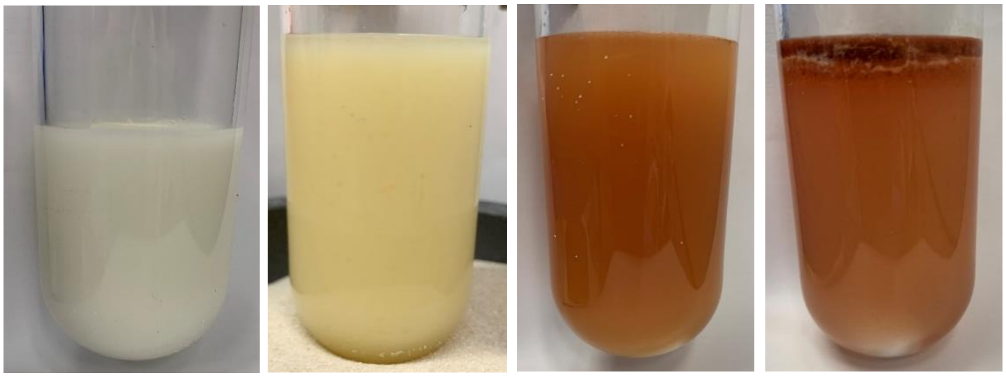

Figure S1: Reaction mixture of Suzuki coupling under aqueous micellar conditions.
From left to right: (a) starting materials and reagents as homogeneous water/TPGS-750-M emulsion (2% surfactant, 10% tetrahydrofuran cosolvent) before base addition; (b) reaction emulsion after base addition, the yellow color is indicative for precatalyst activation; (c) reaction mixture after 16 h mild heating and stirring; (d) reaction mixture after standing for a while, an organic crude product layer formed.

1.3.1 Systematic Evaluation of Preparative HPLC-Conditions

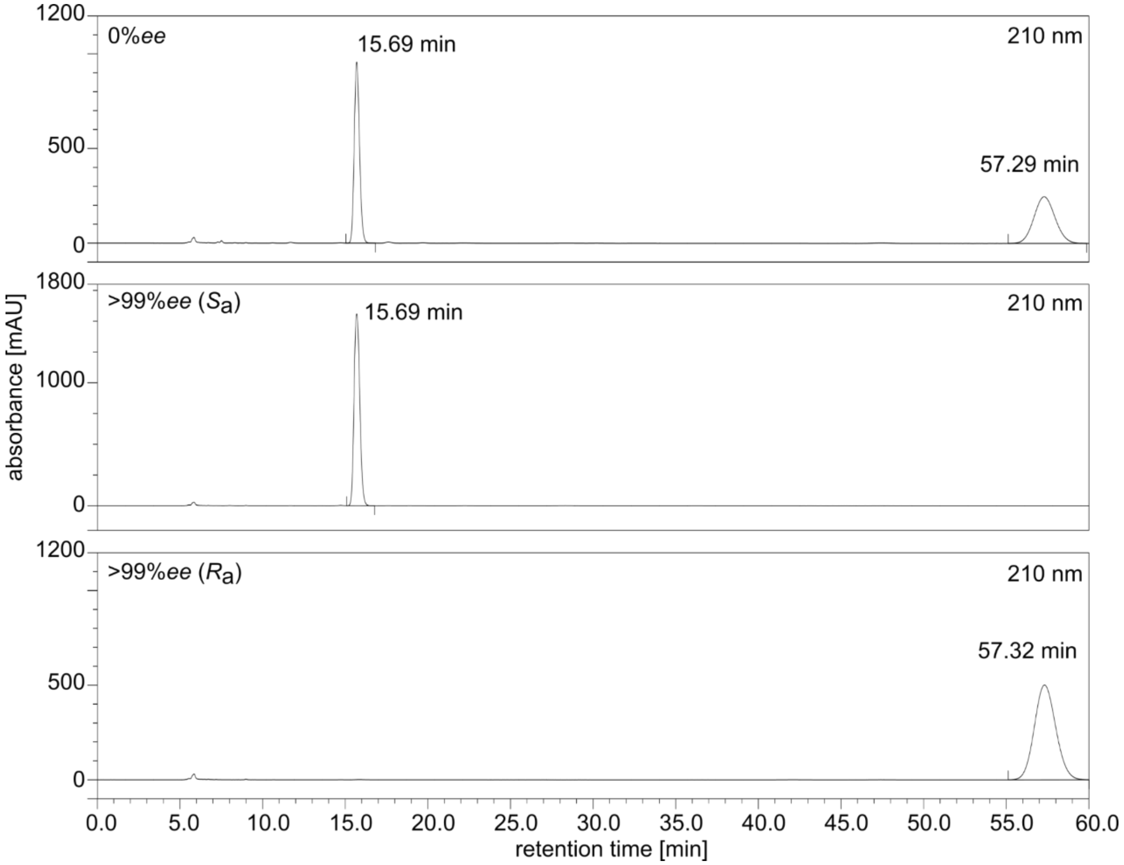

Figure S2: Comparative HPLC analysis of racemic and enantio-pure biphenol 3. Racemic mixture (top), (*S*a)-configured- (middle) and (*R*a)-configured biphenol (bottom). Analytical HPLC conditions: LuxAmylose-1 (*Phenomenex*), 250.0 × 4.6 mm, 5 µm, 25 °C, 0.5 mL min^{-1}, 210 m, 50:50 (v/v) *n*-heptane/*i*-propanol.

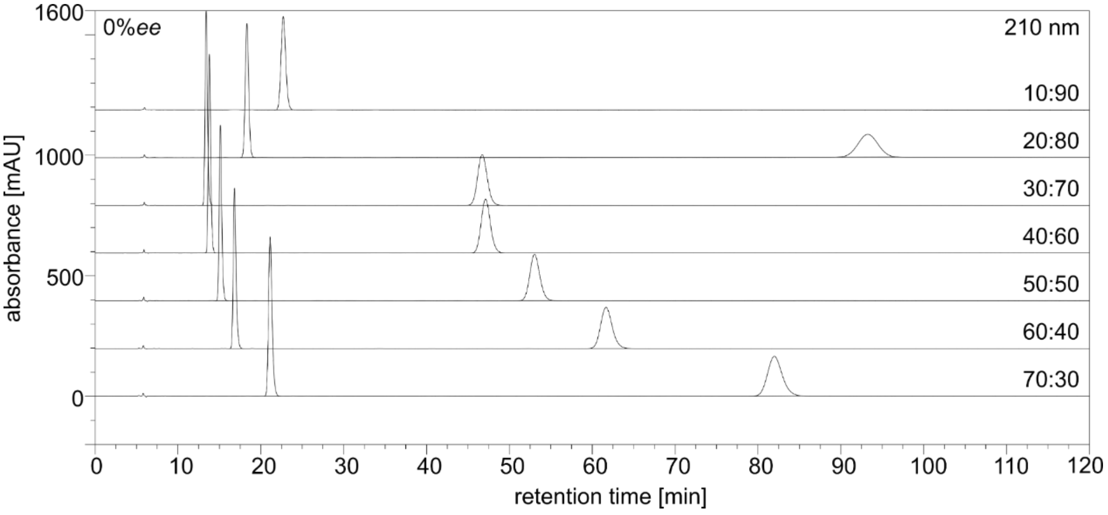

Figure S3: HPLC-separation behavior of racemic biphenol 3 on an analytical column. LuxAmylose-1 (*Phenomenex*), 250.0 × 4.6 mm, 5 µm, 25 °C, 0.5 mL min^{-1}, 210 m, *n*-heptane/*i*-propanol; retention times for (S_a)-3/(R_a)-3 at a different *n*-heptane : *i*-propanol (v/v) mixtures were 21.1 min/82.2 min (70:30), 16.8 min/61.7 min (60:40), 15.1 min/53.0 min (50:50), 13.8 min/47.1 min (40:60), 13.4 min/46.7 min (30:70), 18.2 min/93.5 min (20:80), 22.7 min/>120.0 min (10:90).

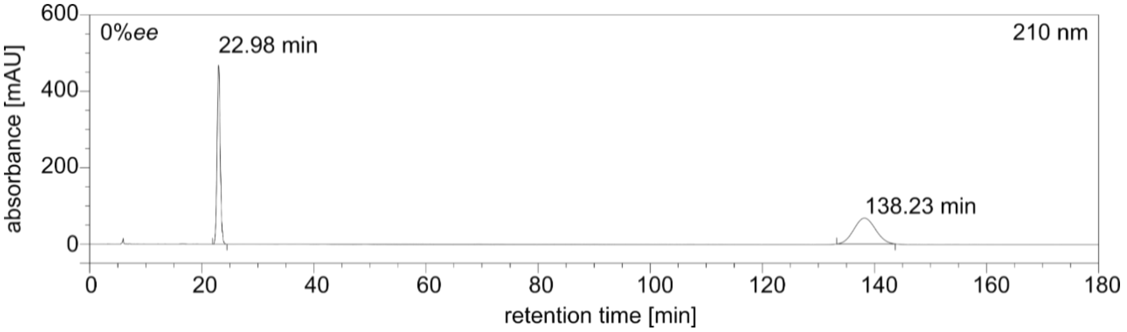

Figure S4: HPLC-separation of racemic biphenol 3 on an analytical column with 10:90 (v/v) *n*-heptane/*i*-propanol and increased runtime. LuxAmylose-1 (*Phenomenex*), 250.0 × 4.6 mm, 5 µm, 25 °C, 0.5 mL min^{-1}, 210 m, *n*-heptane/*i*-propanol.

Based on the observed retention times t_{R1}(S_a-**3**), t_{R2}(R_a-**3**) and the void time t_{R0}, retention factors $k_1 = (t_{R1} - t_{R0})/t_{R0}$ and $k_2 = (t_{R2} - t_{R0})/t_{R0}$ as well as a separation factors (chiral selectivities) $\alpha = k_2/k_1$ could be calculated for eluents of different polarities. These are listed in **Table 2**.

Table 2: Retention Factors k_1/k_2 and Separation Factors α for Eluents with Increasing Polarity.

HepH/*i*-PrOH	$t_{R1}(S_a)$ [min]	$k_1(S_a)$	$t_{R0}(R_a)$ [min]	$k_2(R_a)$	α
70 : 30	21.1	2.6	82.2	13.2	5.0
60 : 40	16.8	1.9	61.7	9.6	5.1
50 : 50	15.1	1.6	53.0	8.1	5.1
40 : 60	13.8	1.4	47.1	7.1	5.2
30 : 70	13.4	1.3	46.7	**7.1**	5.4
20 : 80	18.2	2.1	93.5	15.1	7.1
10 : 90	23.0	3.0	138.2	22.8	**7.7**

Based on analytical separation runs on LuxAmylose-1 (*Phenomenex*), 250.0 × 4.6 mm, 5 µm, 25 °C, 0.5 mL min^{-1}, 210 m (Figures Figure S, Figure S and Figure S); the void time t_{R0} was determined to be 5.8 min based on the injection peak; for an eluent mixture of 10:90 the highest separation factor was observed, at an eluent mixture of 30:70 the lowest retention factor for the later-eluting enantiomer was observed.

The chiral stationary phase (CSP) of LuxAmylose-1 (*Phenomenx*) is based on the well-known chiral selector amylose tris(3,5-dimethylphenyl)carbamate (ADMPC) in combination with a silica gel matrix. The same chiral selector is used in other commercial HPLC columns like Chiralpak AD (*Daicel*) or Chiralpak IA (*Daicel*). Good separation factors of 2 – 5 for the separation of atrop-enantiomeric analytes has been reported recently (Rizzo et al., 2022, Sechi et al., 2023). The structure of polymeric ADMPC (shown in **Figure S5**) is known do adopt a helical conformation (Yamamoto et al., 2002). Also, unusual separation behaviours as a function of the eluent composition including for example inversion of the elution order of enantiomers have been observed by others (Wang and Chen, 1999, Wang et al., 2000). These have been attributed to an alteration of the CSP's chiral cavities (stereo environment) by solvent incorporation or changes in the higher order structure of ADMPC, such as the degree of twisting of the amylose helix or differences in crystallinity, especially for higher concentration of branched alcohols, such as *i*-propanol or *t*-butanol (Wang and Wenslow, 2003).

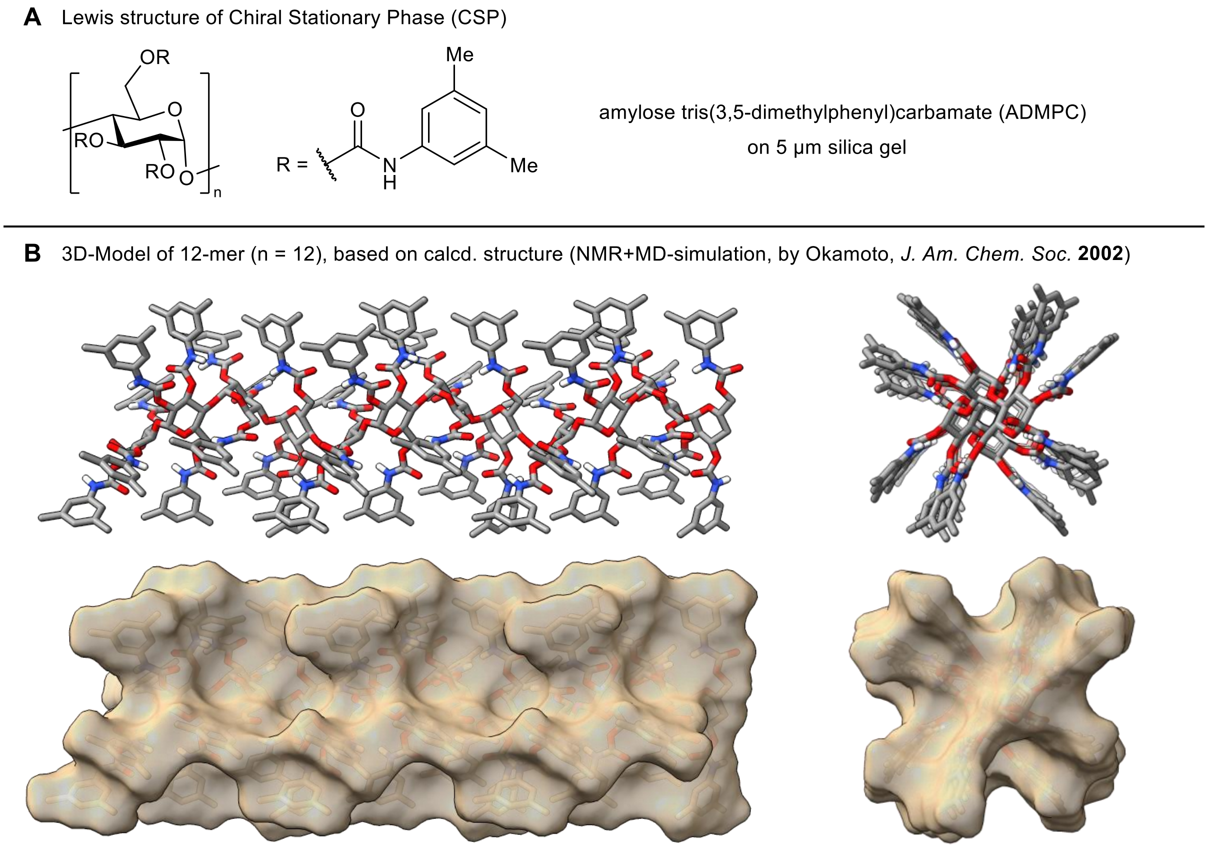

Figure S5: Structural Representations of the LuxAmylose-1 Chiral Selector ADMPC. Polymer 2D-Lewis structure (A) and helical 3D-model (B); the 3D-model was generated based on the structural data published by Ye (Ye et al., 2007), which in turn has been calculated by Okamato and coworkers via experimental NMR-constraints in combination with MD-simulations (Yamamoto et al., 2002). The visualization was generated based on the published pdb-file using ChimeraX (Pettersen et al., 2021) and is depicted from the side (B, left) or top (B, right) as stick model without (B, top) and with surface (solvent-excluded surfaces, 2.0 Å probe radius) (B, bottom).

1.4 Synthetic Procedures

In the following, the synthetic procedures used in this work are outlined. The syntheses of monomeric chalcones as well as the racemic synthesis of bichalcones has previously been published (Klischan et al., 2023). Our enantioselective bichalcone synthesis commenced with methoxymethyl (MOM)-protected 2-bromophenol **4**, which was synthesized following a scalable and column-free procedure starting from commercially available orcinol (Greb et al., 2023).

1.4.1 Synthesis of 2-(4-methoxy-2-(methoxymethoxy)-6-methylphenyl)-4,4,5,5-tetramethyl-1,3,2-dioxaborolane (5)

A dry 50-mL Schlenk-tube was equipped with a magnetic stirring bar and septum and charged with 2-bromo-5-methoxy-1-(methoxymethoxy)-3-methylbenzene (**4**, 522 mg, 2.0 mmol, 1.0 equiv) (Greb et al., 2023) under a nitrogen atmosphere. The starting material was stirred at room temperature and

degassed by three cycles of evacuation and nitrogen backflushing. Subsequently, 20 mL dry and degassed tetrahydrofuran were added, and the resulting solution was cooled to −78 °C. A solution of *n*-butyllithium (2.18 M in hexane, 1.0 mL, 2.2 mmol, 1.1 equiv) was added dropwise and stirring was continued for 30 min. Dry trimethyl borate (340 µL, 3.0 mmol, 1.5 equiv) was added dropwise and stirring was continued for 30 min, after which the solution was allowed to warm to room temperature for 30 min. Subsequently, pinacol (473 mg, 4.0 mmol, 2.0 equiv) was added in a single portion. The resulting solution was stirred for 20 h at room temperature. After cooling to 0 °C, the solution was poured into 40 mL of an ice-cold and stirred, saturated aqueous solution of ammonium chloride. If needed, the mixture was carefully adjusted to pH 5 – 7 using 1 M aqueous hydrogen chloride. The mixture was transferred into a separation funnel, rinsing the used glass ware with water and ethyl acetate. After adding 50 mL ethyl acetate, the resulting two-phase system was mixed, the phases were separated, and the aqueous phase was extracted twice using 50 mL ethyl acetate. The combined organic phases were washed with brine, dried over magnesium sulphate, and concentrated in vacuo. The resulting crude product, a clear yellow oil, was analysed by ^{1}H-NMR and purified by column chromatography (petroleum ether:ethyl acetate, 90:10 → 80:20 v/v) yielding the title compound as colourless oil in a yield of 491 mg (1.6 mmol, 80%).

In a separate experiment the reaction was scaled including slight adjustments of the reagent equivalents, but otherwise identical conditions as described above. In a 100-mL Schlenk-tube 2-bromo-5-methoxy-1-(methoxymethoxy)-3-methylbenzene (2.6 mg, 10.0 mmol, 1.00 equiv) in 50 mL dry tetrahydrofuran was reacted with *n*-butyllithium (2.30 M in hexane, 4.4 mL, 10.2 mmol, 1.02 equiv), dry trimethyl borate (1.18 mL, 10.5 mmol, 1.05 equiv) and pinacol (1.3 g, 11.0 mmol, 1.10 equiv). After 16 h reaction time, quenching, and work up, the crude product was determined to be 85% pure (by ^{1}H-NMR, 15% protodehalogenated side product) and could be used in the subsequent coupling step without further purification.

The analytical data was in accordance with previously published results (Ganardi et al.).

TLC (petroleum ether:ethyl acetate, 90:10 v/v): R_f = 0.20.

: δ [ppm] = 1.36 (s, 12H; 6′-H, 7′-H, 8′-H, 9′-H), 2.34 (s, 3H; 10-H), 3.46 (s, 3H; 8-H), 3.76 (s, 3H; 9-H), 5.11 (s, 2H; 7-H); 6.36 (d, *J* = 2.1 Hz; 5-H), 6.41 (d, *J* = 2.1 Hz; 3-H).

^{13}C NMR (151 MHz, $CDCl_3$): δ [ppm] = 22.3 (C-10), 24.9 (C-6′, C-7′, C-8′, C-9′), 55.3 (C-9), 56.1 (C-8), 83.6 (C-4′, C-5′), 94.7 (C-7), 98.3 (C-3), 108.6 (C-5), 112.5 (C-1, very weak), 144.5 (C-6), 161.8 (C-4), 162.0 (C-2).

^{11}B NMR (96 MHz, $CDCl_3$): δ [ppm] = 31.3 (B-2′).

IR (ATR film): ṽ [cm^{-1}] = 2977, 1603, 1371, 1337, 1299, 1141, 1052, 1035, 926, 857, 834, 670.

HR-MS (ESI): m/z calcd. for $[C_{16}H_{26}BO_5]^+$ ([M + H^+]) = 309.1868, found: 309.1876.

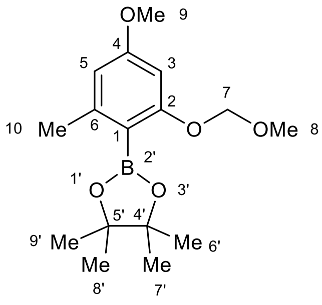

5

1.4.2 Synthesis of 4,4'-dimethoxy-2,2'-bis(methoxymethoxy)-6,6'-dimethyl-1,1'-biphenyl (6)

A 100 mL-Schlenk tube or flask was charged with 2-bromo-5-methoxy-1-(methoxymethoxy)-3-methylbenzene (**4**, 1.48 g, 5.66 mmol, 1.00 equiv) (Greb et al., 2023), crude 2-(4-methoxy-2-(methoxymethoxy)-6-methylphenyl)-4,4,5,5-tetramethyl-1,3,2-dioxaborolane (**5**, 85% purity (see above), 3.08 g, 8.49 mmol, 1.50 equiv), PdSPhosG4 precatalyst (45 mg, 0.57 mmol, 1.0 mol%) and SPhos (23 mg, 0.57 mmol, 1.0 mol%) under a nitrogen atmosphere. The starting materials were stirred at room temperature and degassed by three cycles of evacuation and nitrogen backflushing. As it simplified the set-up operation, TPGS-750-M (1.00 g, 2%(w/w) relative to aqueous part of the final solvent mix) was degassed analogously in a separate 10 mL-Schlenk-tube and dissolved in 5.5 mL dry degassed tetrahydrofuran. Subsequently, the organic surfactant solution was added to the starting materials and reagents forming a yellowish clear solution. While stirring, 30 mL of degassed water were added to the organic solution resulting in the formation of a white homogeneous emulsion (see **Figure S**). A separate 50-mL Schlenk-flask was charged with potassium phosphate (3.60 g, 16.98 mmol, 3.00 equiv) under a nitrogen atmosphere, which was dissolved in 20 mL degassed water. The aqueous base solution was added to the emulsion, which turned yellow indicting precatalyst activation and start of the reaction (see **Figure S**). Subsequently, the reaction mixture was subjected to mild heating at 55 °C under vigorously stirring for 16 h. Upon full conversion, as indicated by TLC, stirring was stopped and the reaction mixture was warmed to room temperature. While standing, the emulsion separated, and an orange organic crude product phase formed on the surface of the aqueous layer (see **Figure S**). The reaction mixture was diluted with ethyl acetate and transferred into a separation funnel. The reaction vessel was rinsed properly with water and ethyl acetate. After mixing of the resulting two-phase system, the layers were separated, and the aqueous phase was extracted three times with 30 mL ethyl acetate. The combined organic layers were washed with brine, dried over magnesium sulphate and concentrated in vacuo. The resulting crude product was analysed by ^{1}H-NMR and purified by column chromatography (petroleum ether:ethyl acetate, 80:20 v/v) yielding the racemic title compound as slightly orange oil in a yield of 1.94 g (5.35 mmol, 95%).

The analytical data was in accordance with previously published results (Ganardi et al., Greb et al., 2023):

TLC (petroleum ether:ethyl acetate, 80:20 v/v): R_f = 0.27.

: δ [ppm] = 6.64 (d, J = 2.5 Hz, 2H; 3-H, 3′-H), 6.52 (d, J = 2.5 Hz, 2H; 5-H, 5′-H), 5.01 (d, J = 6.6 Hz, 2H; 7b-H, 7b′-H), 4.97 (d, J = 6.6 Hz, 2H; 7a-H, 7a′-H), 3.82 (s, 6H; 9-H, 9′-H), 3.30 (s, 6H; 8-H, 8′-H), 1.95 (s, 6H; 10-H, 10′-H).

^{13}C NMR (151 MHz, $CDCl_3$): δ [ppm] = 159.5 (C-4, C-4′). 156.0 (C-2, C-2′), 139.5 (C-6, C-6′), 119.8 (C-1, C-1′), 108.5 (C-5, C-5′), 99.6 (C-3, C-3′), 94.9 (C-7, C-7′), 55.8 (C-8, C-8′), 55.3 (C-9, C-9′), 20.3 (C-10, C-10′).

IR (ATR film): ṽ [cm^{-1}] = 2952, 1602, 1466, 1310, 1209, 1145, 1041, 994, 923, 833, 635, 516.

MS (ESI): m/z = 385.2 ([M + Na^+]).

HR-MS (ESI): m/z calcd. for $[C_{20}H_{26}O_6Na]^+$ ([M + Na^+]) = 385.1622, found: 385.1618.

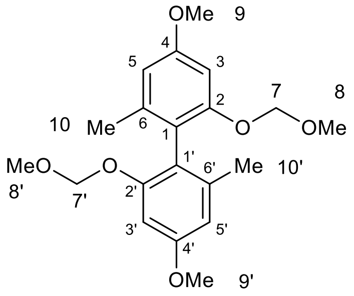

6

1.4.3 Synthesis of 4,4'-dimethoxy-6,6'-dimethyl-[1,1'-biphenyl]-2,2'-diol (3)

A 100-mL round-bottom flask equipped with a magnetic string bar as well as a reflux condenser and charged with 4,4'-dimethoxy-2,2'-bis(methoxymethoxy)-6,6'-dimethyl-1,1'-biphenyl (**6**, 1.2 g, 3.31 mmol, 1.0 equiv) in 33 mL degassed methanol under a nitrogen atmosphere. Aqueous hydrochloric acid (4 M, 0.83 mL, 3.31 mmol, 1.0 equiv) was added and the resulting mixture was heated at 70 °C for 1 – 2 h. Careful TLC monitoring was performed to avoid methyl ether-cleavage. Upon full conversion, as indicated by TLC, the reaction was cooled to room temperature neutralized using a saturated aqueous solution of sodium bicarbonate. The resulting mixture was transferred into a separation funnel and diluted with 30 mL water and 30 mL ethyl acetate, rinsing the used glass ware properly. After mixing, the phases were separated, and the aqueous layer was extracted three times with 30 mL ethyl acetate. The combined organic layers were washed twice with brine and dried over magnesium sulphate. Removal of the solvent at reduced pressure yielded the racemic title compound as slightly yellow crystalline solid in a yield of 899 mg (3.27 mmol, 99%), which could be used without further purification.

The analytical data was in accordance with previously published results (Ganardi et al., Greb et al., 2023):

TLC (petroleum ether:ethyl acetate, 80:20 v/v): R_f = 0.12.

Melting point: 148 – 150 °C (*rac*).

: δ [ppm] = 6.48 (d, *J* = 2.5 Hz, 2H; 5-H, 5′-H), 6.46 (d, *J* = 2.5 Hz, 2H; 3-H, 3′-H), 4.86 (s, 2H; 2-OH, 2′-OH), 3.80 (s, 6H; 7-H, 7′-H), 1.97 (bs, 6H; 8-H, 8′ H).

^{13}C NMR (151 MHz, $CDCl_3$): δ [ppm] = 161.2 (C-4, C-4′), 155.5 (C-2, C-2′), 140.5 (C-6, C-6′), 111.4 (C-1, C-1′), 109.0 (C-5, C-5′), 98.5 (C-3, C-3′), 55.4 (C-7, C-7′), 19.9 (C-8, C-8′).

IR (ATR film): ṽ [cm^{-1}] = 3505, 3360, 2960, 1612, 1575, 1446, 1310, 1197, 1141, 1069, 1038, 934, 840, 824, 608, 497.

MS (ESI): m/z = 275.0 ([M + H^+]).

HR-MS (ESI): m/z calcd. for $[C_{16}H_{17}O_4]^-$ ([M – H^+]) = 273.1132, found: 273.1128.

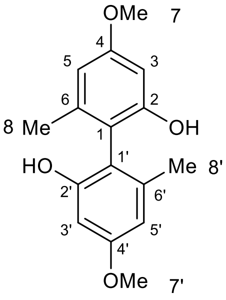

3

1.4.4 Resolution of 4,4'-dimethoxy-6,6'-dimethyl-[1,1'-biphenyl]-2,2'-diol (3)

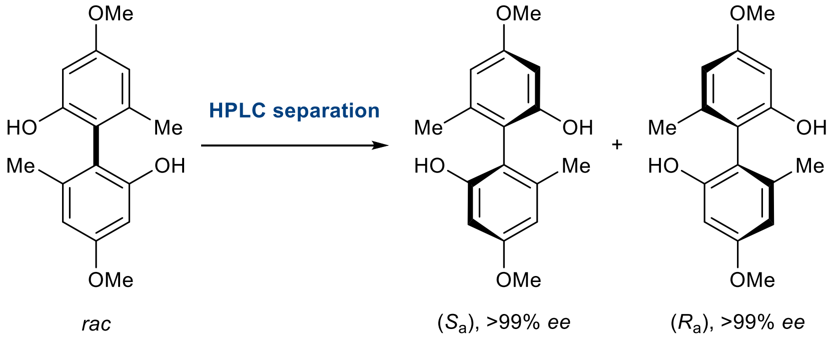

A saturated solution of racemic 4,4'-dimethoxy-6,6'-dimethyl-[1,1'-biphenyl]-2,2'-diol (**3**, 530 mg, 1.94 mmol) in 10:90 (v/v) *n*-heptane/*i*-propanol was prepared by stirring and sonication. After standing at room temperature for 1 – 2 h or overnight (to avoid later precipitate formation, potential temperature/solubility increase due to sonication), the solution was passed through a syringe filter. Enantiomer separation was performed using a Lux® Amylose-1, 5 µm, 250.0 × 21.2 mm, AXIA™ (*Phenomenex*) preparative HPLC column with a suitable HPLC pump system and a diode array (detection at 210 nm). Depending on the polarity of the eluent used, flow rates between 8 and 12 mL/min were used to, conservatively, not exceed maximal backpressures of 100 – 110 bar

(250 bar suggested by manufacturer). After column equilibration with 10:90 (v/v) *n*-heptane/*i*-propanol, the prepared solution of racemic biphenol was injected using an appropriate sample loop. Within the loop, the sample solution has been 'sandwiched' (15% eluent, 70% sample, 15% eluent) to avoid compound loss during loading. Fractions were collected based on the live diode array signal. Due to the high selectivity factor α of 7.7, maximal loading/overloading the column did not at all impede a clean separation. Once roughly half of the earlier enantiomer had been eluted (roughly 20 – 25 min at 8 – 10 mL/min), the eluent was changed to 30:70 (v/v) *n*-heptane/*i*-propanol (lowest k_2 observed) and flowrates were increased, staying within the pressure limits, to shorten overall runtimes. After complete elution, the column was equilibrated to storage conditions and the collected fractions were concentrated at reduced pressure yielding 262 mg (0.946 mmol, 49%, >99%*ee* S_a) and 257 mg (0.937 mmol, 49%, >99%*ee* R_a) respectively.

The stereochemistry-specific analytical data was in accordance with previously published results (Ganardi et al., Greb et al., 2023):

Melting point: 148 – 150 °C (*rac*).

172 – 174 °C (S_a, >99%*ee*).

Optical rotation: $[\alpha]^{25}_D = -31.9°$ (c = 1.04, $CHCl_3$, S_a, >99%*ee*).

$[\alpha]^{25}_D = +32.6°$ (c = 1.22, $CHCl_3$, R_a, >99%*ee*).

HPLC: Lux® Amylose-1 (*Phenomenex*), 250 × 4.6 mm, 25 °C, 0.5 mL min^{-1}, 210 nm, *n*-heptane:*i*-propanol 50:50 (v/v): $t_R(S_a)$ = 15.1 min, $t_R(R_a)$ = 53.0 min.

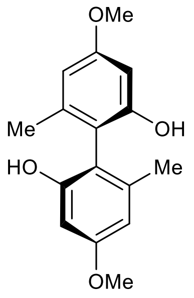

(−)-(S_a)-**3**

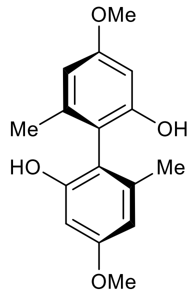

(+)-(R_a)-**3**

1.4.5 Synthesis of 1,1'-(2,2'-dihydroxy-4,4'-dimethoxy-6,6'-dimethyl-[1,1'-biphenyl]-3,3'-diyl)bis(ethan-1-one) (7)

Following our literature known procedure (Greb et al., 2023) a 50 mL Schlenk-vial equipped with stir bar was charged with biphenol **3** (274 mg, 1.0 equiv, 1.00 mmol, >99%*ee* S_a) and anhydr. chlorobenzene (10 mL, 0.1 M). The solution was stirred at 0 °C, acetyl chloride (157 µL, 2.2 equiv, 2.20 mmol) added dropwise and then the mixture stirred at room temperature for 30 minutes. $TiCl_4$ (658 µL, 6.0 equiv, 6.00 mmol) was added dropwise and the reaction mixture stirred at 70 °C for 4 h. The reaction mixture was transferred into a 250 mL Erlenmeyer beaker equipped with stir bar with

K_2HPO_4/KH_2PO_4-buffer (KPi-buffer, 1 M, 100 mL, pH 7) at 0 °C. The pH was then adjusted to pH 4 by the addition of 1 M HCl-solution. The resulting white suspension was then stirred for 15 mins, sonicated for 15 mins, and then stirred for 30 mins. The mixture was filtered over a pad of celite using a wide Buchner-type funnel. The filter cake was washed with CH_2Cl_2 (100 mL) water, then with CH_2Cl_2 (800 mL). The filter cake was then transferred into an Erlenmeyer flask and stirred with CH_2Cl_2 overnight. The suspension was then again filtered over celite. The filtrates were combined, and the aqueous phase extracted with CH_2Cl_2 (4 × 250 mL). The combined aqueous phases were washed with sat. aq. NaCl solution, dried over $MgSO_4$ and the solvent removed in vacuo. The product was isolated by column chromatography (CH_2Cl_2:PhMe 1:9 v/v) and obtained as pale yellow solids in a yield of 297 mg (0.829 mmol, 83%, >99%*ee*, S_a).

In a repeat experiment using biphenol **3** (343 mg, 1.0 equiv, 1.25 mmol, >99%*ee*, R_a) the product was isolated as pale yellow solids in a yield of 271 mg (0.756 mmol, 76%, >99%*ee* R_a).

TLC (petroleum ether:ethyl acetate, 7:3 v/v): R_f = 0.32.

Melting point: 233 – 235 °C (*rac*) (233 – 236 °C) (Greb et al., 2023).

230 – 232 °C (S_a, >99%*ee*) (209 – 210 °C) (Greb et al., 2023).

231 – 234 °C (R_a, >99%*ee*).

: δ [ppm] = 2.06 (s, 3H, Me), 2.67 (s, 3H, CO*Me*), 3.93 (s, 3H, OMe), 6.38 (s, 1H, H-5), 13.68 (s, 1H, OH).

^{13}C NMR (151 MHz, $CDCl_3$): δ [ppm] = 20.97 (Me), 33.66 (CO*Me*), 55.58 (OMe), 102.97 (C-5), 109.41 (C-3), 117.38 (C-1), 147.54 (C-6), 160.79 (C-4), 162.40 (C-2), 204.77 (*CO*Me).

IR (ATR film): ṽ [cm^{-1}] = 1599, 1360, 1283, 1202, 1119, 9693, 865, 834, 655, 573, 534.

HR-MS (ESI): m/z calcd. for $[C_{20}H_{23}O_6]^+$ ([M + H^+]) = 359.1489, found: 359.1492.

Optical rotation: $[\alpha]^{25}_D$ = +51.2° (±0.2°, duplicate) (*c* = 1.02, $CHCl_3$, S_a, >99%*ee*) (+43.1) (Greb et al., 2023).

HPLC: Lux® Amylose-1 (*Phenomenex*) 250 × 4.6 mm, 25 °C, 0.5 mL min^{-1}, 274 nm, *n*-heptane:*i*-propanol 50:50 (v/v): $t_R(S_a)$ = 7.4 min, $t_R(R_a)$ = 11.0 min.

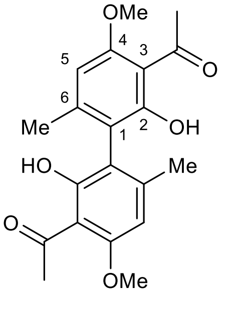

7

1.4.6 (2*E*,2'*E*)-1,1'-(2,2'-dihydroxy-4,4'-dimethoxy-6,6'-dimethyl-[1,1'-biphenyl]-3,3'-diyl)bis(3-phenylprop-2-en-1-one) (2ab)

A vial equipped with a stir bar was charged with acetophenone **7** (100 mg, 0.279 mmol, 1.0 equiv, >99%*ee* S_a) and ethanol (1.0 mL, 0.28 M) and an aqueous solution of KOH (1.12 mL, 3.35 mmol, 12.0 equiv). Once fully dissolved, benzaldehyde (67.7 µL, 0.670 mmol, 2.4 equiv) were added at once. The mixture was stirred for 4 h at 22 °C after which aq. HCl-solution (1 M, 4 mL) was added. The forming solids were filtered off and washed with MeOH:H_2O (8:2, 10 mL). The solids were then dissolved into a round bottom flask using CH_2Cl_2 and solvents removed in vacuo. The product was isolated by column chromatography (100% CH_2Cl_2). The isolated product was suspended in *n*-pentane (2 × 5 mL) and sonicated to remove traces of CH_2Cl_2. The solvent was then removed in vacuo. The product was isolated as orange amorphous solids in a yield of 81.0 mg (0.152 mmol, 54%, >99%*ee* S_a).

In a repeat experiment starting from (100 mg, 0.279 mmol, 1.0 equiv, >99%*ee* R_a) the product was isolated as orange amorphous solids in a yield of 91.1 mg (0.170 mmol, 61%, >99%*ee* R_a).

TLC (100% CH_2Cl_2): R_f = 0.36 (yellow spot)

Melting point: 213 – 215 °C (*rac*)

190 – 192 °C (S_a)

191 – 192 °C (R_a)

: δ [ppm] = 2.12 (s, 6H, Me), 4.00 (s, 6H, OMe), 6.44 (s, 2H, H-5), 7.37 – 7.45 (m, 6H, H-3''+H-4''), 7.59 – 7.65 (m, 4H, H-2''), 7.80 (d, *J* = 15.6 Hz, 2H, H-3'), 7.94 (d, *J* = 15.6 Hz, 2H, H-2'), 13.71 (s, 2H, OH).

^{13}C NMR (151 MHz, $CDCl_3$): δ [ppm] = 21.06 (Me), 55.96 (OMe), 103.50 (C-5), 110.07 (C-3), 117.82 (C-1), 128.16 (C-2'), 128.56 (C-2''), 129.04 (C-3''), 130.24 (C-4''), 135.76 (C-1''), 142.55 (C-3'), 147.57 (C-6), 160.38 (C-4), 162.89 (C-2), 194.30 (C-1').

IR (ATR film): $\tilde{\nu}$ [cm^{-1}] = 3104, 3026, 2970, 2942, 2250, 1628, 1609, 1564, 1448, 1388, 1361, 1329, 1272, 1214, 1179, 1115, 1073, 1038, 976, 948, 907, 869, 817, 789, 758, 725, 688, 647, 565, 534, 494.

HR-MS (ESI): m/z calcd. for $[C_{34}H_{31}O_6]^+$ ([M + H^+]) = 535.2115, found: 535.2122.

elemental analysis (calcd., found for $C_{34}H_{30}O_6$): C (76.39, 76.31), H (5.66, 5.59). (*rac*)

elemental analysis (calcd., found for $C_{34}H_{30}O_6$): C (76.39, 76.13), H (5.66, 5.62). (S_a)

elemental analysis (calcd., found for $C_{34}H_{30}O_6$): C (76.39, 75.91), H (5.66, 5.72). (R_a)

Chiral HPLC: >99% ee (S_a)

>99% ee (R_a)

Purity: >99% (normal phase HPLC), >99% (reverse phase HPLC) (S_a)

>99% (normal phase HPLC), >99% (reverse phase HPLC) (R_a)

96% (reverse phase HPLC), (elemental Analysis) (*rac*)

Optical rotation: $[\alpha]^{20}_{D}$ = +128.7° (±0.1°, duplicate) (c = 0.964, $CHCl_3$, S_a, >99%*ee*)

HPLC: Lux® Amylose-1 (*Phenomenex*) 2504.6 mm, 25 °C, 0.5 mL min^{-1}, 331 nm, *n*-heptane:*i*-propanol 50:50 (v/v): $t_R(S_a)$ = 14.7 min, $t_R(R_a)$ = 24.2 min.

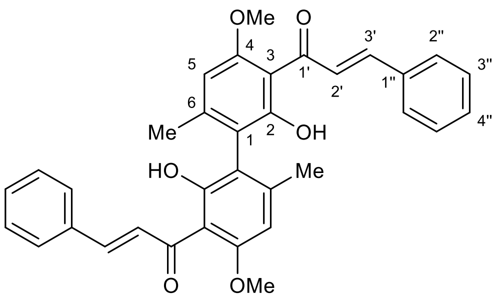

2ab

2 HPLC Chromatograms

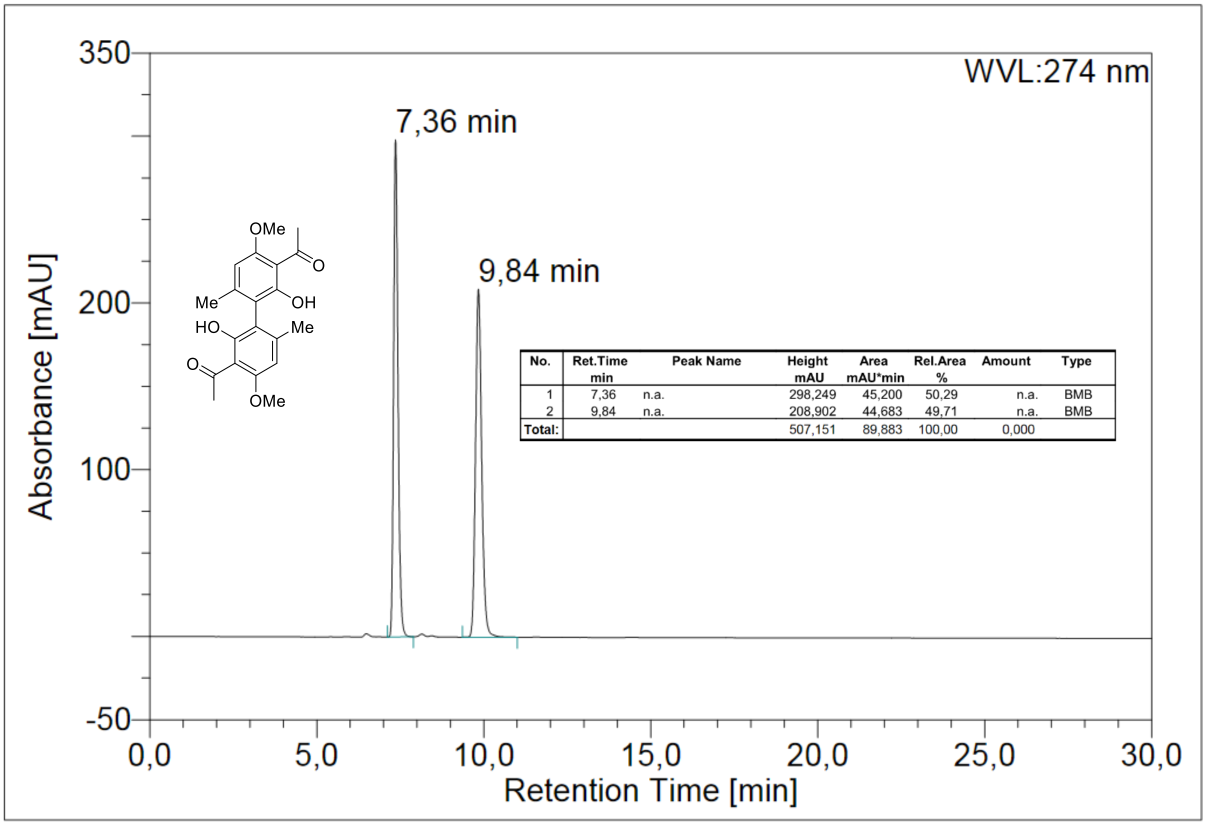

Figure S6: HPLC Chromatogram of biacetophenone 7 (*rac*) Lux® Amylose-1 (*Phenomenex*) 250 × 4.6 mm, 25 °C, 0.5 mL min^{-1}, 274 nm, *n*-heptane:*i*-propanol 50:50 (v/v): $t_R(S_a)$ = 7.4 min, $t_R(R_a)$ = 11.0 min.

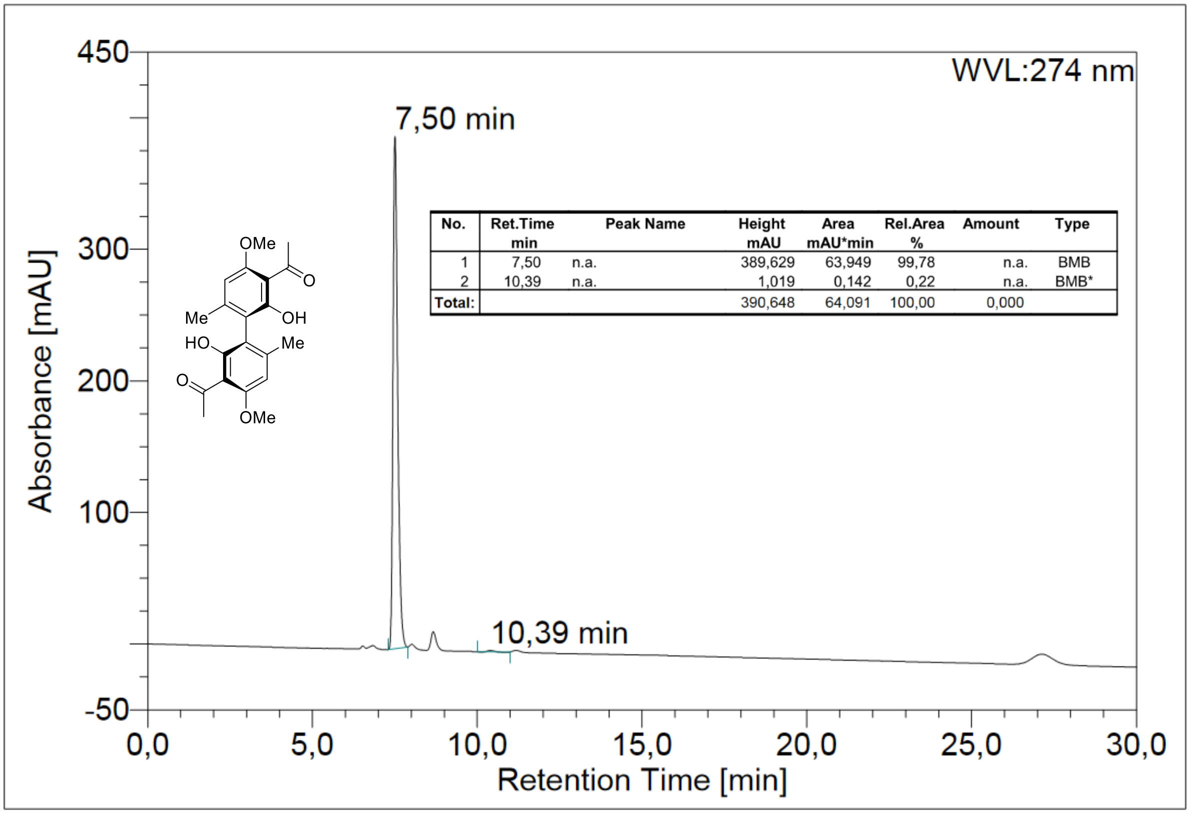

Figure S7: HPLC Chromatogram of biacetophenone 7 >99%*ee* (*S*a). Lux® Amylose-1 (*Phenomenex*) 250 × 4.6 mm, 25 °C, 0.5 mL min^{-1}, 274 nm, *n*-heptane:*i*-propanol 50:50 (v/v): t_R(S_a) = 7.4 min, t_R(R_a) = 11.0 min.

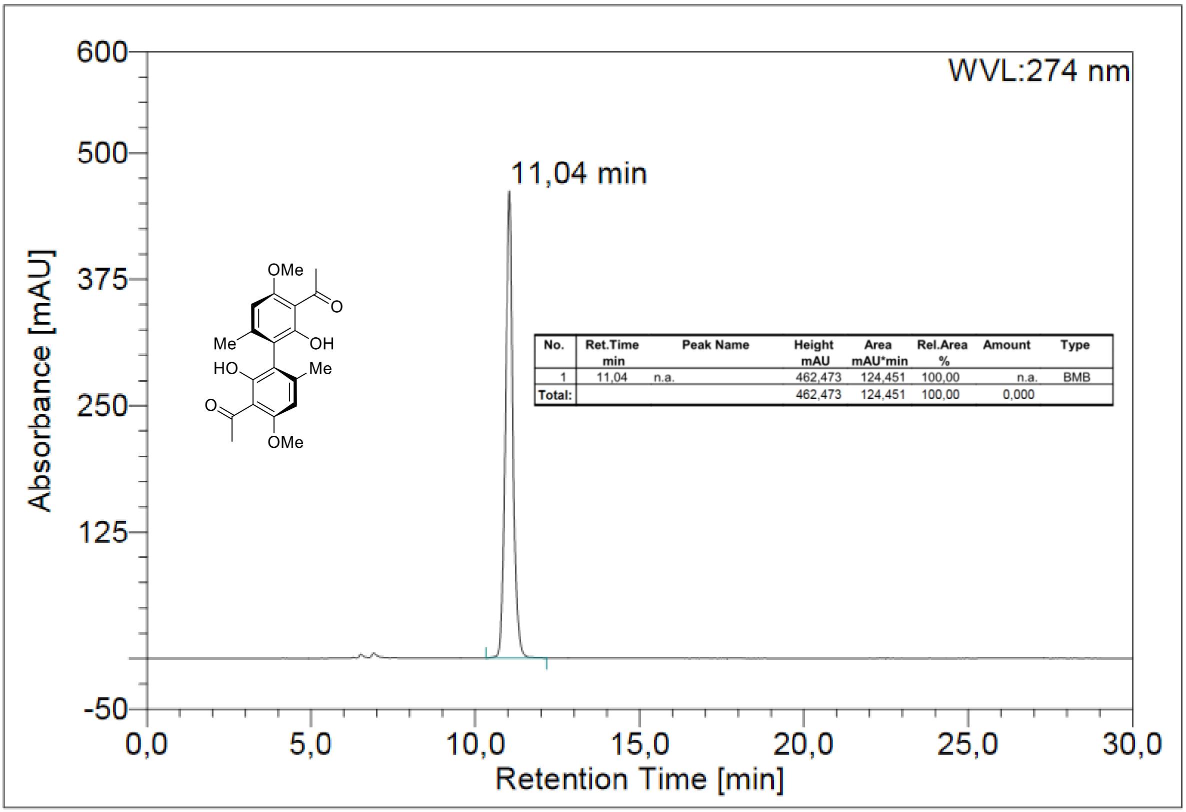

Figure S8: HPLC Chromatogram of biacetophenone 7 >99%*ee* (*R*a). Lux® Amylose-1 (*Phenomenex*) 250 × 4.6 mm, 25 °C, 0.5 mL min^{-1}, 274 nm, *n*-heptane:*i*-propanol 50:50 (v/v): t_R(S_a) = 7.4 min, t_R(R_a) = 11.0 min.

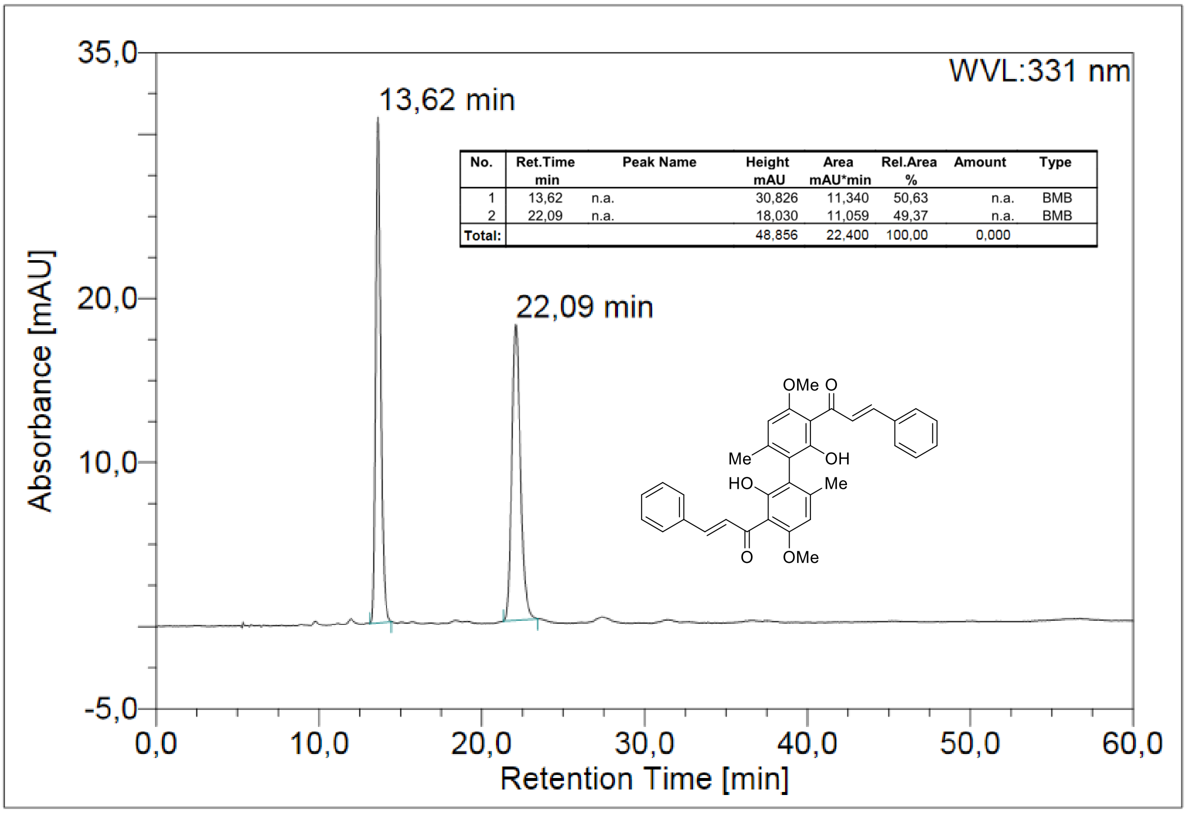

Figure S9: HPLC Chromatogram of bichalcone 2ab (*rac*). Lux® Amylose-1 (*Phenomenex*) 2504.6 mm, 25 °C, 0.5 mL min^{-1}, 331 nm, *n*-heptane:*i*-propanol 50:50 (v/v): t_R(S_a) = 14.7 min, t_R(R_a) = 24.2 min.

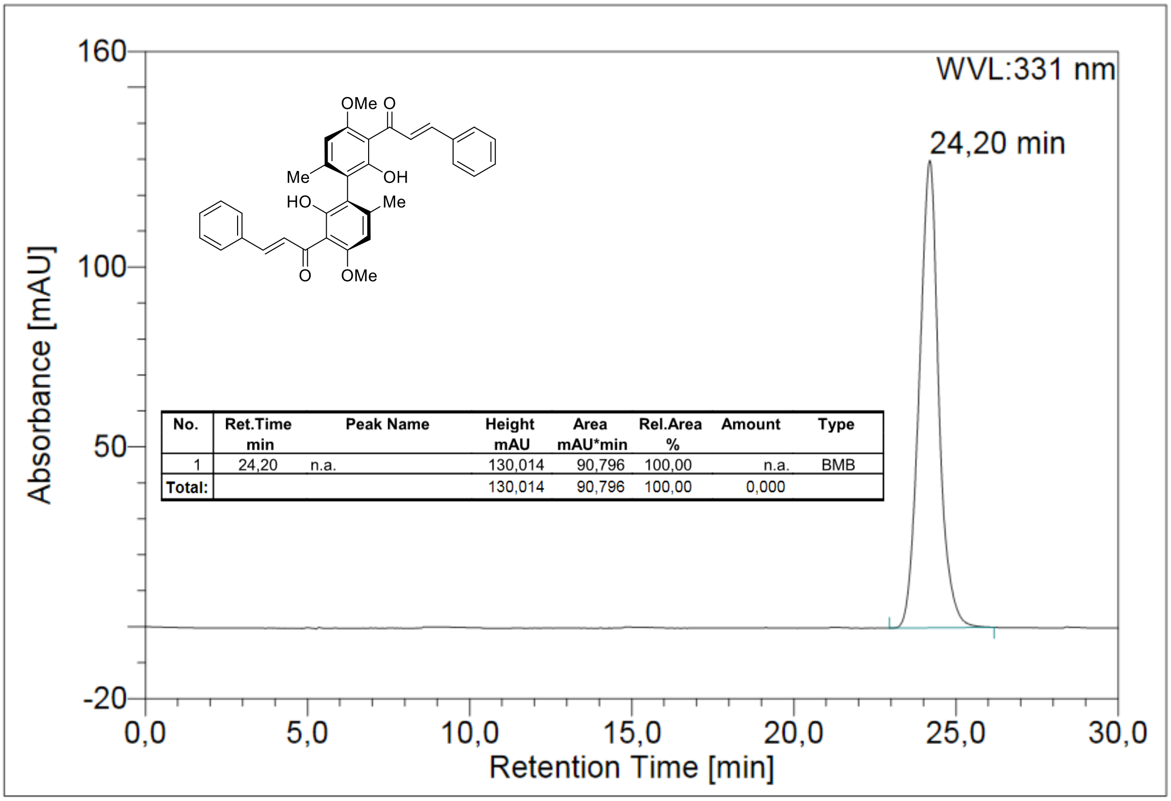

Figure S10: HPLC Chromatogram of bichalcone 2ab >99%*ee* (*R*a). Lux® Amylose-1 (*Phenomenex*) 2504.6 mm, 25 °C, 0.5 mL min^{-1}, 331 nm, *n*-heptane:*i*-propanol 50:50 (v/v): $t_R(S_a)$ = 14.7 min, $t_R(R_a)$ = 24.2 min.

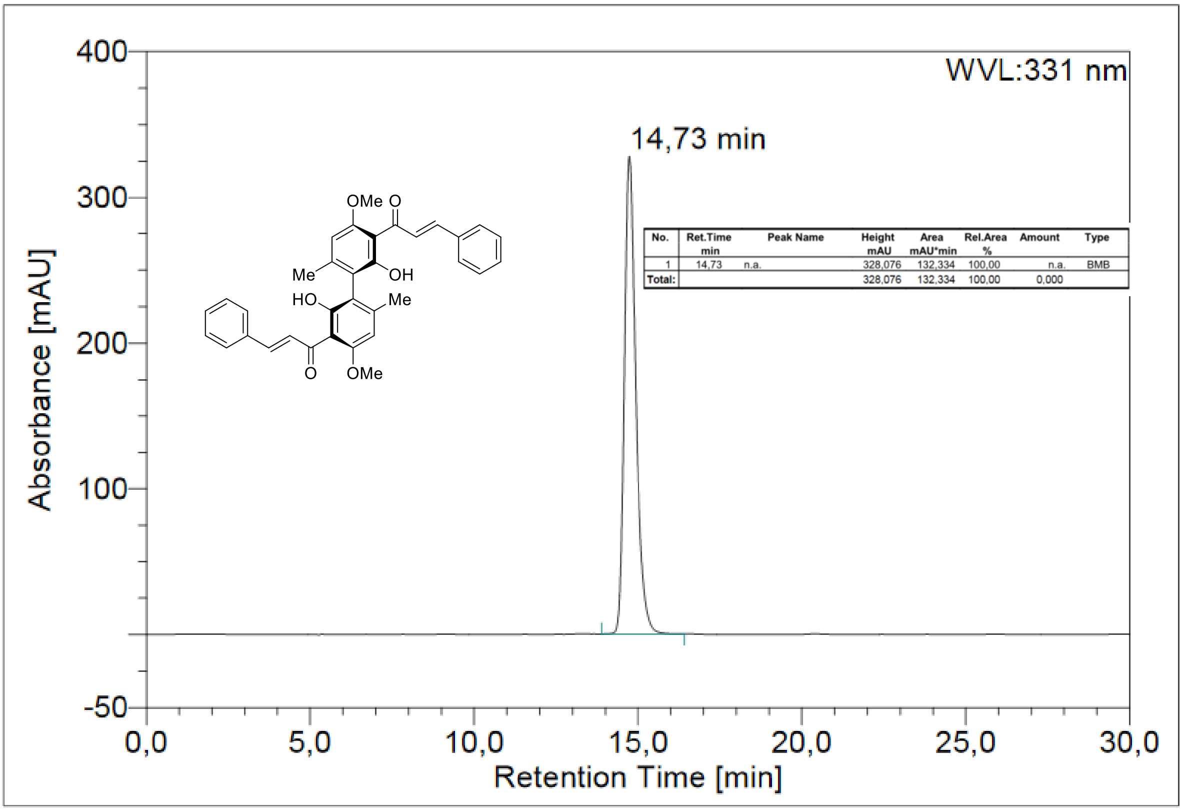

Figure S11: HPLC Chromatogram of bichalcone 2ab >99%*ee* (*S*a). Lux® Amylose-1 (*Phenomenex*) 2504.6 mm, 25 °C, 0.5 mL min^{-1}, 331 nm, *n*-heptane:*i*-propanol 50:50 (v/v): $t_R(S_a)$ = 14.7 min, $t_R(R_a)$ = 24.2 min.

2.1 Stability experiments and purity by reversed phase HPLC

To investigate the stability of bichalcone **2ab**, we submitted the racemic mixture to various conditions.

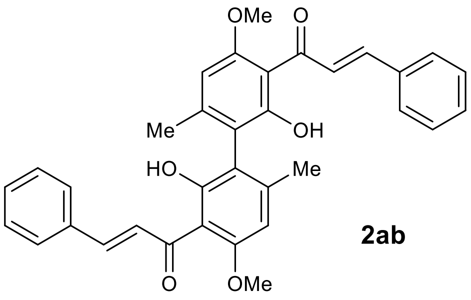

2ab

Method:

Hyperclone 5 µ ODS (C18), 125*4 mm, 120 Å, Fa. Phenomenex, 1 mL/min, A: H_2O; B: Methanol

Gradient: 0-2 min 10% B/90% A

2-15 min	Gradient 10% B/90% A to 100% B/0% A
15-20 min	100% B/0% A
20-21 min	Gradient 100% B/0% A to 10% B/90% A
21-30 min	10% B/90% A

Table S3: Purity results of the stability experiments over a varying time span at different temperatures.

Entry	Variations	Time	Purity [%]
1	22 °C, DMSO	24 h	75.9
2	40 °C, DMSO	24 h	76.6
3	-20 °C, DMSO	6 months	96.2
3	40 °C, MeOH, KOH	24 h	35.6
4	40 °C, MeOH	24 h	89.7

Table S4: Reverse Phase HPLC chromatogram of bichalcone 2ab (*rac*) in DMSO stock solution at 22 °C for 24 h.

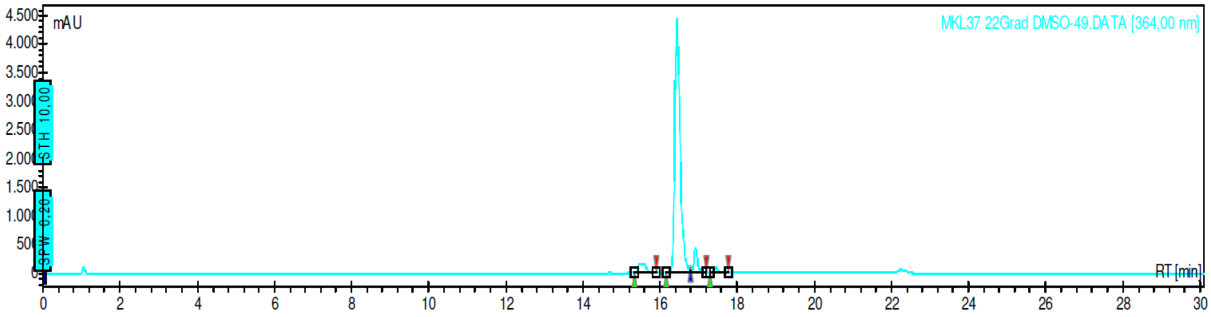

Index	Name	Time [Min]	Quantity [% Area]	Height [mAU]	Area [mAU.Min]	Area % [%]
1	UNKNOWN	15,479	5,01	164,3	39,7	5,008
2	UNKNOWN	16,439	87,93	4437,9	696,2	87,931
4	UNKNOWN	16,905	5,98	428,5	47,3	5,980
3	UNKNOWN	17,439	1,08	78,6	8,6	1,081
Total			100,00	5109,3	791,8	100,000

Table S5: Reverse Phase HPLC chromatogram of bichalcone 2ab (*rac*) in DMSO stock solution at 40 °C for 24 h.

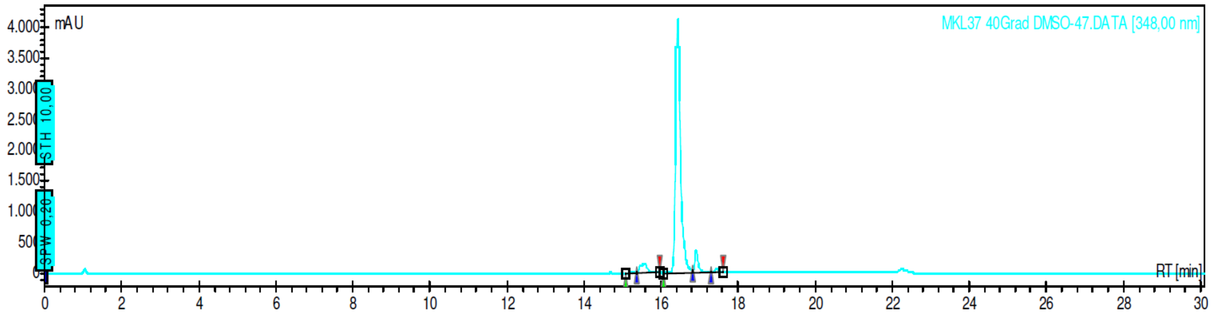

Index	Name	Time [Min]	Quantity [% Area]	Height [mAU]	Area [mAU.Min]	Area % [%]
1	UNKNOWN	15,239	0,23	19,0	1,6	0,234
3	UNKNOWN	15,559	4,99	147,8	34,9	4,986
2	UNKNOWN	16,425	88,23	4142,5	617,8	88,234
4	UNKNOWN	16,905	5,67	367,5	39,7	5,666
5	UNKNOWN	17,425	0,88	60,0	6,2	0,880
Total			100,00	4736,8	700,2	100,000

Table S6: Reverse Phase HPLC chromatogram of bichalcone 2ab (*rac*) in DMSO at -78 °C for 6 months.

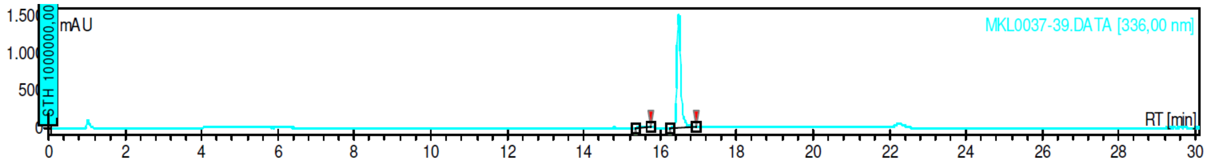

Index	Name	Time [Min]	Quantity [% Area]	Height [mAU]	Area [mAU.Min]	Area % [%]
2	UNKNOWN	15,546	1,91	14,8	3,0	1,910
1	UNKNOWN	16,545	98,09	1353,7	153,2	98,090
Total			100,00	1368,5	156,2	100,000

Table S7: Reverse Phase HPLC chromatogram of bichalcone 2ab (*rac*) in MeOH/aq. KOH solution at 40 °C for 24 h.

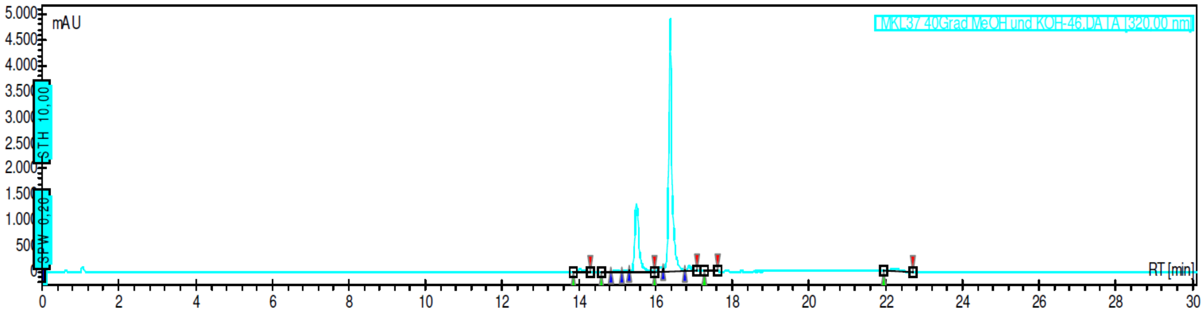

Index	Name	Time [Min]	Quantity [% Area]	Height [mAU]	Area [mAU.Min]	Area % [%]
1	UNKNOWN	14,026	1,04	56,6	7,1	1,043
2	UNKNOWN	14,732	0,11	6,4	0,8	0,113
3	UNKNOWN	14,932	0,41	21,2	2,8	0,413
4	UNKNOWN	15,212	0,24	13,1	1,6	0,238
5	UNKNOWN	15,506	24,66	1318,7	168,8	24,655
6	UNKNOWN	16,119	1,48	89,1	10,1	1,477
7	UNKNOWN	16,372	67,71	4914,3	463,6	67,707
8	UNKNOWN	16,879	1,53	98,2	10,5	1,529
9	UNKNOWN	17,399	0,32	18,4	2,2	0,315
10	UNKNOWN	22,225	2,51	60,7	17,2	2,508
Total			100,00	6596,6	684,7	100,000

Table S8: Reverse Phase HPLC chromatogram of bichalcone 2ab (*rac*) in MeOH at 40 °C for 24 h.

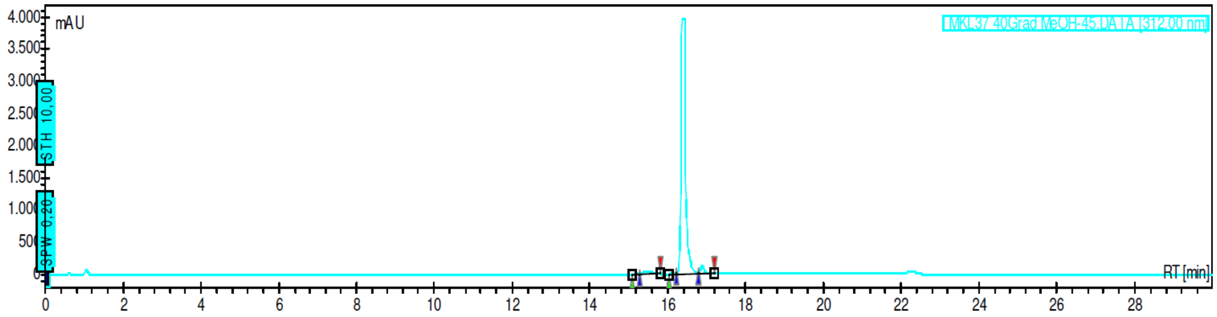

Index	Name	Time [Min]	Quantity [% Area]	Height [mAU]	Area [mAU.Min]	Area % [%]
1	UNKNOWN	15,212	0,13	10,0	0,7	0,129
2	UNKNOWN	15,439	2,19	48,8	11,6	2,187
3	UNKNOWN	16,132	0,18	8,1	0,9	0,177
4	UNKNOWN	16,372	94,89	3972,3	504,6	94,895
5	UNKNOWN	16,879	2,61	128,4	13,9	2,612
Total			100,00	4167,6	531,8	100,000

We additionally assessed the purity of both enantiomers of bichalcone **2ab** by reversed phase HPLC.

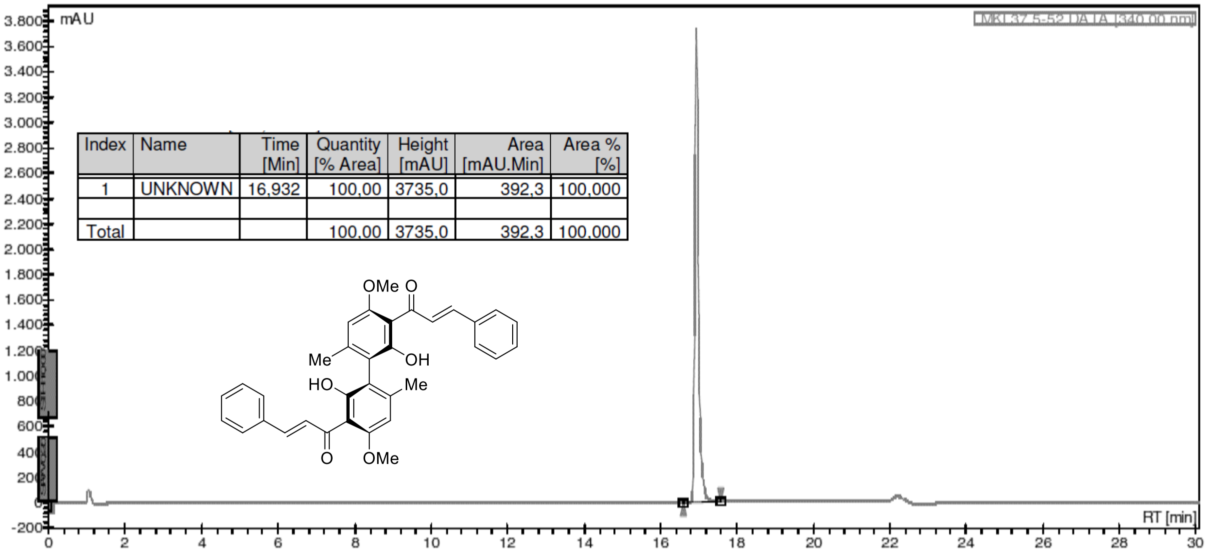

Index	Name	Time [Min]	Quantity [% Area]	Height [mAU]	Area [mAU.Min]	Area % [%]
1	UNKNOWN	16,932	100,00	3735,0	392,3	100,000
Total			100,00	3735,0	392,3	100,000

Figure S12: Reverse Phase HPLC chromatogram of bichalcone 2ab (*S_a*).

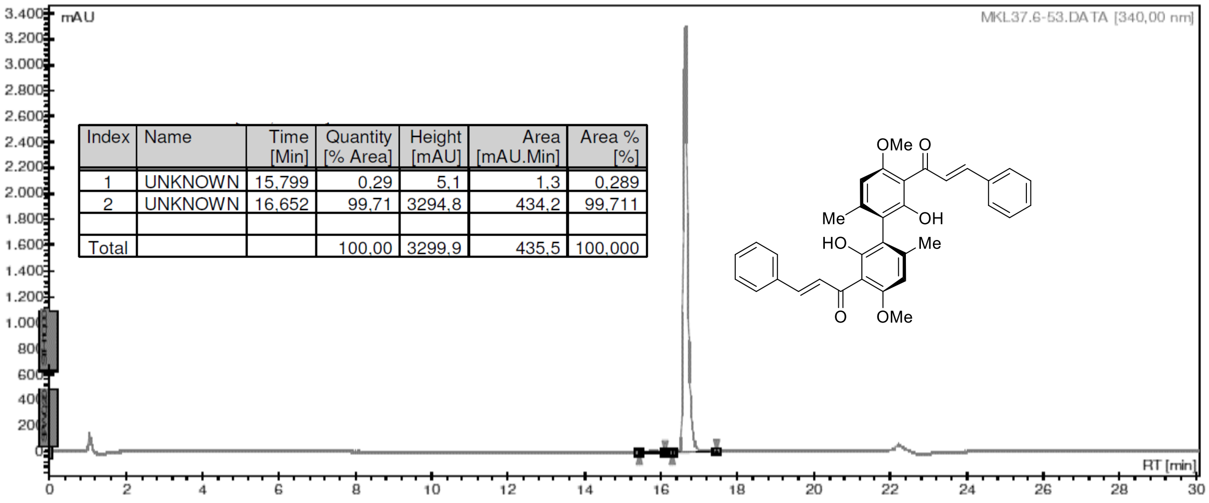

Index	Name	Time [Min]	Quantity [% Area]	Height [mAU]	Area [mAU.Min]	Area % [%]
1	UNKNOWN	15,799	0,29	5,1	1,3	0,289
2	UNKNOWN	16,652	99,71	3294,8	434,2	99,711
Total			100,00	3299,9	435,5	100,000

Figure S13: Reverse Phase HPLC chromatogram of bichalcone 2ab (*R_a*).

3 NMR spectra

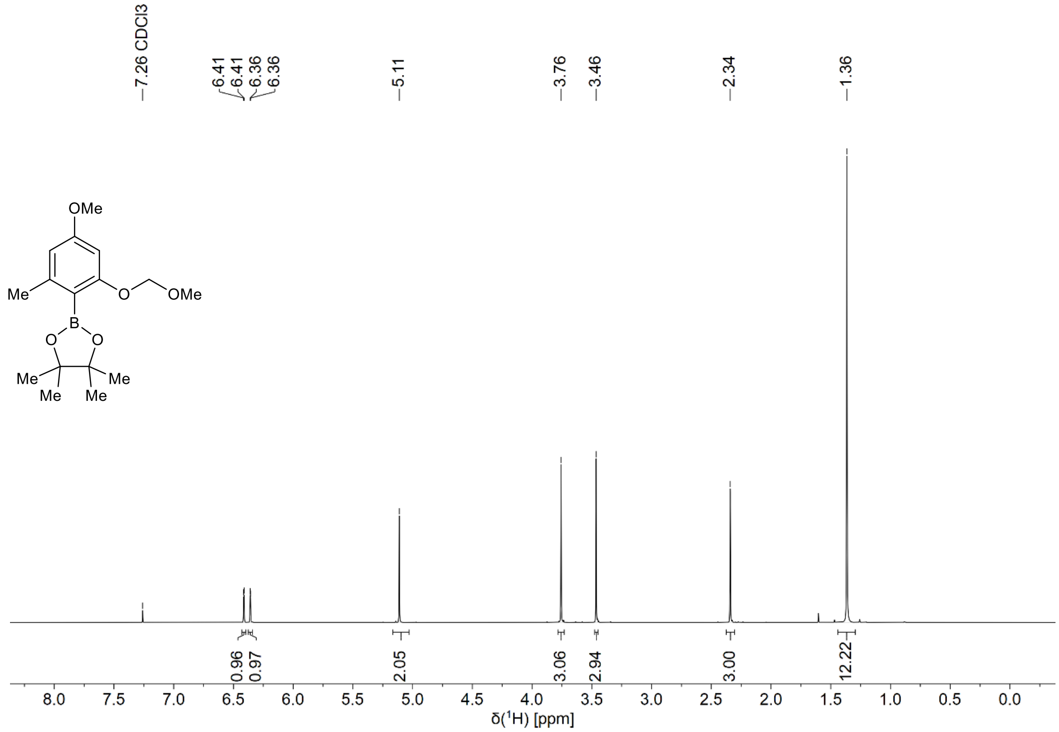

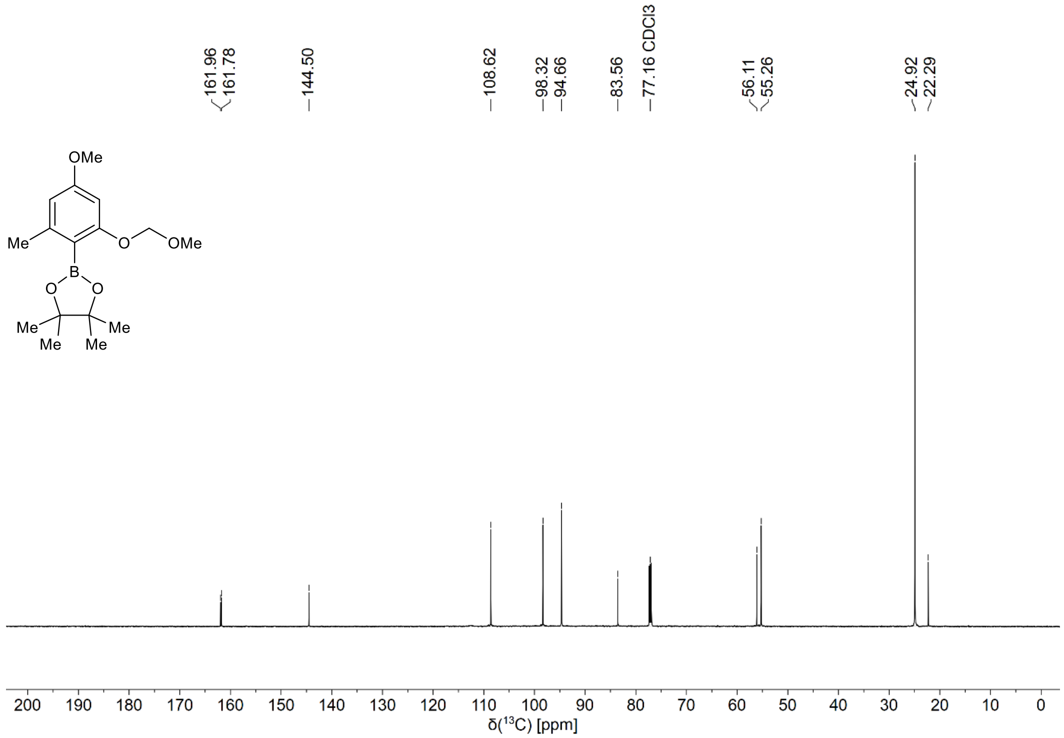

Figure S14: ^{1}H- and ^{13}C-NMR spectra (600 / 151 MHz, $CDCl_3$) of 2-(4-methoxy-2-(methoxymethoxy)-6-methylphenyl)-4,4,5,5-tetramethyl-1,3,2-dioxaborolane (5).

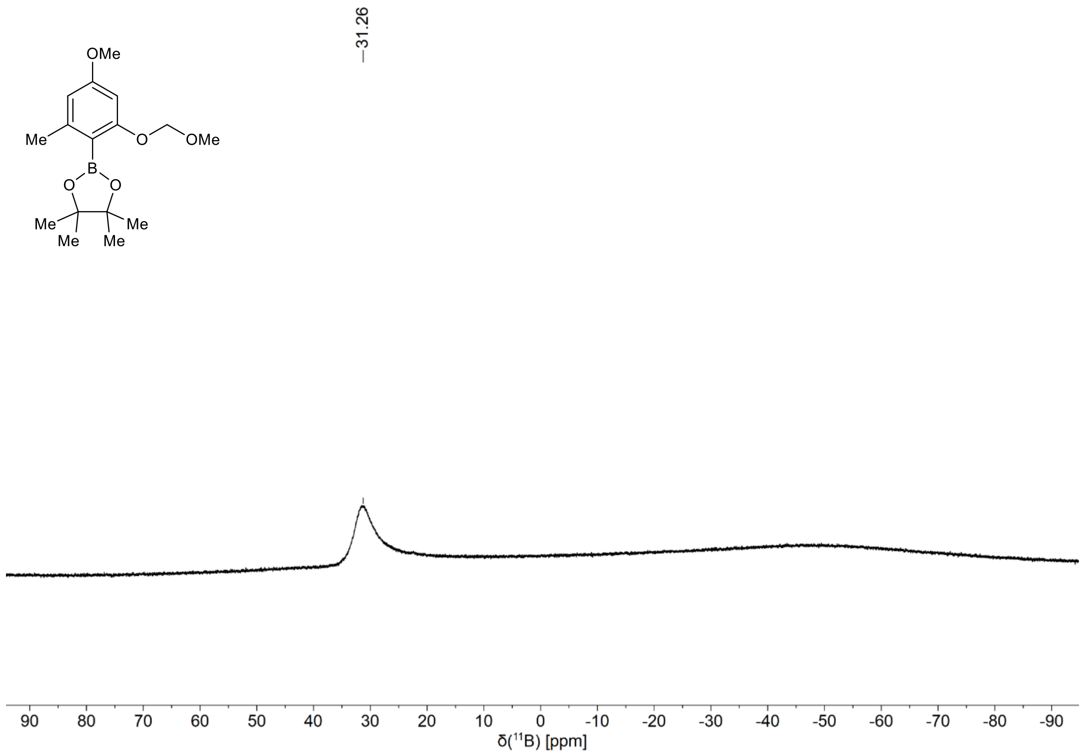

Figure S15: ^{11}B-NMR spectrum (96 MHz, $CDCl_3$) of ArBpin (5).

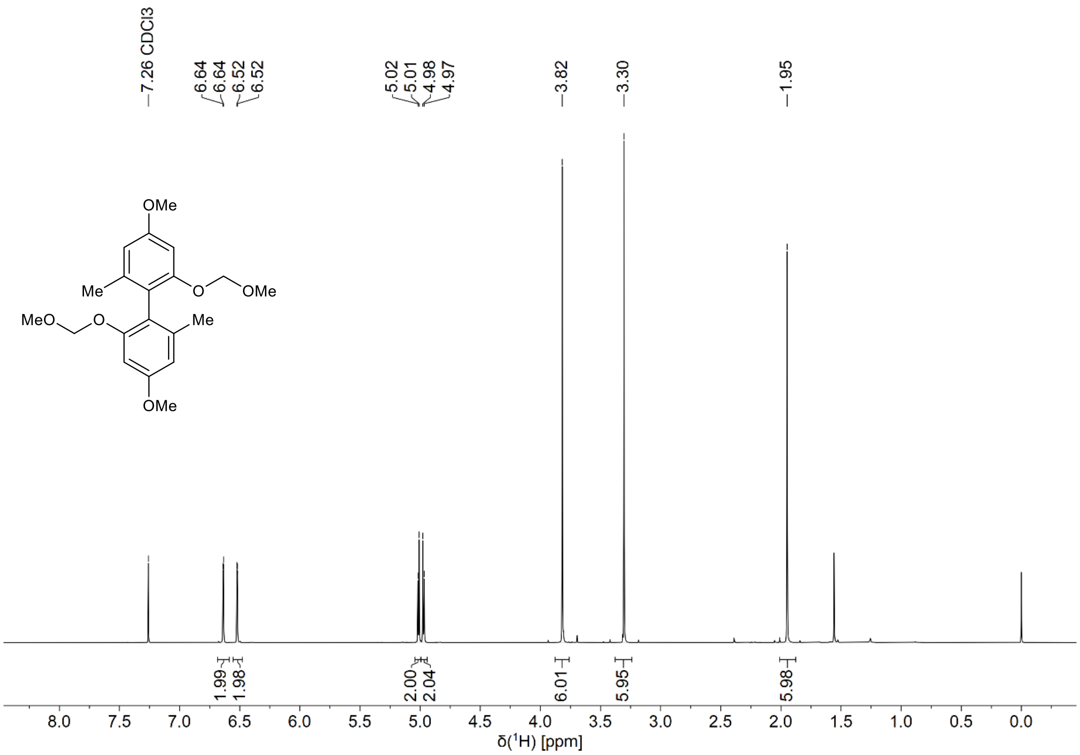

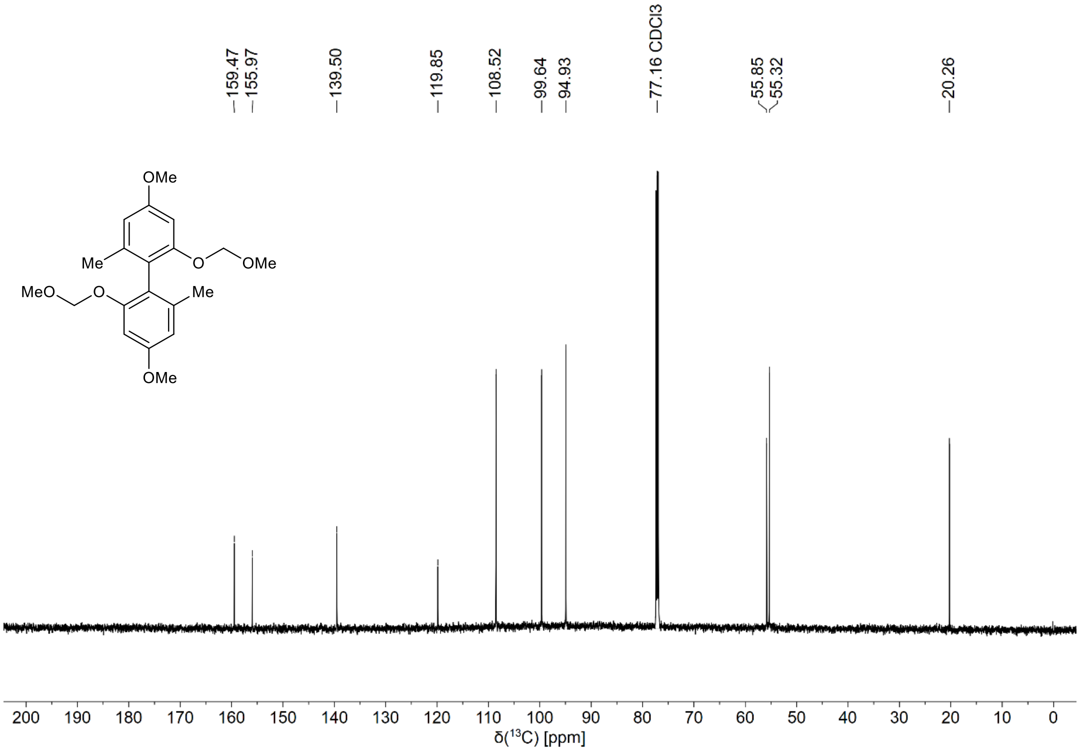

Figure S16: ^{1}H- and ^{13}C-NMR spectra (600 / 151 MHz, $CDCl_3$) of 4,4'-dimethoxy-2,2'-bis(methoxymethoxy)-6,6'-dimethyl-1,1'-biphenyl (6).

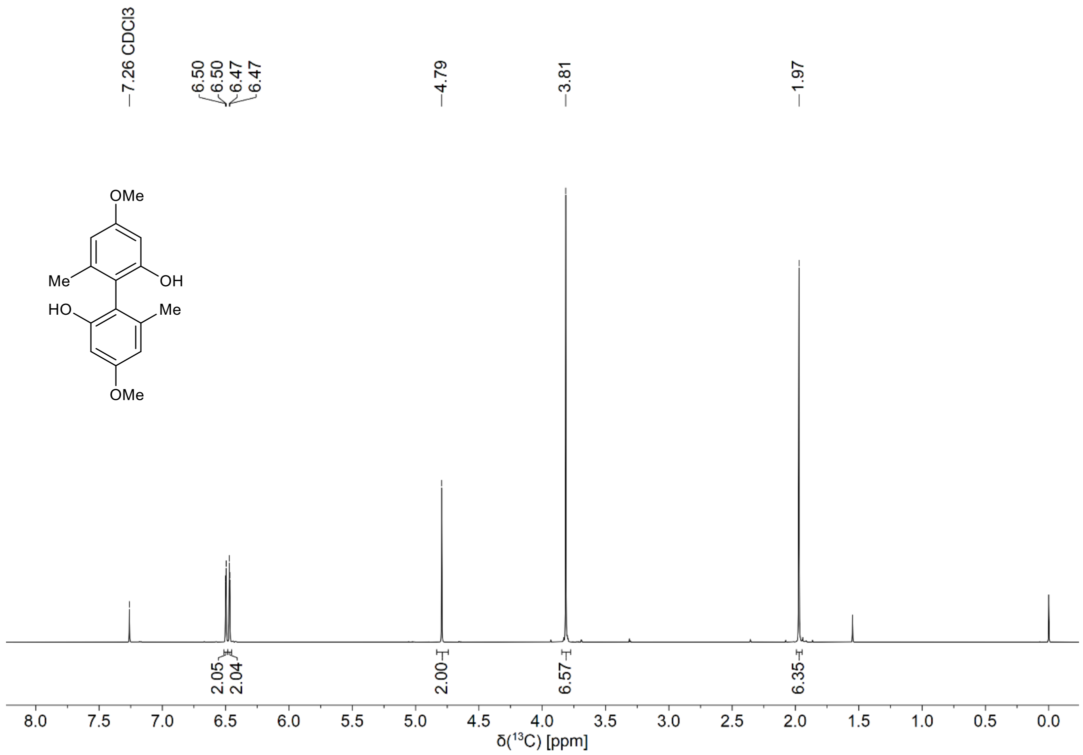

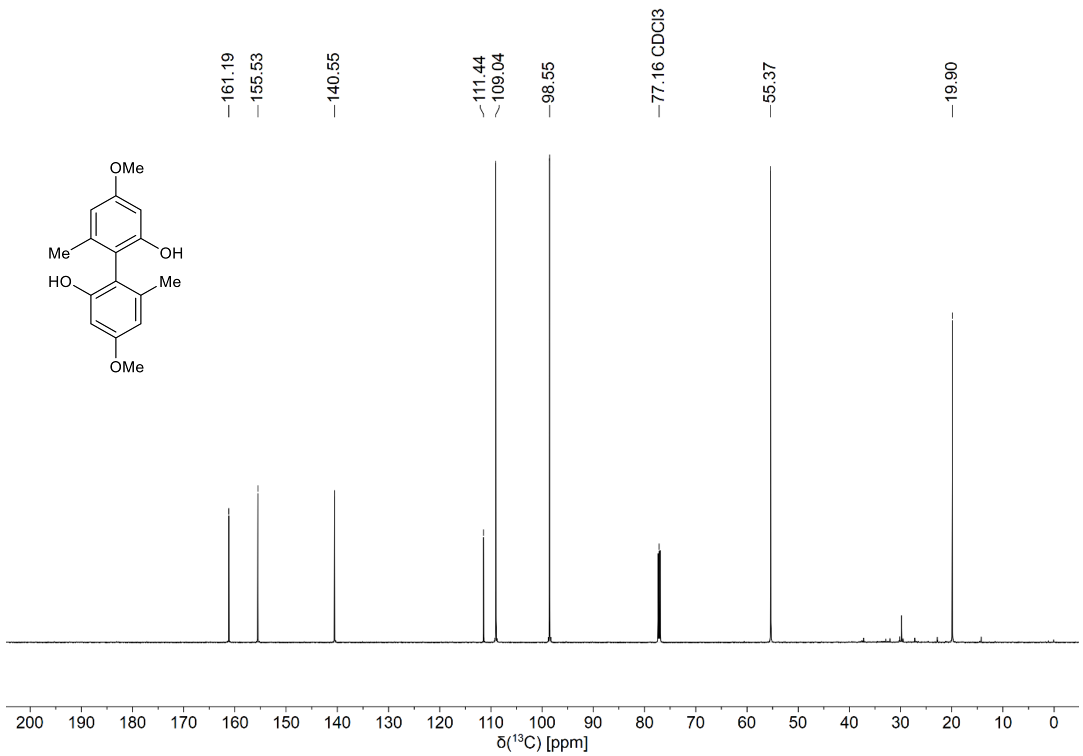

Figure S17: ^{1}H- and ^{13}C-NMR spectra (600 / 151 MHz, $CDCl_3$) of 4,4'-dimethoxy-6,6'-dimethyl-[1,1'-biphenyl]-2,2'-diol (3).

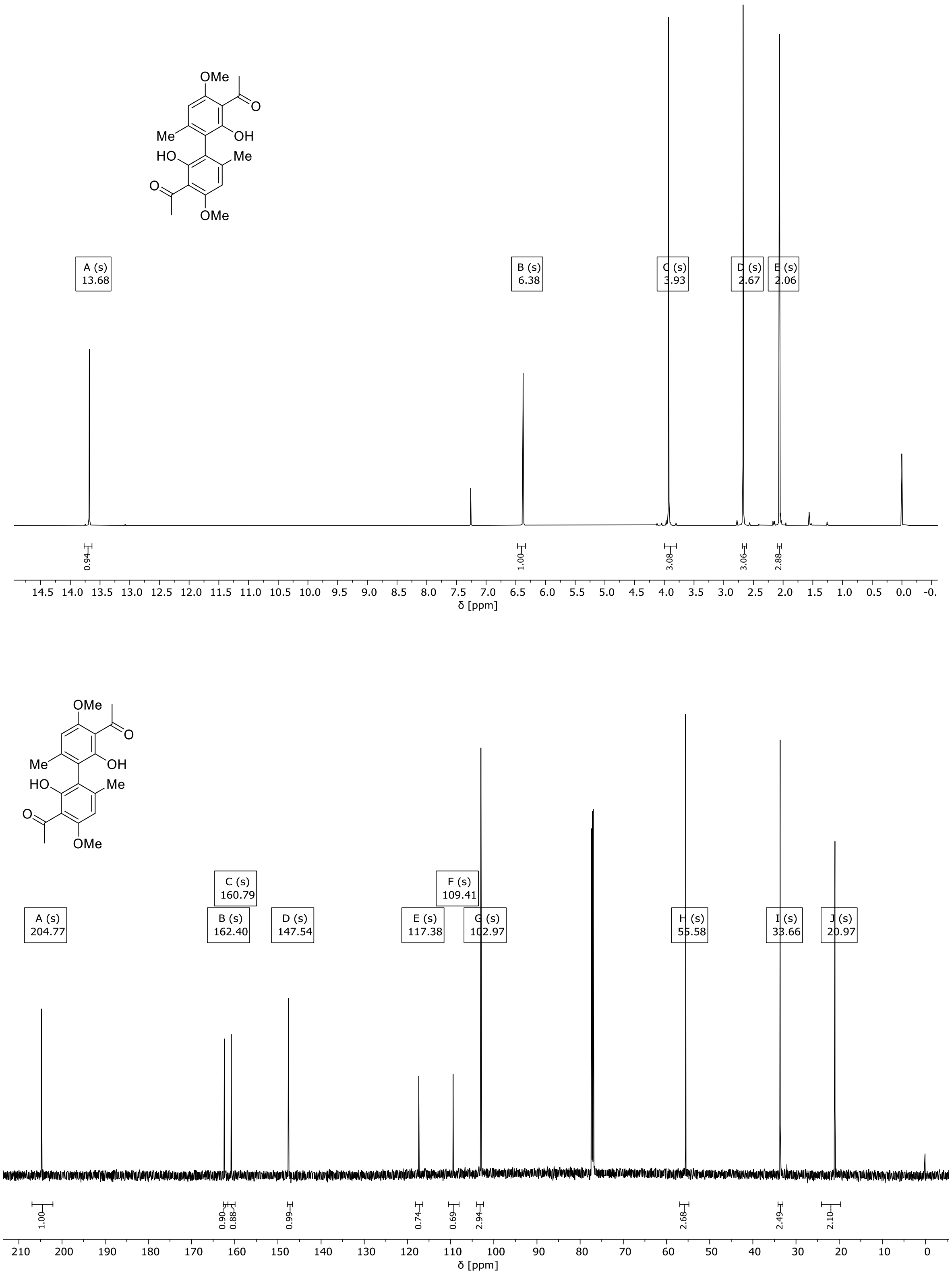

Figure S18: ^{1}H- and ^{13}C-NMR spectra (600 / 151 MHz, $CDCl_3$) of rac-1-(2-hydroxy-6-methoxy-4-methylphenyl)ethan-1-one (7).

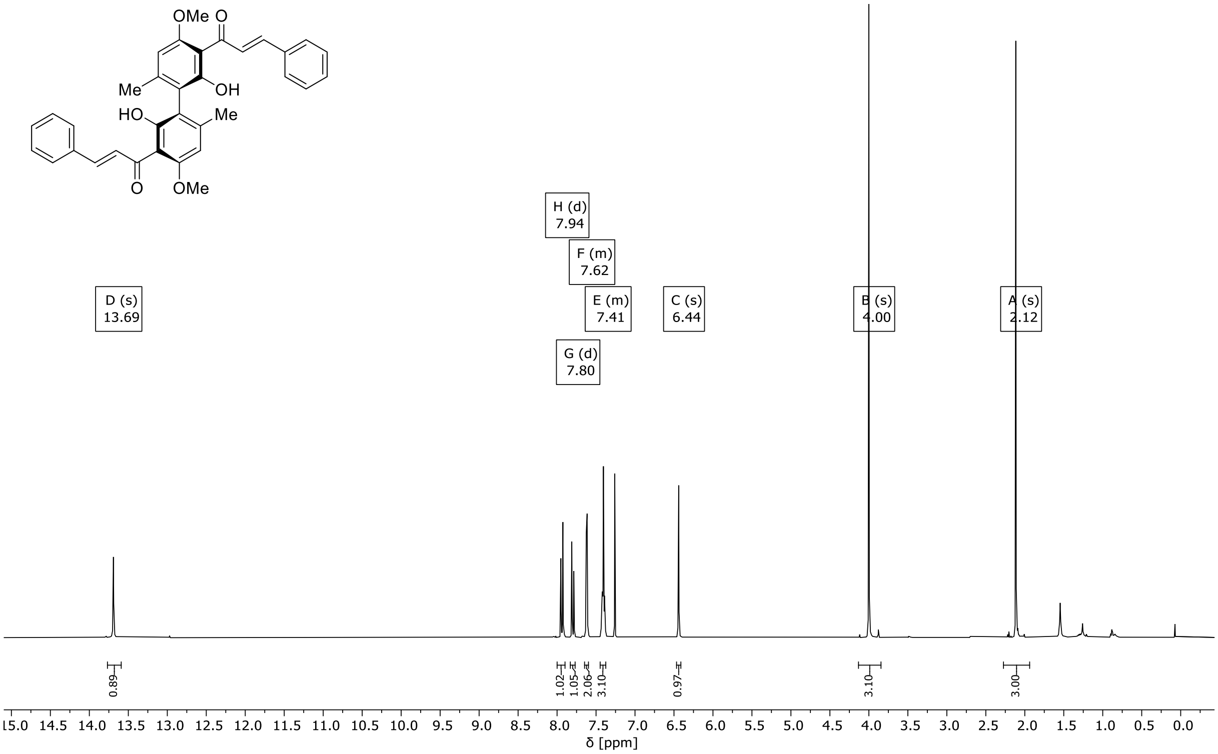

Figure S19: ^{1}H- spectrum (600 MHz, $CDCl_3$) of (+)-(2*E*,2'*E*)-1,1'-(2,2'-dihydroxy-4,4'-dimethoxy-6,6'-dimethyl-[1,1'-biphenyl]-3,3'-diyl)bis(3-phenylprop-2-en-1-one) (2ab, S_a).

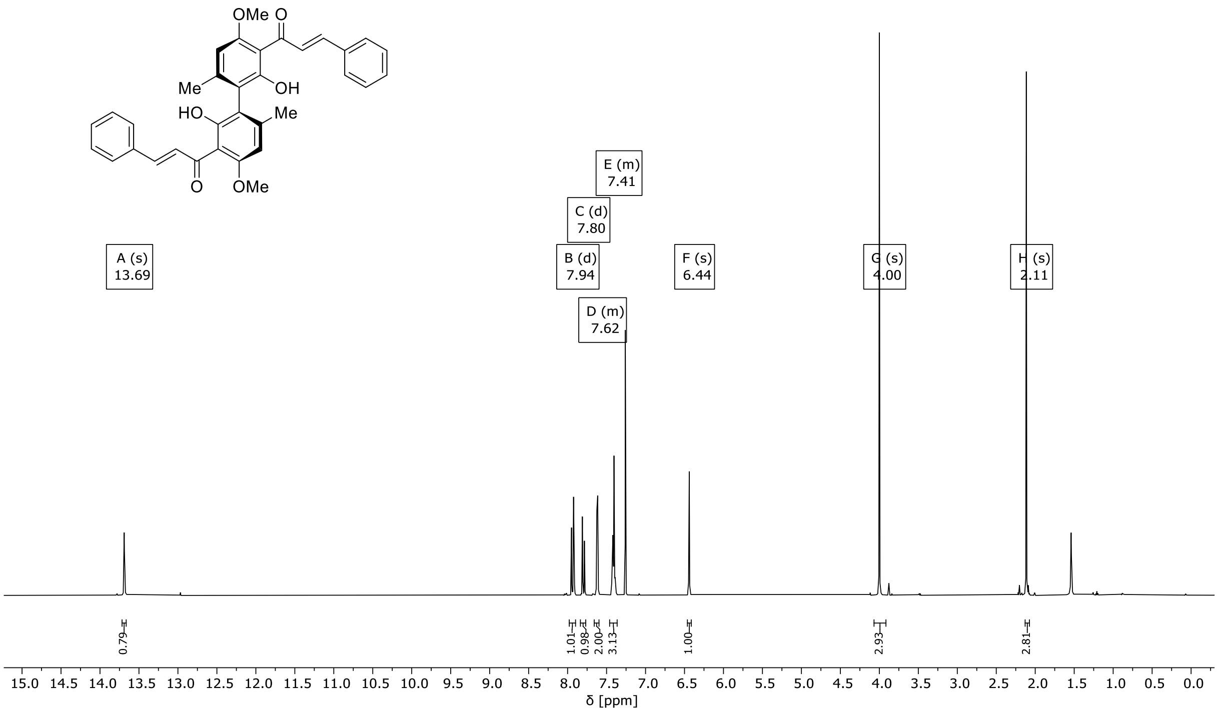

Figure S20: ^{1}H- spectrum (600 MHz, $CDCl_3$) of (−)-(2*E*,2'*E*)-1,1'-(2,2'-dihydroxy-4,4'-dimethoxy-6,6'-dimethyl-[1,1'-biphenyl]-3,3'-diyl)bis(3-phenylprop-2-en-1-one) (2ab, *R*a).

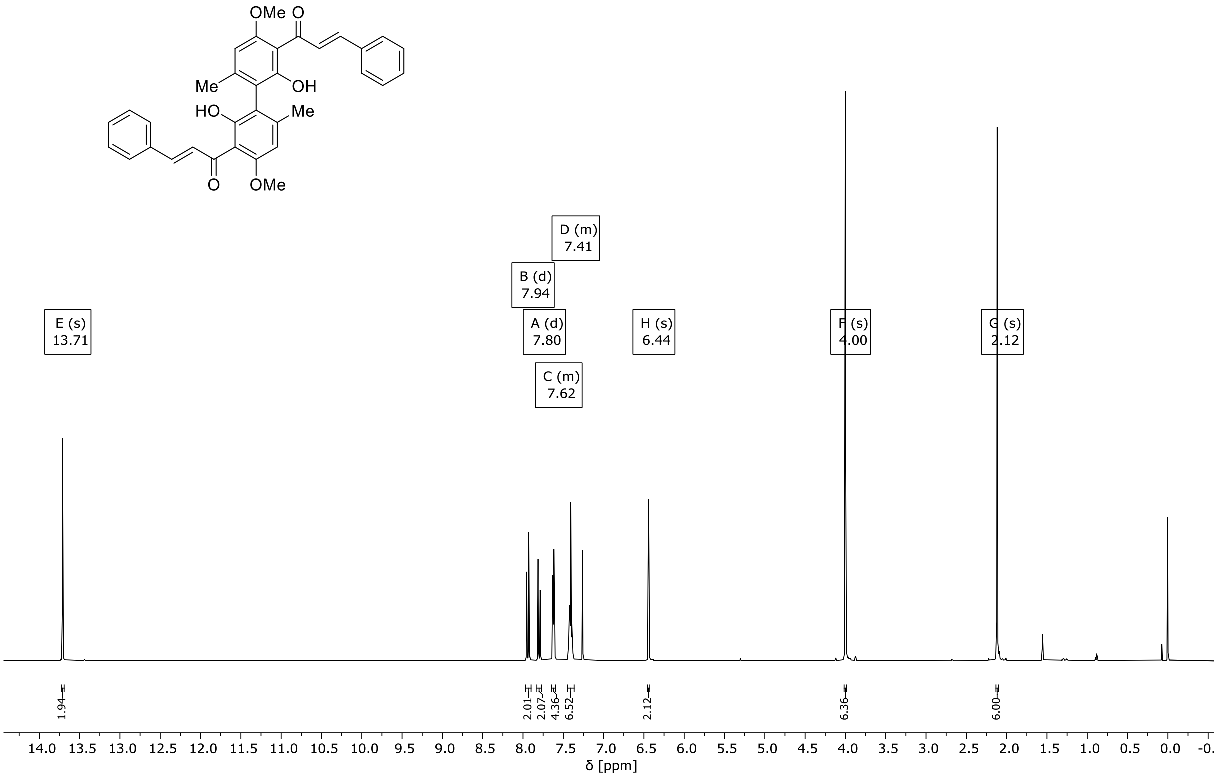

Figure S21: ^{1}H- spectrum (600 MHz, $CDCl_3$) of *rac*-(2*E*,2'*E*)-1,1'-(2,2'-dihydroxy-4,4'-dimethoxy-6,6'-dimethyl-[1,1'-biphenyl]-3,3'-diyl)bis(3-phenylprop-2-en-1-one) (2ab, *rac*).

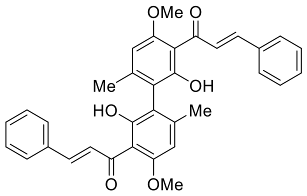

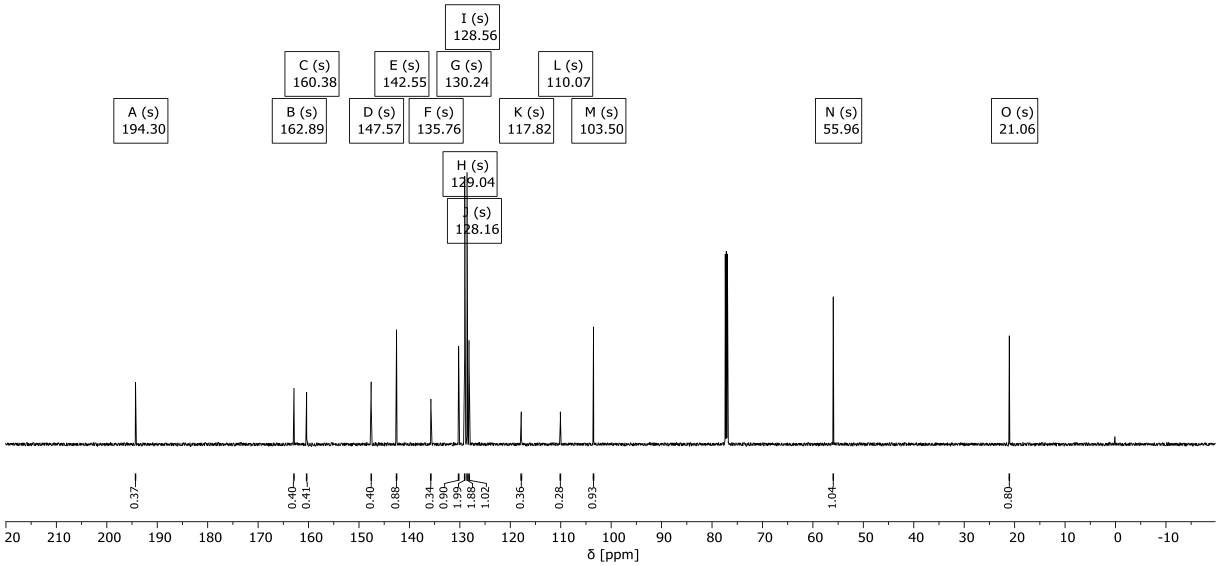

Figure S22: ^{13}C- spectrum (151 MHz, $CDCl_3$) of *rac*-(2*E*,2'*E*)-1,1'-(2,2'-dihydroxy-4,4'-dimethoxy-6,6'-dimethyl-[1,1'-biphenyl]-3,3'-diyl)bis(3-phenylprop-2-en-1-one) (2ab, *rac*).

4 Biological data

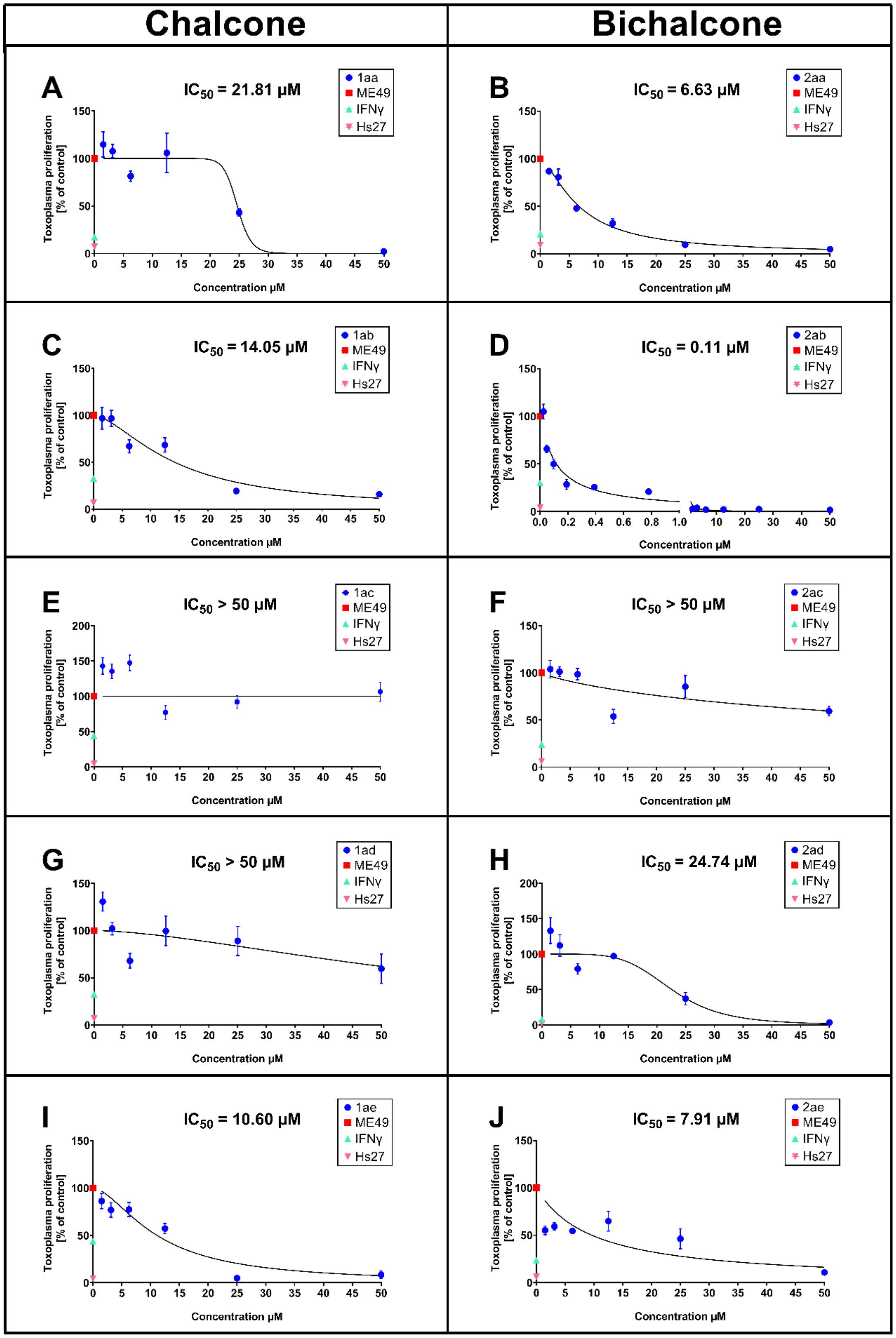

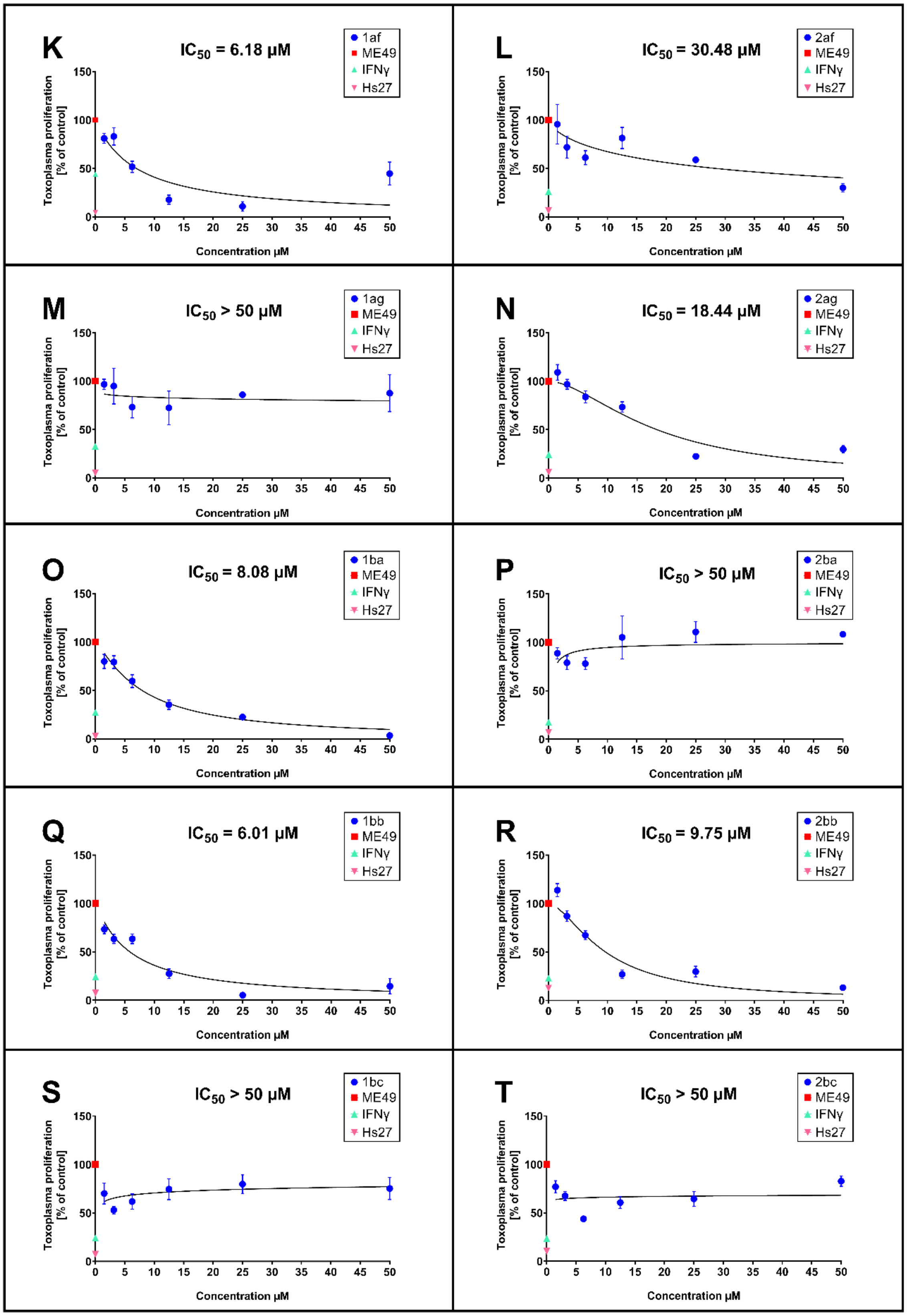
K
IC50 = 6.18 µM
1af
ME49
IFNγ
Hs27
L
IC50 = 30.48 µM
2af
ME49
IFNγ
Hs27
M
IC50 > 50 µM
1ag
ME49
IFNγ
Hs27
N
IC50 = 18.44 µM
2ag
ME49
IFNγ
Hs27
O
IC50 = 8.08 µM
1ba
ME49
IFNγ
Hs27
P
IC50 > 50 µM
2ba
ME49
IFNγ
Hs27
Q
IC50 = 6.01 µM
1bb
ME49
IFNγ
Hs27
R
IC50 = 9.75 µM
2bb
ME49
IFNγ
Hs27
S
IC50 > 50 µM
1bc
ME49
IFNγ
Hs27
T
IC50 > 50 µM
2bc
ME49
IFNγ
Hs27
Toxoplasma proliferation [% of control]
Concentration µM

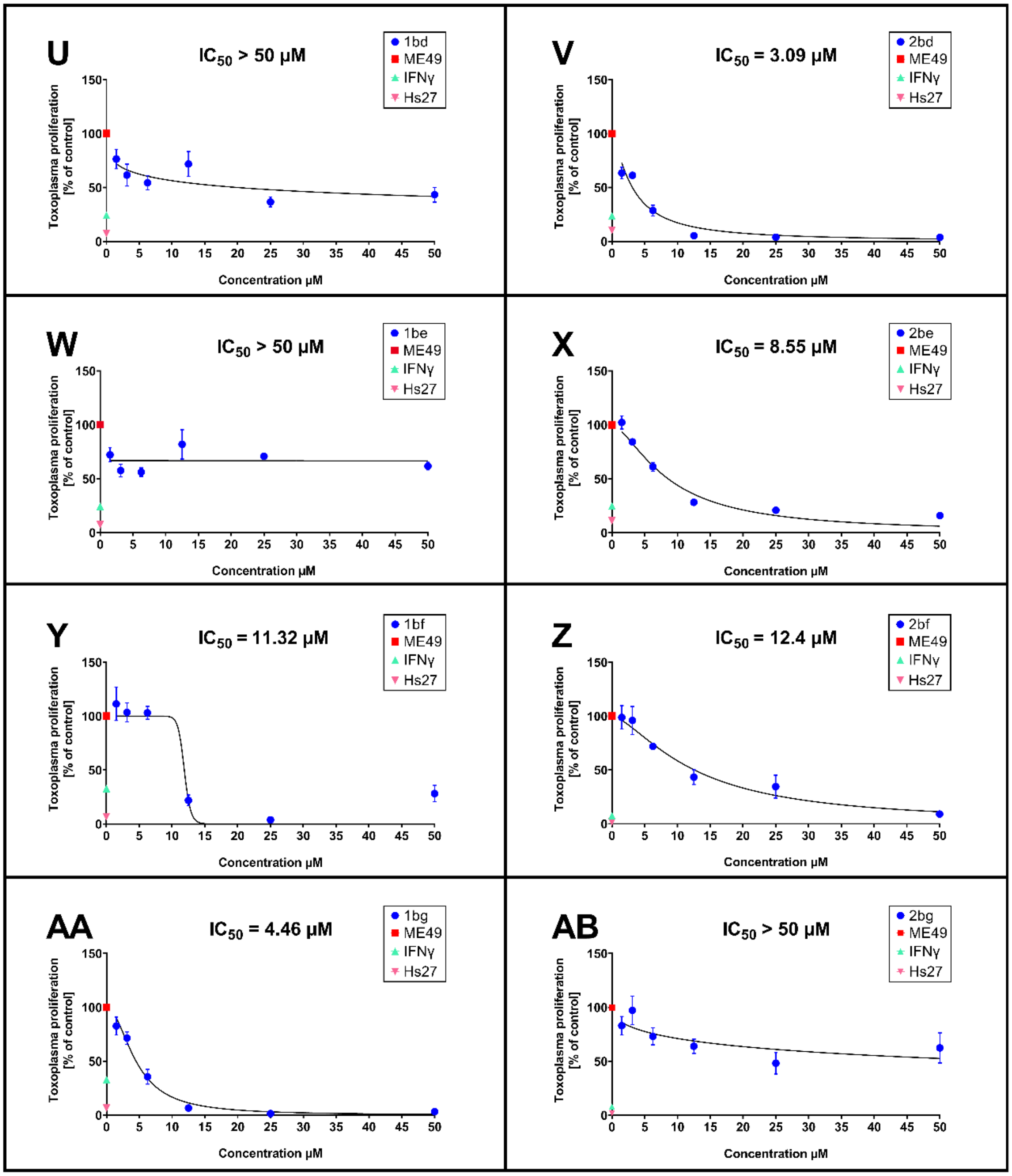

Figure S23: Effects and IC50 values of chalcones and bichalcones on *T. gondii* proliferation. *T. gondii* ME49 tachyzoites were incubated with various concentrations of chalcones and bichalcones and the [^{3}H]-uracil incorporation assay was performed to assess their activity as described in the Materials and Methods. Plots represent nonlinear regression curves and IC50 values of **1aa** (**A**); **2aa** (**B**); **1ab** (**C**); **2ab** (**D**); **1ac** (**E**); **2ac** (**F**); **1ad** (**G**); **2ad** (**H**); **1ae** (**I**); **2ae** (**J**); **1af** (**K**); **2af** (**L**); **1ag** (**M**); **2ag** (**N**); **1ba** (**O**); **2ba** (**P**); **1bb** (**Q**); **2bb** (**R**); **1bc** (**S**); **2bc** (**T**); **1bd** (**U**); **2bd** (**V**); **1be** (**W**); **2be** (**X**); **1bf** (**Y**); **2bf** (**Z**); **1bg** (**AA**); **2bg** (**AB**) against *T. gondii* proliferation. The control samples included untreated and uninfected Hs27 cells (represented by pink triangles), infected Hs27 cells pre-stimulated with IFNγ for 18 h (represented by green triangles) and untreated *T. gondii*-infected Hs27

cells (represented by red squares). Values presented in the tables indicated the means of three independent experiments each performed in duplicate (*n* = 6) ± SEM. IC_{50} values are shown.

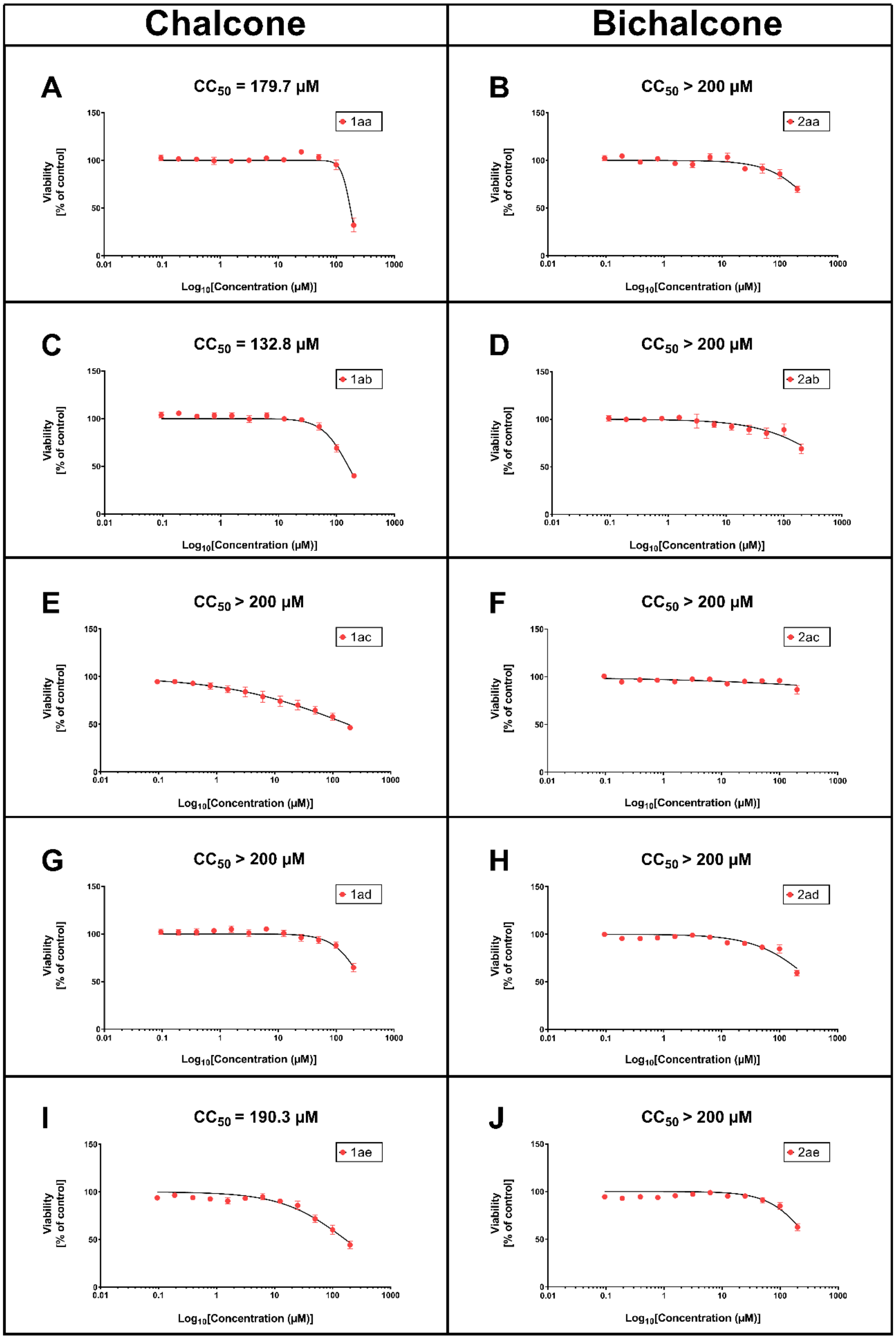
Chalcone
Bichalcone
A
CC50 = 179.7 µM
1aa
B
CC50 > 200 µM
2aa
C
CC50 = 132.8 µM
1ab
D
CC50 > 200 µM
2ab
E
CC50 > 200 µM
1ac
F
CC50 > 200 µM
2ac
G
CC50 > 200 µM
1ad
H
CC50 > 200 µM
2ad
I
CC50 = 190.3 µM
1ae
J
CC50 > 200 µM
2ae
Viability [% of control]
Log10[Concentration (µM)]
0
50
100
150
0.01
0.1
1
10
100
1000

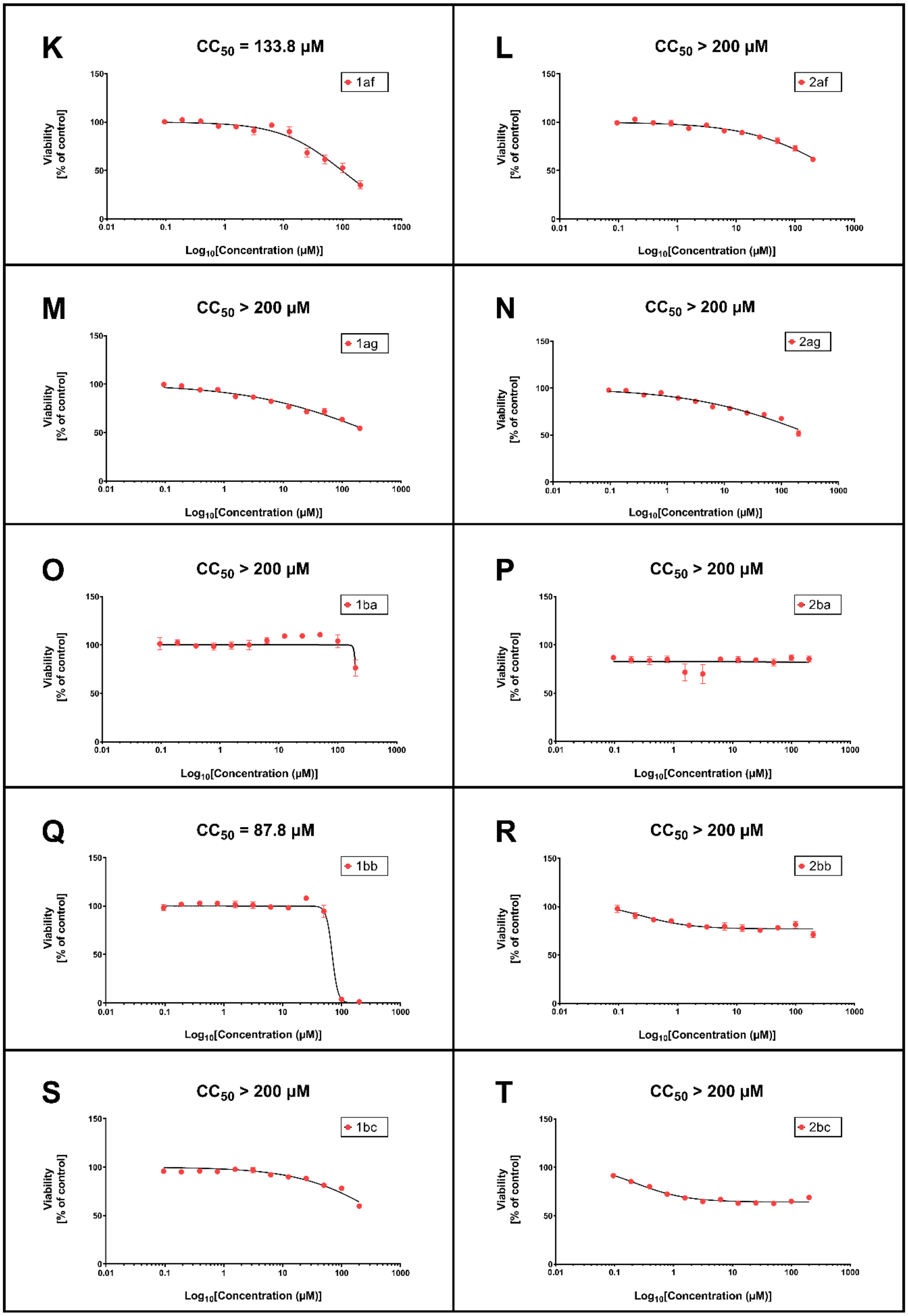

K
CC50 = 133.8 μM
1af
L
CC50 > 200 μM
2af
M
CC50 > 200 μM
1ag
N
CC50 > 200 μM
2ag
O
CC50 > 200 μM
1ba
P
CC50 > 200 μM
2ba
Q
CC50 = 87.8 μM
1bb
R
CC50 > 200 μM
2bb
S
CC50 > 200 μM
1bc
T
CC50 > 200 μM
2bc
Viability [% of control]
150
100
50
0
0.01
0.1
1
10
100
1000
Log10[Concentration (μM)]

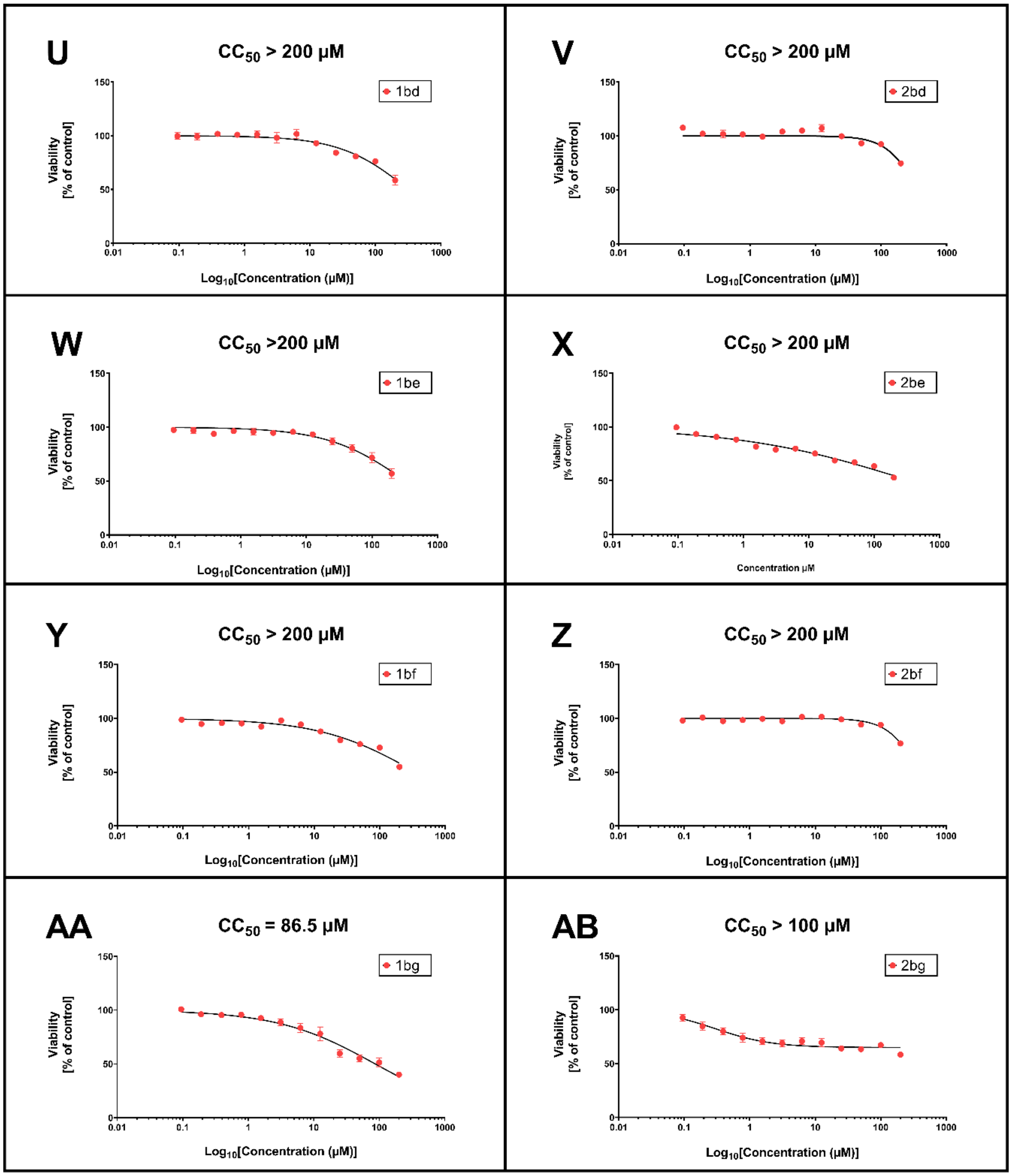

Figure S24: Effect of chalcones and bichalcones on the viability of human Hs27 fibroblasts. Cytotoxic effect of **1aa** (**A**); **2aa** (**B**); **1ab** (**C**); **2ab** (**D**); **1ac** (**E**); **2ac** (**F**); **1ad** (**G**); **2ad** (**H**); **1ae** (**I**); **2ae** (**J**); **1af** (**K**); **2af** (**L**); **1ag** (**M**); **2ag** (**N**); **1ba** (**O**); **2ba** (**P**); **1bb** (**Q**); **2bb** (**R**); **1bc** (**S**); **2bc** (**T**); **1bd** (**U**); **2bd** (**V**); **1be** (**W**); **2be** (**X**); **1bf** (**Y**); **2bf** (**Z**); **1bg** (**AA**); **2bg** (**AB**) against human cell lines Hs27 as determined by MTT assay. 100% growth control DMSO, 0% growth control staurosporine. Values shown in the figures represent the means of three independent experiments each done in duplicate (n = 6) ± SEM.

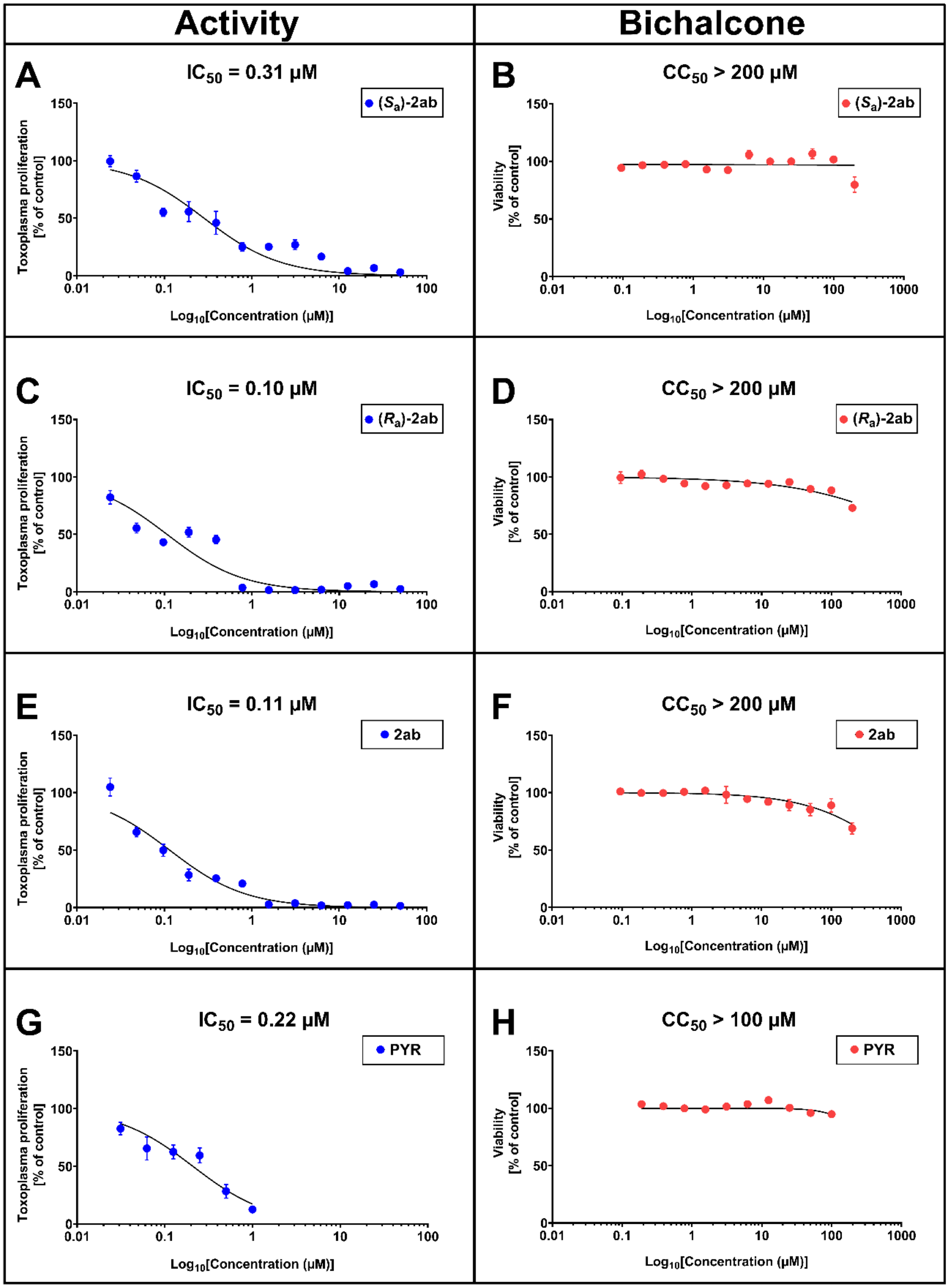

Figure S25: The anti-toxoplasma activity and cytotoxicity of the pure enantiomers (*S*a)-2ab and (*R*a)-2ab and the racemic solution 2ab compared with pyrimethamine.

The inhibitory activities of the enantiomers **(*S*a)-2ab** (**A**), **(*R*a)-2ab** (**C**), the racemic solution **2ab** (**E**) and **PYR** (**G**) were determined by the *T. gondii in vitro* inhibition assay via the [^{3}H]-uracil incorporation into the RNA of the parasite. The cytotoxicity of **(*S*a)-2ab** (**B**), **(*R*a)-2ab** (**D**), the racemic solution **2ab** (**F**) and **PYR** (**H**) was measured by MTT assays. Data shown are from the means of three independent experiments each performed in duplicate (*n* = 6) ± SEM. IC_{50} and CC_{50} values of each compound are shown.

5 SwissADME

Table S9. SwissADME physicochemical, pharmacokinetics and drug-likeness predictions for chalchones 1aa–1bg and bichalcone 2aa–2bg – part 1.

Molecule	Canonical SMILES
**1aa**	COc1ccc(cc1)/C=C/C(=O)c1c(O)cc(cc1OC)C
**1ab**	COc1cc(C)cc(c1C(=O)/C=C/c1ccccc1)O
**1ac**	COc1cc(C)cc(c1C(=O)/C=C/c1ccc(cc1)N(C)C)O
**1ad**	COc1cc(C)cc(c1C(=O)/C=C/c1ccc(cc1)C(F)(F)F)O
**1ae**	COc1cc(C)cc(c1C(=O)/C=C/c1ccc(cc1)Br)O
**1af**	COc1cc(C)cc(c1C(=O)/C=C/c1cccc(c1)Br)O
**1ag**	COc1cc(C)cc(c1C(=O)/C=C/c1ccccc1Br)O
**1ba**	COc1ccc(cc1)/C=C/C(=O)c1c(O)cc(cc1OC)OC
**1bb**	COc1cc(O)c(c(c1)OC)C(=O)/C=C/c1ccccc1
**1bc**	COc1cc(O)c(c(c1)OC)C(=O)/C=C/c1ccc(cc1)N(C)C
**1bd**	COc1cc(O)c(c(c1)OC)C(=O)/C=C/c1ccc(cc1)C(F)(F)F
**1be**	COc1cc(O)c(c(c1)OC)C(=O)/C=C/c1ccc(cc1)Br
**1bf**	COc1cc(O)c(c(c1)OC)C(=O)/C=C/c1cccc(c1)Br
**1bg**	COc1cc(O)c(c(c1)OC)C(=O)/C=C/c1ccccc1Br
**2aa**	COc1cc(C)c(c(c1C(=O)/C=C/c1ccc(cc1)OC)O)c1c(C)cc(c(c1O)C(=O)/C=C/c1ccc(cc1)OC)OC
**2ab**	COc1cc(C)c(c(c1C(=O)/C=C/c1ccccc1)O)c1c(C)cc(c(c1O)C(=O)/C=C/c1ccccc1)OC
**2ac**	COc1cc(C)c(c(c1C(=O)/C=C/c1ccc(cc1)N(C)C)O)c1c(C)cc(c(c1O)C(=O)/C=C/c1ccc(cc1)N(C)C)OC
**2ad**	COc1cc(C)c(c(c1C(=O)/C=C/c1ccc(cc1)C(F)(F)F)O)c1c(C)cc(c(c1O)C(=O)/C=C/c1ccc(cc1)C(F)(F)F)OC
**2ae**	COc1cc(C)c(c(c1C(=O)/C=C/c1ccc(cc1)Br)O)c1c(C)cc(c(c1O)C(=O)/C=C/c1ccc(cc1)Br)OC
**2af**	COc1cc(C)c(c(c1C(=O)/C=C/c1cccc(c1)Br)O)c1c(C)cc(c(c1O)C(=O)/C=C/c1cccc(c1)Br)OC
**2ag**	COc1cc(C)c(c(c1C(=O)/C=C/c1ccccc1Br)O)c1c(C)cc(c(c1O)C(=O)/C=C/c1ccccc1Br)OC
**2ba**	COc1cc(OC)c(c(c1c1c(OC)cc(c(c1O)C(=O)/C=C/c1ccc(cc1)OC)OC)O)C(=O)/C=C/c1ccc(cc1)OC
**2bb**	COc1cc(OC)c(c(c1c1c(OC)cc(c(c1O)C(=O)/C=C/c1ccccc1)OC)O)C(=O)/C=C/c1ccccc1
**2bc**	COc1cc(OC)c(c(c1c1c(OC)cc(c(c1O)C(=O)/C=C/c1ccc(cc1)N(C)C)OC)O)C(=O)/C=C/c1ccc(cc1)N(C)C
**2bd**	COc1cc(OC)c(c(c1c1c(OC)cc(c(c1O)C(=O)/C=C/c1ccc(cc1)C(F)(F)F)OC)O)C(=O)/C=C/c1ccc(cc1)C(F)(F)F
**2be**	COc1cc(OC)c(c(c1c1c(OC)cc(c(c1O)C(=O)/C=C/c1ccc(cc1)Br)OC)O)C(=O)/C=C/c1ccc(cc1)Br
**2bf**	COc1cc(OC)c(c(c1c1c(OC)cc(c(c1O)C(=O)/C=C/c1cccc(c1)Br)OC)O)C(=O)/C=C/c1cccc(c1)Br
**2bg**	COc1cc(OC)c(c(c1c1c(OC)cc(c(c1O)C(=O)/C=C/c1ccccc1Br)OC)O)C(=O)/C=C/c1ccccc1Br

Table S10. SwissADME physicochemical, pharmacokinetics and drug-likeness predictions for chalchones 1aa–1bg and bichalcone 2aa–2bg – part 2.

Molecule	Formula	MW	#Heavy atoms	#Aromatic heavy atoms	Fraction Csp3
**1aa**	C18H18O4	298.33	22	12	0.17
**1ab**	C17H16O3	268.31	20	12	0.12
**1ac**	C19H21NO3	311.37	23	12	0.21
**1ad**	C18H15F3O3	336.31	24	12	0.17
**1ae**	C17H15BrO3	347.2	21	12	0.12
**1af**	C17H15BrO3	347.2	21	12	0.12
**1ag**	C17H15BrO3	347.2	21	12	0.12
**1ba**	C18H18O5	314.33	23	12	0.17
**1bb**	C17H16O4	284.31	21	12	0.12
**1bc**	C19H21NO4	327.37	24	12	0.21
**1bd**	C18H15F3O4	352.3	25	12	0.17
**1be**	C17H15BrO4	363.2	22	12	0.12
**1bf**	C17H15BrO4	363.2	22	12	0.12
**1bg**	C17H15BrO4	363.2	22	12	0.12
**2aa**	C36H34O8	594.65	44	24	0.17
**2ab**	C34H30O6	534.6	40	24	0.12
**2ac**	C38H40N2O6	620.73	46	24	0.21
**2ad**	C36H28F6O6	670.59	48	24	0.17
**2ae**	C34H28Br2O6	692.39	42	24	0.12
**2af**	C34H28Br2O6	692.39	42	24	0.12
**2ag**	C34H28Br2O6	692.39	42	24	0.12
**2ba**	C36H34O10	626.65	46	24	0.17
**2bb**	C34H30O8	566.6	42	24	0.12
**2bc**	C38H40N2O8	652.73	48	24	0.21
**2bd**	C36H28F6O8	702.59	50	24	0.17
**2be**	C34H28Br2O8	724.39	44	24	0.12
**2bf**	C34H28Br2O8	724.39	44	24	0.12
**2bg**	C34H28Br2O8	724.39	44	24	0.12

Table S11. SwissADME physicochemical, pharmacokinetics and drug-likeness predictions for chalchones 1aa–1bg and bichalcone 2aa–2bg – part 3.

Molecule	#Rotatable bonds	#H-bond acceptors	#H-bond donors	MR	TPSA
**1aa**	5	4	1	86.22	55.76
**1ab**	4	3	1	79.73	46.53
**1ac**	5	3	1	93.94	49.77
**1ad**	5	6	1	84.73	46.53
**1ae**	4	3	1	87.43	46.53
**1af**	4	3	1	87.43	46.53
**1ag**	4	3	1	87.43	46.53
**1ba**	6	5	1	87.75	64.99
**1bb**	5	4	1	81.26	55.76
**1bc**	6	4	1	95.46	59
**1bd**	6	7	1	86.26	55.76
**1be**	5	4	1	88.96	55.76
**1bf**	5	4	1	88.96	55.76
**1bg**	5	4	1	88.96	55.76
**2aa**	11	8	2	171.44	111.52
**2ab**	9	6	2	158.45	93.06
**2ac**	11	6	2	186.87	99.54
**2ad**	11	12	2	168.46	93.06
**2ae**	9	6	2	173.85	93.06
**2af**	9	6	2	173.85	93.06
**2ag**	9	6	2	173.85	93.06
**2ba**	13	10	2	174.49	129.98
**2bb**	11	8	2	161.5	111.52
**2bc**	13	8	2	189.92	118
**2bd**	13	14	2	171.51	111.52
**2be**	11	8	2	176.9	111.52
**2bf**	11	8	2	176.9	111.52
**2bg**	11	8	2	176.9	111,52

Table S12. SwissADME physicochemical, pharmacokinetics and drug-likeness predictions for chalchones 1aa–1bg and bichalcone 2aa–2bg – part 4.

Molecule	iLOGP	XLOGP3	WLOGP	MLOGP	Silicos-IT Log P	Consensus Log P
**1aa**	3.35	4.19	3.51	2.31	4.06	3.48
**1ab**	2.39	4.22	3.5	2.66	4	3.35
**1ac**	2.61	4.35	3.56	2.55	3.69	3.35
**1ad**	2.73	5.11	5.67	3.51	5.08	4.42
**1ae**	2.84	4.91	4.26	3.28	4.68	3.99
**1af**	2.82	4.91	4.26	3.28	4.68	3.99
**1ag**	2.76	4.91	4.26	3.28	4.68	3.98
**1ba**	2.98	3.8	3.21	1.75	3.6	3.07
**1bb**	2.63	3.83	3.2	2.08	3.54	3.06
**1bc**	3.44	3.95	3.26	1.99	3.24	3.17
**1bd**	3.05	4.71	5.37	2.93	4.64	4.14
**1be**	2.74	4.52	3.96	2.7	4.22	3.63
**1bf**	3.19	4.52	3.96	2.7	4.22	3.72
**1bg**	3.05	4.52	3.96	2.7	4.22	3.69
**2aa**	4.38	8.09	6.99	2.96	8.19	6.12
**2ab**	4.4	8.15	6.97	3.65	8	6.24
**2ac**	4.45	8.4	7.11	3.31	7.5	6.15
**2ad**	4.6	9.92	11.32	5.07	10.26	8.23
**2ae**	4.13	9.53	8.5	4.72	9.38	7.25
**2af**	4.93	9.53	8.5	4.72	9.38	7.41
**2ag**	4.7	9.53	8.5	4.72	9.38	7.36
**2ba**	4.56	7.3	6.39	1.94	7.3	5.5
**2bb**	4.36	7.36	6.37	2.6	7.1	5.56
**2bc**	4.9	7.61	6.51	2.3	6.61	5.59
**2bd**	4.93	9.13	10.72	3.75	9.37	7.58
**2be**	4.76	8.74	7.9	3.67	8.48	6.71
**2bf**	5.03	8.74	7.9	3.67	8.48	6.76
**2bg**	5.1	8.74	7.9	3.67	8.48	6.78

Table S13. SwissADME physicochemical, pharmacokinetics and drug-likeness predictions for chalchones 1aa–1bg and bichalcone 2aa–2bg – part 5.

Molecule	ESOL Log S	ESOL Solubility (mg/ml)	ESOL Solubility (mol/l)	ESOL Class
**1aa**	-4.4	1.18E-02	3.95E-05	Moderately soluble
**1ab**	-4.34	1.22E-02	4.55E-05	Moderately soluble
**1ac**	-4.57	8.44E-03	2.71E-05	Moderately soluble
**1ad**	-5.18	2.20E-03	6.54E-06	Moderately soluble
**1ae**	-5.24	1.98E-03	5.69E-06	Moderately soluble
**1af**	-5.24	1.98E-03	5.69E-06	Moderately soluble
**1ag**	-5.24	1.98E-03	5.69E-06	Moderately soluble
**1ba**	-4.17	2.11E-02	6.72E-05	Moderately soluble
**1bb**	-4.11	2.21E-02	7.79E-05	Moderately soluble
**1bc**	-4.33	1.52E-02	4.65E-05	Moderately soluble
**1bd**	-4.95	3.95E-03	1.12E-05	Moderately soluble
**1be**	-5.01	3.52E-03	9.70E-06	Moderately soluble
**1bf**	-5.01	3.52E-03	9.70E-06	Moderately soluble
**1bg**	-5.01	3.52E-03	9.70E-06	Moderately soluble
**2aa**	-8.3	2.97E-06	5.00E-09	Poorly soluble
**2ab**	-8.14	3.88E-06	7.26E-09	Poorly soluble
**2ac**	-8.64	1.42E-06	2.29E-09	Poorly soluble
**2ad**	-9.89	8.61E-08	1.28E-10	Poorly soluble
**2ae**	-9.97	7.50E-08	1.08E-10	Poorly soluble
**2af**	-9.97	7.50E-08	1.08E-10	Poorly soluble
**2ag**	-9.97	7.50E-08	1.08E-10	Poorly soluble
**2ba**	-7.85	8.80E-06	1.41E-08	Poorly soluble
**2bb**	-7.69	1.17E-05	2.06E-08	Poorly soluble
**2bc**	-8.19	4.18E-06	6.41E-09	Poorly soluble
**2bd**	-9.45	2.52E-07	3.59E-10	Poorly soluble
**2be**	-9.52	2.21E-07	3.05E-10	Poorly soluble
**2bf**	-9.52	2.21E-07	3.05E-10	Poorly soluble
**2bg**	-9.52	2.21E-07	3.05E-10	Poorly soluble

Table S14. SwissADME physicochemical, pharmacokinetics and drug-likeness predictions for chalchones 1aa–1bg and bichalcone 2aa–2bg – part 6.

Molecule	Ali Log S	Ali Solubility (mg/ml)	Ali Solubility (mol/l)	Ali Class
**1aa**	-5.07	2.54E-03	8.51E-06	Moderately soluble
**1ab**	-4.91	3.32E-03	1.24E-05	Moderately soluble
**1ac**	-5.11	2.42E-03	7.76E-06	Moderately soluble
**1ad**	-5.83	4.96E-04	1.48E-06	Moderately soluble
**1ae**	-5.62	8.26E-04	2.38E-06	Moderately soluble
**1af**	-5.62	8.26E-04	2.38E-06	Moderately soluble
**1ag**	-5.62	8.26E-04	2.38E-06	Moderately soluble
**1ba**	-4.86	4.35E-03	1.38E-05	Moderately soluble
**1bb**	-4.7	5.72E-03	2.01E-05	Moderately soluble
**1bc**	-4.89	4.23E-03	1.29E-05	Moderately soluble
**1bd**	-5.61	8.65E-04	2.46E-06	Moderately soluble
**1be**	-5.41	1.40E-03	3.87E-06	Moderately soluble
**1bf**	-5.41	1.40E-03	3.87E-06	Moderately soluble
**1bg**	-5.41	1.40E-03	3.87E-06	Moderately soluble
**2aa**	-10.29	3.06E-08	5.15E-11	Insoluble
**2ab**	-9.96	5.83E-08	1.09E-10	Poorly soluble
**2ac**	-10.36	2.72E-08	4.38E-11	Insoluble
**2ad**	-11.8	1.06E-09	1.59E-12	Insoluble
**2ae**	-11.39	2.79E-09	4.03E-12	Insoluble
**2af**	-11.39	2.79E-09	4.03E-12	Insoluble
**2ag**	-11.39	2.79E-09	4.03E-12	Insoluble
**2ba**	-9.86	8.73E-08	1.39E-10	Poorly soluble
**2bb**	-9.53	1.67E-07	2.95E-10	Poorly soluble
**2bc**	-9.93	7.74E-08	1.19E-10	Poorly soluble
**2bd**	-11.37	3.02E-09	4.29E-12	Insoluble
**2be**	-10.96	7.90E-09	1.09E-11	Insoluble
**2bf**	-10.96	7.90E-09	1.09E-11	Insoluble
**2bg**	-10.96	7.90E-09	1.09E-11	Insoluble

Table S15. SwissADME physicochemical, pharmacokinetics and drug-likeness predictions for chalchones 1aa–1bg and bichalcone 2aa–2bg – part 7.

Molecule	Silicos-IT LogSw	Silicos-IT Solubility (mg/ml)	Silicos-IT Solubility (mol/l)	Silicos-IT class
**1aa**	-5.01	2.91E-03	9.75E-06	Moderately soluble
**1ab**	-4.89	3.42E-03	1.28E-05	Moderately soluble
**1ac**	-4.99	3.20E-03	1.03E-05	Moderately soluble
**1ad**	-5.75	5.99E-04	1.78E-06	Moderately soluble
**1ae**	-5.71	6.82E-04	1.96E-06	Moderately soluble
**1af**	-5.71	6.82E-04	1.96E-06	Moderately soluble
**1ag**	-5.71	6.82E-04	1.96E-06	Moderately soluble
**1ba**	-4.74	5.68E-03	1.81E-05	Moderately soluble
**1bb**	-4.63	6.68E-03	2.35E-05	Moderately soluble
**1bc**	-4.72	6.25E-03	1.91E-05	Moderately soluble
**1bd**	-5.48	1.17E-03	3.32E-06	Moderately soluble
**1be**	-5.44	1.33E-03	3.66E-06	Moderately soluble
**1bf**	-5.44	1.33E-03	3.66E-06	Moderately soluble
**1bg**	-5.44	1.33E-03	3.66E-06	Moderately soluble
**2aa**	-9.87	8.10E-08	1.36E-10	Poorly soluble
**2ab**	-9.69	1.10E-07	2.06E-10	Poorly soluble
**2ac**	-9.8	9.91E-08	1.60E-10	Poorly soluble
**2ad**	-11.28	3.54E-09	5.27E-12	Insoluble
**2ae**	-11.18	4.61E-09	6.65E-12	Insoluble
**2af**	-11.18	4.61E-09	6.65E-12	Insoluble
**2ag**	-11.18	4.61E-09	6.65E-12	Insoluble
**2ba**	-9.3	3.11E-07	4.97E-10	Poorly soluble
**2bb**	-9.13	4.22E-07	7.45E-10	Poorly soluble
**2bc**	-9.23	3.81E-07	5.83E-10	Poorly soluble
**2bd**	-10.71	1.36E-08	1.94E-11	Insoluble
**2be**	-10.61	1.78E-08	2.45E-11	Insoluble
**2bf**	-10.61	1.78E-08	2.45E-11	Insoluble
**2bg**	-10.61	1.78E-08	2.45E-11	Insoluble

Table S16. SwissADME physicochemical, pharmacokinetics and drug-likeness predictions for chalchones 1aa–1bg and bichalcone 2aa–2bg – part 8.

Molecule	GI absorption	BBB permeant	Pgp substrate	CYP1A2 inhibitor	CYP2C19 inhibitor
**1aa**	High	Yes	No	Yes	Yes
**1ab**	High	Yes	No	Yes	Yes
**1ac**	High	Yes	No	Yes	Yes
**1ad**	High	Yes	No	Yes	Yes
**1ae**	High	Yes	No	Yes	Yes
**1af**	High	Yes	No	Yes	Yes
**1ag**	High	Yes	No	Yes	Yes
**1ba**	High	Yes	No	Yes	Yes
**1bb**	High	Yes	No	Yes	Yes
**1bc**	High	Yes	No	Yes	Yes
**1bd**	High	Yes	No	Yes	Yes
**1be**	High	Yes	No	Yes	Yes
**1bf**	High	Yes	No	Yes	Yes
**1bg**	High	Yes	No	Yes	Yes
**2aa**	Low	No	No	No	No
**2ab**	Low	No	No	No	No
**2ac**	Low	No	No	No	No
**2ad**	Low	No	No	No	No
**2ae**	Low	No	No	No	No
**2af**	Low	No	No	No	No
**2ag**	Low	No	No	No	No
**2ba**	Low	No	No	No	Yes
**2bb**	Low	No	No	No	No
**2bc**	Low	No	No	No	Yes
**2bd**	Low	No	No	No	No
**2be**	Low	No	No	No	No
**2bf**	Low	No	No	No	No
**2bg**	Low	No	No	No	No

Table S17. SwissADME physicochemical, pharmacokinetics and drug-likeness predictions for chalchones 1aa–1bg and bichalcone 2aa–2bg – part 9.

Molecule	CYP2C9 inhibitor	CYP2D6 inhibitor	CYP3A4 inhibitor	log Kp (cm/s)	Lipinski #violations
**1aa**	Yes	Yes	Yes	-5.14	0
**1ab**	Yes	Yes	Yes	-4.94	0
**1ac**	Yes	Yes	Yes	-5.11	0
**1ad**	Yes	No	Yes	-4.72	0
**1ae**	Yes	Yes	Yes	-4.93	0
**1af**	Yes	Yes	Yes	-4.93	0
**1ag**	Yes	Yes	Yes	-4.93	0
**1ba**	Yes	No	Yes	-5.52	0
**1bb**	Yes	No	Yes	-5.31	0
**1bc**	Yes	Yes	Yes	-5.49	0
**1bd**	Yes	No	Yes	-5.1	0
**1be**	Yes	Yes	Yes	-5.31	0
**1bf**	Yes	Yes	Yes	-5.31	0
**1bg**	Yes	Yes	Yes	-5.31	0
**2aa**	No	No	No	-4.18	1
**2ab**	No	No	No	-3.77	1
**2ac**	No	No	No	-4.12	1
**2ad**	No	No	No	-3.35	2
**2ae**	No	Yes	No	-3.76	2
**2af**	No	Yes	No	-3.76	2
**2ag**	No	Yes	No	-3.76	2
**2ba**	No	No	No	-4.94	1
**2bb**	No	No	No	-4.53	1
**2bc**	No	No	No	-4.88	1
**2bd**	No	No	No	-4.1	1
**2be**	No	No	No	-4.51	1
**2bf**	No	No	No	-4.51	1
**2bg**	No	No	No	-4.51	1

Table S18. SwissADME physicochemical, pharmacokinetics and drug-likeness predictions for chalchones 1aa–1bg and bichalcone 2aa–2bg – part 10.

Molecule	Ghose #violations	Veber #violations	Egan #violations	Muegge #violations	Bioavailability Score
**1aa**	0	0	0	0	0.55
**1ab**	0	0	0	0	0.55
**1ac**	0	0	0	0	0.55
**1ad**	1	0	0	1	0.55
**1ae**	0	0	0	0	0.55
**1af**	0	0	0	0	0.55
**1ag**	0	0	0	0	0.55
**1ba**	0	0	0	0	0.55
**1bb**	0	0	0	0	0.55
**1bc**	0	0	0	0	0.55
**1bd**	0	0	0	0	0.55
**1be**	0	0	0	0	0.55
**1bf**	0	0	0	0	0.55
**1bg**	0	0	0	0	0.55
**2aa**	4	1	1	1	0.55
**2ab**	3	0	1	1	0.55
**2ac**	4	1	1	2	0.55
**2ad**	4	1	1	3	0.17
**2ae**	3	0	1	2	0.17
**2af**	3	0	1	2	0.17
**2ag**	3	0	1	2	0.17
**2ba**	4	1	1	2	0.55
**2bb**	4	1	1	1	0.55
**2bc**	4	1	1	2	0.55
**2bd**	4	1	1	3	0.55
**2be**	4	1	1	2	0.55
**2bf**	4	1	1	2	0.55
**2bg**	4	1	1	2	0.55

Table S19. SwissADME physicochemical, pharmacokinetics and drug-likeness predictions for chalchones 1aa–1bg and bichalcone 2aa–2bg – part 11.

Molecule	PAINS #alerts	Brenk #alerts	Leadlikeness #violations	Synthetic Accessibility
**1aa**	0	1	1	2.78
**1ab**	0	1	1	2.64
**1ac**	1	1	1	2.77
**1ad**	0	1	1	2.8
**1ae**	0	1	1	2.68
**1af**	0	1	1	2.74
**1ag**	0	1	1	2.77
**1ba**	0	1	1	2.87
**1bb**	0	1	1	2.73
**1bc**	1	1	1	2.86
**1bd**	0	1	2	2.87
**1be**	0	1	2	2.77
**1bf**	0	1	2	2.83
**1bg**	0	1	2	2.87
**2aa**	0	1	3	4.52
**2ab**	0	1	3	4.2
**2ac**	1	1	3	4.71
**2ad**	0	1	3	4.46
**2ae**	0	1	3	4.22
**2af**	0	1	3	4.26
**2ag**	0	1	3	4.28
**2ba**	0	1	3	4.7
**2bb**	0	1	3	4.38
**2bc**	1	1	3	4.87
**2bd**	0	1	3	4.61
**2be**	0	1	3	4.39
**2bf**	0	1	3	4.43
**2bg**	0	1	3	4.45

6 Bibliography

Gabriel, C.M., Lee, N.R., Bigorne, F., Klumphu, P., Parmentier, M., Gallou, F., et al. (2017). Effects of Co-solvents on Reactions Run under Micellar Catalysis Conditions. *Org. Lett.* 19(1), 194-197. doi: 10.1021/acs.orglett.6b03468.

Ganardi, R.C., Greb, J., Henssen, B. and Pietruszka, J. Atroposelective Total Synthesis of (+)-Isokotanin A via Combined Metal and Enzyme Catalysis. *Adv. Synth. Catal.*(365), 3512-3520. doi: 10.1002/adsc.202300698.

Greb, J., Drennhaus, T., Klischan, M.K.T., Schroeder, Z.W., Frey, W. and Pietruszka, J. (2023). A Common C2-Symmetric 2,2'-Biphenol Building Block and its Application in the Synthesis of (+)-di-epi-Gonytolide A. *Chemistry – A European Journal* 29(34), e202300941. doi: 10.1002/chem.202300941.

Klischan, M.K.T., Mazzone, F., Berning, L., Greb, J., Schlamkow, M., Haase, M., et al. (2023). Modular Approach for the Synthesis and Bioactivity Profiling of 8,8′-Biflavones. *ACS Omega* 8(44), 41816-41834. doi: 10.1021/acsomega.3c06503.

Lipshutz, B.H., Ghorai, S., Abela, A.R., Moser, R., Nishikata, T., Duplais, C., et al. (2011). TPGS-750-M: A Second-Generation Amphiphile for Metal-Catalyzed Cross-Couplings in Water at Room Temperature. *The Journal of Organic Chemistry* 76(11), 4379-4391. doi: 10.1021/jo101974u.

Pettersen, E.F., Goddard, T.D., Huang, C.C., Meng, E.C., Couch, G.S., Croll, T.I., et al. (2021). UCSF ChimeraX: Structure visualization for researchers, educators, and developers. *Protein Sci.* 30(1), 70-82. doi: 10.1002/pro.3943.

Rizzo, S., Benincori, T., Fontana, F., Pasini, D. and Cirilli, R. (2022). HPLC Enantioseparation of Rigid Chiral Probes with Central, Axial, Helical, and Planar Stereogenicity on an Amylose (3,5-Dimethylphenylcarbamate) Chiral Stationary Phase. *Molecules* 27(23), 8527. doi: 10.3390/molecules27238527.

Sechi, B., Mamane, V., Dallocchio, R., Dessì, A., Cossu, S., Jibuti, G., et al. (2023). Enantioseparation of new axially chiral carboxylic acids on polysaccharide-based chiral stationary phases under normal phase elution conditions. *Journal of Pharmaceutical and Biomedical Analysis Open* 1, 100011. doi: 10.1016/j.jpbao.2023.100011.

Tietze, L.F., Ma, L. and Jackenkroll, S. (2014). The paecilin puzzle - enantioselective syntheses of the proposed structures of paecilin A and B. *Heterocycles* 88(2), 1101-1119. doi: 10.3987/COM-13-S(S)68.

Wang, T. and Chen, Y.W. (1999). Application and comparison of derivatized cellulose and amylose chiral stationary phases for the separation of enantiomers of pharmaceutical compounds by high-performance liquid chromatography. *J. Chromatogr. A* 855(2), 411-421. doi: 10.1016/S0021-9673(99)00733-5.

Wang, T., Chen, Y.W. and Vailaya, A. (2000). Enantiomeric separation of some pharmaceutical intermediates and reversal of elution orders by high-performance liquid chromatography using

cellulose and amylose tris(3,5-dimethylphenylcarbamate) derivatives as stationary phases. *J. Chromatogr. A* 902(2), 345-355. doi: 10.1016/S0021-9673(00)00862-1.

Wang, T. and Wenslow, R.M. (2003). Effects of alcohol mobile-phase modifiers on the structure and chiral selectivity of amylose tris(3,5-dimethylphenylcarbamate) chiral stationary phase. *J. Chromatogr. A* 1015(1), 99-110. doi: 10.1016/S0021-9673(03)01262-7.

Yamamoto, C., Yashima, E. and Okamoto, Y. (2002). Structural Analysis of Amylose Tris(3,5-dimethylphenylcarbamate) by NMR Relevant to Its Chiral Recognition Mechanism in HPLC. *Journal of the American Chemical Society* 124(42), 12583-12589. doi: 10.1021/ja020828g.

Ye, Y.K., Bai, S., Vyas, S. and Wirth, M.J. (2007). NMR and Computational Studies of Chiral Discrimination by Amylose Tris(3,5-dimethylphenylcarbamate). *The Journal of Physical Chemistry B* 111(5), 1189-1198. doi: 10.1021/jp0637173.
